# Asymptomatic Hemorrhagic Events and Functional Outcomes in Acute Stroke: A Secondary Analysis of the DIRECT-MT Randomized Clinical Trial

**DOI:** 10.1001/jamanetworkopen.2025.2411

**Published:** 2025-03-28

**Authors:** Rundong Chen, Weilong Hua, Yilei Zhang, Yongxin Zhang, Hongjian Zhang, Yongwei Zhang, Jianmin Liu, Pengfei Yang, Xiaoxi Zhang, Lei Zhang

**Affiliations:** 1 School of Health Science and Engineering, University of Shanghai for Science and Technology, Shanghai, China; 2 Neurovascular Center, Changhai Hospital, Naval Medical University, Shanghai, China; 3 Nursing Department, Sir Run Run Shaw Hospital, Zhejiang University School of Medicine, Hangzhou, China; 4 Oriental Pan-Vascular Devices Innovation College, University of Shanghai for Science and Technology, Shanghai, China; 5 State Key Laboratory of Medical Neurobiology and MOE Frontiers Center for Brain Science, Institutes of Brain Science, Shanghai Medical College, Fudan University, Shanghai, China

## Abstract

**Importance:**

Asymptomatic hemorrhagic infarction (HI) and subarachnoid hemorrhage (SAH) after endovascular treatment (EVT) for acute ischemic stroke are commonly considered low risk, but their long-term impact on functional outcomes is unclear.

**Objective:**

To determine whether asymptomatic HI and SAH are associated with worse 90-day functional recovery in patients with acute ischemic stroke treated with EVT.

**Design, Setting, and Participants:**

This is a secondary analysis of the DIRECT-MT randomized clinical trial, which compared intravenous thrombolysis prior to EVT with EVT alone. The multicenter study was conducted at tertiary hospitals in China between 2016 and 2019 with 90-day follow-up. Trial patients with asymptomatic HI, SAH, or no hemorrhage were included in the present analysis, which was performed in December 2024.

**Exposure:**

Asymptomatic HI and SAH detected on follow-up imaging.

**Main Outcomes and Measures:**

The primary outcome was the score on the modified Rankin scale (mRS) assessed at 90 days. Secondary analyses categorized mRS scores into thresholds of 0 to 1, 0 to 2, and 0 to 3, representing excellent, good, and favorable recovery, respectively.

**Results:**

A total of 490 patients were included (median [IQR] age, 70 [60-76] years; 210 [42.9%] female), with 133 (27.1%) in the asymptomatic HI and SAH group and 357 (72.9%) in the no hemorrhage group. After propensity score matching, the odds ratio of having a worse mRS scores at 90 days in the asymptomatic HI and SAH group compared with the no hemorrhage group was 2.59 (95% CI, 1.45-4.63; *P* = .001). For binary outcomes, asymptomatic HI and SAH were consistently associated with worse recovery across mRS score thresholds of 0 to 1 and 0 to 2 in all models.

**Conclusions and Relevance:**

In this secondary analysis of a randomized clinical trial, asymptomatic HI and SAH were associated with worse 90-day functional outcomes in patients with acute ischemic stroke treated with EVT. These findings emphasize the need for close monitoring and tailored management strategies in patients with asymptomatic hemorrhagic events following thrombectomy.

**Trial Registration:**

ClinicalTrials.gov Identifier: NCT03469206

## Key Points

**Question:**

Do asymptomatic hemorrhagic infarction (HI) and subarachnoid hemorrhage (SAH) after endovascular treatment for acute ischemic stroke affect long-term functional outcomes?

**Findings:**

In this secondary analysis of a randomized clinical trial with 490 participants, asymptomatic HI and SAH were significantly associated with worse 90-day outcomes after propensity score matching.

**Meaning:**

In this study, asymptomatic HI and SAH were negatively associated with stroke recovery, suggesting a need for closer monitoring and proactive management to improve outcomes.

## Teaser

This secondary analysis of a randomized clinical trial evaluates whether asymptomatic hemorrhagic infarction and subarachnoid hemorrhage, often considered low risk, are associated with 90-day outcomes among patients in China.

## Introduction

Intracranial hemorrhage (ICH) is a known complication following endovascular treatment (EVT) for acute ischemic stroke (AIS).^1^ While symptomatic ICH, which results in clinical deterioration, is well-recognized for its association with poor outcomes, there is much less focus on asymptomatic ICH—hemorrhages that are radiologically detected but do not immediately affect neurological status.^2^ This has led to a clinical assumption that asymptomatic ICH is largely benign, especially when involving small volumes of blood, as in hemorrhagic infarction type 1 (HI1), hemorrhagic infarction type 2 (HI2), and subarachnoid hemorrhage (SAH).^3^

Although research comparing outcomes between patients with symptomatic ICH and those with no hemorrhage exists,^4,5^ studies specifically addressing the prognostic significance of asymptomatic HI and SAH are limited. Clinicians often regard asymptomatic HI and SAH, particularly those without overt clinical symptoms, as inconsequential. As a result, these asymptomatic hemorrhages are frequently overlooked in both acute management and long-term follow-up, under the assumption that they do not negatively impact patient recovery.

In this study, we utilized data from the Direct-MT trial to perform an in-depth analysis of patients with HI1, HI2, or SAH hemorrhages that were classified as asymptomatic. Our goal was to investigate whether these patients, despite lacking immediate clinical symptoms, had worse functional outcomes at 90 days compared with patients without any hemorrhage. By focusing on these often-overlooked hemorrhages, we aim to challenge the prevailing view that asymptomatic HI and SAH can be disregarded in clinical practice.

## Methods

This study used data from the Direct-MT trial—a multicenter, open-label randomized clinical trial conducted across tertiary hospitals between 2016 and 2019 in China. The trial compared the efficacy of EVT alone vs combined intravenous thrombolysis and EVT in patients with AIS due to large-vessel occlusion. This study’s protocol aligns with the Consolidated Standards of Reporting Trials (CONSORT) standards for reporting randomized clinical trials. The study protocol (Supplement 1), including procedures for obtaining written informed consent from all participants or their legal representatives, was approved by the relevant ethics committees and has been previously published.^6^ For this secondary analysis, additional informed consent was not required, as only deidentified data were used.

The classification of hemorrhage subtypes followed the Heidelberg Bleeding Classification. All imaging was evaluated by an independent core laboratory that was blinded to treatment allocation. Asymptomatic HI and SAH were defined as HI1, HI2, or SAH, provided that they also met the criteria for asymptomatic hemorrhage according to the Heidelberg Bleeding Classification. Patients with other types of hemorrhagic transformation or symptomatic ICH were excluded from the analysis. The control group consisted of patients without any hemorrhage on follow-up imaging.

Baseline demographic and clinical data were collected, including age, sex, body weight, systolic blood pressure, and serum glucose levels. The etiology of the stroke was categorized as cardioembolism, intracranial atherosclerosis, or undetermined cause. Medical history was also recorded, including prior ischemic stroke, atrial fibrillation, diabetes, and hypertension, as well as the use of preadmission medications, such as antiplatelet agents, vitamin K antagonists, direct oral anticoagulants, and statins.

Stroke severity was assessed at baseline using the National Institutes of Health Stroke Scale (NIHSS),^7^ and prestroke functional status was evaluated using the modified Rankin Scale (mRS).^8^ Early ischemic changes were assessed using the Alberta Stroke Program Early Computed Tomography Score (ASPECTS) on baseline noncontrast computed tomography. Additionally, the presence of leukoaraiosis and the location of intracranial target occlusions were documented. Procedural details, such as the number of mechanical thrombectomy attempts, time from stroke onset to reperfusion, and the use of periprocedural medications, including heparin and glycoprotein IIb/IIIa–receptor antagonists, were also recorded. Reperfusion success was defined as achieving an expanded Thrombolysis in Cerebral Infarction score of 2b or better.^9^

The primary outcome was the functional outcome at 90 days, initially analyzed as a continuous ordinal variable to capture the full range of modified mRS scores. To enhance clinical interpretability, we further categorized mRS scores into binary thresholds of 0 to 1, 0 to 2, and 0 to 3, representing excellent, good, and favorable outcomes, respectively.

### Statistical Analysis

Descriptive statistics were used to summarize baseline characteristics, outcomes, and procedural variables. Continuous variables are expressed as means with SDs or medians with IQRs, depending on the data distribution, while categorical variables are presented as frequencies and percentages. Group comparisons were conducted using the χ^2^ test or Fisher exact test for categorical variables, and the Student *t* test or Mann-Whitney *U* test for continuous variables, depending on the distribution of the data. Only 11 cases contained missing values across the covariates. These cases were excluded using a complete-case analysis approach, and no imputation methods were applied.

To control for potential confounders and improve comparability between groups, we used propensity score–based methods, including propensity score matching (PSM) and inverse probability of treatment weighting (IPTW). Propensity scores for experiencing asymptomatic HI and SAH were estimated using a multivariable logistic regression model that incorporated all baseline covariates, including demographic characteristics, clinical data, imaging findings, and procedural details. These scores were used to create matched pairs in the PSM algorithm, which implements nearest neighbor matching with a 1:1 ratio and a caliper width of 0.01, and as weights in the IPTW model. We calculated standardized mean differences (SMDs) to evaluate the effectiveness of PSM in balancing covariates between groups.

To analyze the primary outcome, we applied ordinal regression analyses on the propensity score–matched dataset, treating the mRS score as a continuous ordinal variable. Next, to provide clinically meaningful insights, we categorized mRS scores into binary outcomes (0-1, 0-2, and 0-3) to represent excellent, good, and favorable outcomes at 90 days, respectively. For these binary outcomes, we used the McNemar test to compare paired outcomes in the propensity score–matched dataset. Multivariable logistic regression was used to further adjust for all baseline covariates, providing adjusted odds ratios (ORs) with 95% CIs. An IPTW model was then developed using the estimated propensity scores as weights. After weighting, logistic regression models were applied to the weighted cohort using the glm function from the stats package of R version 4.3.0 (R Project for Statistical Computing) to estimate adjusted ORs.

To increase the robustness of the analysis, we also used doubly robust estimation, which combines both multivariable regression models and propensity score models. This method has been used in several previously published studies with well-designed methods.^10,11^ These studies not only demonstrated the effectiveness of the approach but also ensured transparency and reproducibility by providing open access to their data, statistical methods, and code. In our analysis, the doubly robust model was also applied in 2 forms: one adjusting for all covariates and the other adjusting only for unbalanced covariates after matching. We first applied the svydesign function in survey package to incorporate the IPTW into a survey design object, ensuring proper handling of the weighted pseudo-population. The svyglm function was then used to fit logistic regression models, adjusting for covariates. This allowed us to estimate ORs with 95% CIs, while accounting for the weights and covariates between groups.

For sensitivity analysis, all covariates were included in the model, and continuous variables were categorized. Subgroup analyses were conducted to explore interactions between variables and determine whether certain subpopulations demonstrated different outcomes. Interaction *P* values were calculated to assess potential effect modifications and to verify the robustness of the results across various subgroups.

All statistical analyses were performed using R software version 4.3.0, with a significance threshold of *P* < .05. The current analysis was performed in December 2024.

## Results

**Figure 1.**
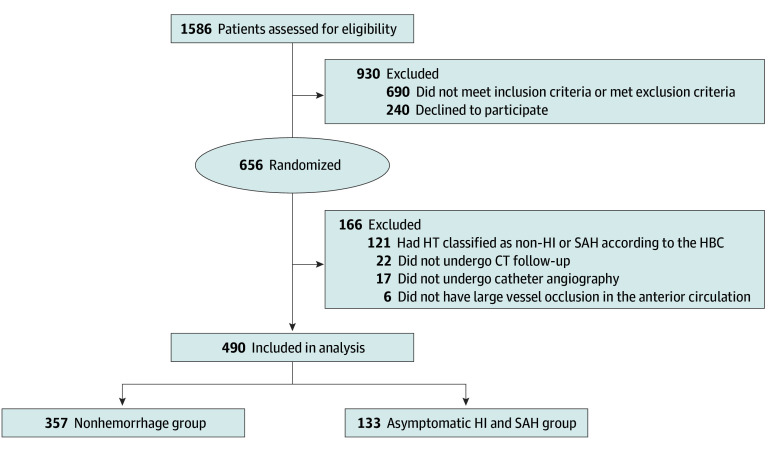
Flowchart of Patient Selection and Group Allocation CT indicates computed tomography; HBC, Heidelberg Bleeding Classification; HI, hemorrhagic infarction; HT, hemorrhagic transformation; and SAH, subarachnoid hemorrhage.

A total of 490 patients were included in the study (median [IQR] age, 70 [60-76] years; 210 [42.9%] female), with 133 (27.1%) patients in the asymptomatic HI and SAH group and 357 (72.9%) patients in the no hemorrhage group (Figure 1). Baseline characteristics before and after PSM are summarized in Table 1. Prior to PSM, several significant differences were observed between the asymptomatic HI and SAH and no hemorrhage groups. The asymptomatic HI and SAH group had higher median (IQR) NIHSS scores (18 [14-23] vs 16 [12-20]) and lower median (IQR) ASPECTS scores (8 [7-9] vs 9 [7-10]). They also had higher median (IQR) glucose levels (129.73 [109.91-184.32] mg/dL vs 120.72 [104.32-142.34] mg/dL [to convert to millimoles per liter, multiply by 0.0555]) and a longer median (IQR) time from stroke onset to reperfusion (300.00 [256.00-341.00] minutes vs 264.00 [213.75-311.00] minutes). Additionally, the asymptomatic HI and SAH group underwent more median (IQR) thrombectomy attempts (2 [1-3] vs 1 [1-2]) and had poorer 90-day functional outcomes.

**Table 1.** Baseline Characteristics Before and After Propensity Score Matching of 2 Cohorts

Characteristic	Before matching					After matching				
	Patients, No. (%)			SMD	*P* value	Patients. No. (%)			SMD	*P* value
	Overall (N = 490)	Asymptomatic HI and SAH (n = 133)	No hemorrhage (n = 357)			Overall (n = 160)	Asymptomatic HI and SAH (n = 80)	No hemorrhage (n = 80)		
**Demographic data**										
Age, median (IQR), y	70.00 (60.00-76.00)	69.00 (60.00-75.00)	70.00 (60.00-76.00)	0.040	.44	68.00 (60.00-77.00)	68.00 (60.00-76.25)	69.00 (59.50-77.00)	0.030	.73
Sex										
Female	210 (42.86)	51 (38.35)	159 (44.54)	0.126	.26	70 (43.75)	32 (40.00)	38 (47.50)	0.152	.43
Male	280 (57.14)	82 (61.65)	198 (55.46)			90 (56.25)	48 (60.00)	42 (52.50)		
Weight, median (IQR), kg	65.00 (58.00-74.00)	65.00 (60.00-75.00)	65.00 (56.00-72.00)	0.144	.21	65.52 (10.84)	65.81 (10.60)	65.23 (11.13)	0.054	.73
Systolic blood pressure, median (IQR), mm Hg	145.00 (131.00-160.75)	143.00 (132.00-158.00)	146.00 (131.00-163.00)	0.154	.17	143.24 (21.30)	143.85 (20.42)	142.62 (22.25)	0.058	.72
Glucose, median (IQR), mg/dL	122.52 (104.50-150.81)	129.73 (109.91-184.32)	120.72 (104.32-142.34)	0.394	.002	123.60 (108.29-159.46)	125.23 (107.03-163.42)	121.98 (108.47-159.28)	0.074	.80
Cause of stroke										
Cardioembolic	213 (43.47)	56 (42.11)	157 (43.98)	0.098	.37	71 (44.38)	34 (42.50)	37 (46.25)	0.082	.96
Intracranial atherosclerosis	37 (7.55)	6 (4.51)	31 (8.68)			10 (6.25)	5 (6.25)	5 (6.25)		
Ipsilateral extracranial ICA obstruction	50 (10.20)	14 (10.53)	36 (10.08)			20 (12.50)	10 (12.50)	10 (12.50)		
Undetermined	190 (38.78)	57 (42.86)	133 (37.25)			59 (36.88)	31 (38.75)	28 (35.00)		
**Medical history**										
Previous ischemic stroke	71 (14.49)	24 (18.05)	47 (13.17)	0.135	.22	30 (18.75)	12 (15.00)	18 (22.50)	0.193	.31
History of atrial fibrillation	222 (45.31)	59 (44.36)	163 (45.66)	0.026	.88	75 (46.88)	36 (45.00)	39 (48.75)	0.075	.75
History of diabetes	83 (16.94)	28 (21.05)	55 (15.41)	0.147	.18	30 (18.75)	17 (21.25)	13 (16.25)	0.128	.54
History of hypertension	287 (58.57)	74 (55.64)	213 (59.66)	0.082	.48	89 (55.62)	45 (56.25)	44 (55.00)	0.025	1
Use of antiplatelet medication	81 (16.53)	23 (17.29)	58 (16.25)	0.028	.89	29 (18.12)	15 (18.75)	14 (17.50)	0.032	1
Use of vitamin K antagonists	19 (3.88)	6 (4.51)	13 (3.64)	0.044	.86	10 (6.25)	4 (5.00)	6 (7.50)	0.103	.74
Use of direct oral anticoagulants	20 (4.08)	8 (6.02)	12 (3.36)	0.126	.29	5 (3.12)	2 (2.50)	3 (3.75)	0.072	1
Use of statins	34 (6.94)	12 (9.02)	22 (6.16)	0.108	.36	16 (10.00)	7 (8.75)	9 (11.25)	0.083	.79
**Baseline assessments**										
NIHSS score, median (IQR)^a^	17.00 (13.00-21.00)	18.00 (14.00-23.00)	16.00 (12.00-20.00)	0.284	.01	17.00 (13.00-22.00)	16.00 (13.00-22.00)	18.00 (14.00-22.00)	0.198	.15
Modified Rankin Scale score of 1 or 2^b^	42 (8.57)	10 (7.52)	32 (8.96)	0.053	.74	15 (9.38)	7 (8.75)	8 (10.00)	0.043	1
ASPECTS, median (IQR)^c^	9.00 (7.00-10.00)	8.00 (7.00-9.00)	9.00 (7.00-10.00)	0.275	.003	8.50 (7.00-10.00)	8.00 (7.00-9.00)	9.00 (7.00-10.00)	0.018	.69
Leukoaraiosis	163 (33.27)	43 (32.33)	120 (33.61)	0.021	.92	49 (30.62)	23 (28.75)	26 (32.50)	0.081	.73
**Treatment specifics**										
Intracranial target occlusion location^d^										
Intracranial ICA	150 (30.61)	44 (33.08)	106 (29.69)	0.108	.56	52 (32.50)	24 (30.00)	28 (35.00)	0.101	.79
M1	284 (57.96)	76 (57.14)	208 (58.26)			91 (56.88)	47 (58.75)	44 (55.00)		
M2	55 (11.22)	12 (9.02)	43 (12.04)			17 (10.62)	9 (11.25)	8 (10.00)		
Intervention therapy (direct EVT)	255 (52.04)	74 (55.64)	181 (50.70)	0.099	.38	73 (45.62)	42 (52.50)	31 (38.75)	0.279	.11
Per-procedural heparin	214 (43.67)	60 (45.11)	154 (43.14)	0.037	.79	69 (43.12)	38 (47.50)	31 (38.75)	0.177	.34
Per-procedural GPIIb/IIIa–receptor antagonist	142 (28.98)	35 (26.32)	107 (29.97)	0.083	.48	39 (24.38)	19 (23.75)	20 (25.00)	0.029	1
Mechanical thrombectomy attempts, median (IQR), No.	2.00 (1.00-3.00)	2.00 (1.00-3.00)	1.00 (1.00-2.00)	0.489	<.001	2.00 (1.00-3.00)	2.00 (1.00-3.00)	2.00 (1.00-3.00)	0.132	.62
Time from stroke onset to reperfusion, median (IQR), min^e^	271.00 (225.00-324.00)	300.00 (256.00-341.00)	264.00 (213.75-311.00)	0.378	<.001	275.50 (230.00-325.25)	284.50 (250.00-322.75)	263.00 (221.25-328.00)	0.230	.14
Reperfusion on final DSA (successful reperfusion)^f^	406 (82.86)	104 (78.20)	302 (84.59)	0.165	.12	136 (85.00)	65 (81.25)	71 (88.75)	0.211	.27
**Outcome**										
Modified Rankin scale score at 90 d (0 to 1)^b^	138 (28.16)	16 (12.03)	122 (34.17)	0.544	<.001	32 (20.00)	10 (12.50)	22 (27.50)	0.382	<.05
Modified Rankin scale score at 90 d (0 to 2)^b^	209 (42.65)	29 (21.80)	180 (50.42)	0.624	<.001	51 (31.88)	16 (20.00)	35 (43.75)	0.527	<.01
Modified Rankin scale score at 90 d (0 to 3)^b^	301 (61.43)	59 (44.36)	242 (67.79)	0.486	<.001	89 (55.62)	39 (48.75)	50 (62.50)	0.279	.08

Abbreviations: ASPECTS, Alberta Stroke Program Early Computed Tomography Score; DSA, digital subtraction angiography; EVT, endovascular treatment; GPIIb/IIIa, glycoprotein IIb/IIIa; HI, hemorrhagic infarction; ICA, internal carotid artery; NIHSS, National Institutes of Health Stroke Scale; SAH, subarachnoid hemorrhage; SMD, standardized mean difference.

SI conversion factor: To convert glucose to millimoles per liter, multiply by 0.0555.

^a^ Scores on the NIHSS range from 0 to 42, with higher scores indicating more severe neurologic deficits.

^b^ Scores on the modified Rankin Scale of functional recovery range from 0 (no symptoms) to 6 (death).

^c^ The ASPECTS is a measure of the extent of early cerebral ischemia. Scores range from 0 to 10, with higher scores indicating fewer early ischemic changes. Shown are values as assessed by the core laboratory.

^d^ The cause of stroke was assessed according to the medical history, clinical features, and results on digital subtraction angiography.

^e^ Reperfusion was defined as the first visualization of successful reperfusion, as indicated by an extended Thrombolysis in Cerebral Infarction eTICI score of 2b, 2c, or 3 (on a scale from 0 [no reperfusion] to 3 [complete reperfusion]).

^f^ An extended Thrombolysis in Cerebral Infarction score of 2b, 2c, or 3 indicated successful reperfusion.

**Figure 2.**
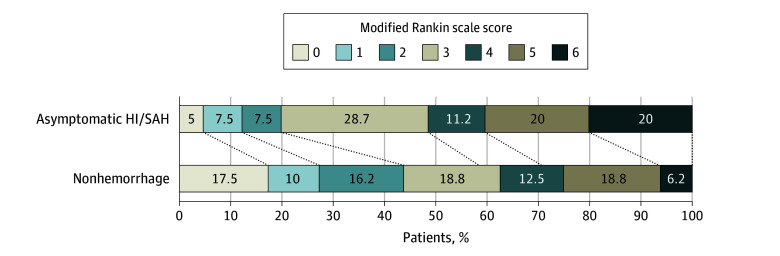
Distribution of Functional Outcomes at 90 Days in Asymptomatic Hemorrhagic Infarction (HI) and Subarachnoid Hemorrhage Group (SAH) and No Hemorrhage Groups Scores range from 0 to 6, with 0 indicating no symptoms; 1, no clinically significant disability; 2, slight disability (patients are able to look after their own affairs without assistance but are unable to carry out all previous activities); 3, moderate disability (patients require some help but are able to walk unassisted); 4, moderately severe disability (patients are unable to attend to bodily needs without assistance and are unable to walk unassisted); 5, severe disability (patients require constant nursing care and attention); and 6, death.

After PSM, Figure 2 shows the distribution of mRS scores for the asymptomatic HI and SAH and non-hemorrhage groups. The OR for higher mRS score at 90 days among patients with asymptomatic HI and SAH compared with those with no hemorrhage was 2.59 (95% CI, 1.45-4.63; *P* = .001).

Five models were applied to assess the association between asymptomatic HI and SAH and 90-day outcomes (mRS 0-1, 0-2, and 0-3), summarized in Table 2. For mRS scores 0 to 1, PSM showed an OR of 0.37 (95% CI, 0.15-0.88; *P* = .03), and the multivariable model gave an OR of 0.31 (95% CI, 0.15-0.61; *P* < .001). IPTW and doubly robust models confirmed these findings, with the doubly robust with unbalanced covariates model showing an OR of 0.33 (95% CI, 0.17-0.65; *P* = .002). For mRS scores 0 to 2, PSM showed an OR of 0.30 (95% CI, 0.13-0.65; *P* = .002), with similar results from IPTW and doubly robust models. The doubly robust with unbalanced covariates model yielded an OR of 0.31 (95% CI, 0.17-0.55; *P* < .001). For mRS scores 0 to 3, PSM resulted in an OR of 0.56 (95% CI, 0.29-1.08; *P* = .11), while multivariable and IPTW models showed significant associations. The doubly robust with unbalanced covariates model for mRS scores 0 to 3 produced an OR of 0.43 (95% CI, 0.25-0.75; *P* = .003).

**Table 2.** Association Between Asymptomatic HI and SAH and 90-Day mRS Scores of 0 to 1, 0 to 2, and 0 to 3 Across Different Models

Model	Discordant pairs, No.	Patients, No./total No. (%)		OR (95% CI)	*P* value
		No hemorrhage	Asymptomatic HI and SAH		
**mRS 0-1**					
Propensity score matching	26	22/80 (27.50)	10/80 (12.50)	0.37 (0.15-0.88)	.03
Multivariate logistic model adjusted with all covariates		122/357 (34.17)	16/133 (12.03)	0.31 (0.15-0.61)	<.001
Propensity score IPTW		151/485 (31.13)	61/456 (13.38)	0.30 (0.20-0.44)	<.001
Doubly robust estimation with all covariates		151/485 (31.13)	61/456 (13.38)	0.30 (0.15-0.60)	<.001
Doubly robust estimation with unbalanced covariates		151/485 (31.13)	61/456 (13.38)	0.33 (0.17-0.65)	.002
**mRS 0-2**					
Propensity score matching	35	35/80 (43.75)	16/80 (20.00)	0.30 (0.13-0.65)	.002
Multivariate logistic model adjusted with all covariates		180/357 (50.42)	29/133 (21.80)	0.30 (0.16-0.52)	<.001
Propensity score IPTW		228/485 (47.01)	109/456 (23.90)	0.27 (0.19-0.38)	<.001
Doubly robust estimation with all covariates		228/485 (47.01)	109/456 (23.90)	0.27 (0.15-0.51)	<.001
Doubly robust estimation with unbalanced covariates		228/485 (47.01)	109/456 (23.90)	0.31 (0.17-0.55)	<.001
**mRS 0-3**					
Propensity score matching	39	50/80 (62.50)	39/80 (48.75)	0.56 (0.29-1.08)	.11
Multivariate logistic model adjusted with all covariates		242/357 (67.79)	59/133 (44.36)	0.45 (0.26-0.77)	.004
Propensity score IPTW		308/485 (63.51)	220/456 (48.25)	0.41 (0.29-0.56)	<.001
Doubly robust estimation with all covariates		308/485 (63.51)	220/456 (48.25)	0.41 (0.23-0.72)	.002
Doubly robust estimation with unbalanced covariates		308/485 (63.51)	220/456 (48.25)	0.43 (0.25-0.75)	.003

Abbreviations: HI, hemorrhagic infarction; IPTW, inverse probability of treatment weighting; SAH, subarachnoid hemorrhage; mRS, modified Rankin Scale; OR, odds ratio.

As part of the sensitivity analysis, forest plots were generated to assess mRS scores of 0 to 1 and mRS scores of 0 to 2 across all covariate subgroups. In the analysis of mRS scores of 0 to 1 (eFigure 1 in Supplement 2), male patients were more negatively affected by asymptomatic HI and SAH compared with female patients, and those with a single thrombectomy attempt were more adversely affected than those with multiple attempts (*P* for interaction = .01). For mRS scores of 0 to 2 (eFigure 2 in Supplement 2), patients with ASPECTS scores of 8 to 10 were more vulnerable to the detrimental effects of asymptomatic HI and SAH compared with those with scores of 0 to 7 (*P* for interaction = .02).

## Discussion

In this study, we investigated the association of asymptomatic HI and SAH following EVT for AIS with 90-day functional outcomes. In clinical practice, asymptomatic HI and SAH are typically regarded as low risk because of their limited volume and the tendency for these hemorrhages to be reabsorbed within a few days after the procedure. Since these hemorrhages typically do not cause immediate symptoms, they are frequently regarded as benign and often overlooked in follow-up care. However, our findings challenge this assumption, as we observed that asymptomatic HI and SAH were associated with significantly worse functional outcomes at 90 days, despite the absence of immediate neurological deterioration.

Several studies have explored the impact of asymptomatic ICH on outcomes in patients with ischemic stroke undergoing EVT, with mixed results. Feldman et al^12^ found no significant association between asymptomatic ICH and 90-day outcomes in 485 patients with stroke treated with EVT; other studies consistently highlight its detrimental impact on recovery (mRS 0-2: OR, 0.84; 95% CI, 0.53-1.35; *P* = .55). In contrast, several other studies have underscored the significant impact of asymptomatic ICH on functional outcomes. For instance, Constant Dit Beaufils et al^13^ analyzed 1526 patients treated for large-vessel occlusion and found that 42.7% developed asymptomatic ICH, leading to significantly worse outcomes at 3 months (mRS 4-6) compared with those without asymptomatic ICH (46.4% vs 30.8%; *P* < .01). Similarly, Kang et al^14^ examined 460 patients with large-vessel occlusion and found that 33% developed asymptomatic ICH, resulting in poorer functional recovery at 90 days (mRS ≤2: 32.2% vs 49.4%; *P* < .001). Additionally, Kr et al^5^ found hemorrhagic transformation in 222 of 478 patients, with HI2 and parenchymal hematoma type 2 being significantly associated with poorer outcomes (adjusted ORs, 0.54 and 0.37, respectively). A meta-analysis by Tang et al^15^ involving 10 915 patients confirmed that asymptomatic ICH was significantly associated with increased risk of poor functional outcomes at 3 months (adjusted ORs of 1.70 and 1.43), although no significant association with mortality was observed. Furthermore, Suzuki et al^16^ reported that 30% of 349 patients with stroke treated with mechanical thrombectomy developed asymptomatic ICH, with these patients showing notably worse outcomes at 90 days (30% vs 67%; *P* < .01). Zhang et al^4^ observed similar findings in 732 patients undergoing EVT, with 29.5% developing asymptomatic ICH, resulting in worse functional outcomes at 90 days (mRS 0-2: 37.8% vs 55.5%; *P* < .001). Factors such as age, NIHSS scores, ASPECTS, and the use of general anesthesia were also associated with outcomes in these patients. Collectively, these studies highlight the detrimental impact of asymptomatic ICH on stroke recovery, emphasizing the importance of mitigating its occurrence to improve patient outcomes.

While these studies provide valuable insights into the consequences of asymptomatic ICH, most rely on broader definitions of hemorrhage and do not offer a granular analysis of the different hemorrhagic subtypes. Clinically, parenchymal hematomas are treated with caution due to their association with clinical deterioration, prompting early treatment adjustments to prevent further complications. In contrast, smaller hemorrhages, such as HI and SAH, once confirmed stable on follow-up computed tomography, are often considered benign and do not lead to further intervention. This assumption, however, overlooks their potential impact on long-term outcomes. Our study challenges this perspective by providing a more detailed analysis of these smaller hemorrhages and their significant associations with recovery, suggesting a need for closer monitoring even when these hemorrhages are asymptomatic.

Unlike previous research, which primarily relied on observational designs, our study draws from high-quality, multicenter randomized clinical trial data (DIRECT-MT trial), which reduces selection bias and enhances the reliability of our conclusions. Additionally, we used advanced statistical methods—PSM, IPTW, and doubly robust estimation—to ensure thorough adjustment for confounders including stroke severity, baseline imaging, and treatment parameters. These methods provide more accurate comparisons between asymptomatic HI and SAH and no hemorrhage groups, reducing the potential for bias. Moreover, our study evaluates functional outcomes across multiple thresholds (mRS scores of 0-1, 0-2, and 0-3) and includes detailed procedural and imaging data, offering deeper insights into the factors associated with asymptomatic HI and SAH and their impact. This comprehensive approach allows us to better understand the mechanisms through which asymptomatic HI and SAH could affect recovery, highlighting the need for a reevaluation of how these hemorrhages are managed in clinical practice.

The worse outcomes associated with asymptomatic HI and SAH, despite the absence of immediate symptoms, may be explained by several complex mechanisms. Asymptomatic HI and SAH, although not causing immediate clinical deterioration, may disrupt neural pathways via axonal shearing, localized edema, and microvascular damage, leading to functional deficits during long-term recovery.^2,17^ This disruption, while not causing immediate symptoms, accumulates over time, eventually leading to functional deficits that emerge later in the recovery process. As such, the true extent of the damage caused by asymptomatic HI and SAH is often underestimated during the initial phase of stroke management, only becoming apparent during longer-term recovery.

Asymptomatic HI and SAH may reflect a compromised blood-brain barrier due to chronic conditions like hypertension or diabetes, allowing blood leakage and microvascular damage.^18,19^ These patients are thus at higher risk for hemorrhagic transformation during procedures like EVT, where reperfusion of ischemic brain tissue places additional strain on fragile vessels. This fragility not only predisposes patients to further hemorrhagic events but also exacerbates ischemic injury during reperfusion, as the sudden restoration of blood flow to previously oxygen-starved tissues can generate oxidative stress and free radical damage, known as reperfusion injury.^20^ These complications further hinder the brain’s ability to recover from ischemic damage and complicate the treatment process.

Third, asymptomatic HI and SAH trigger secondary biological effects, such as inflammation and oxidative stress, that extend the damage beyond the original hemorrhagic site. When blood leaks into brain tissue, it activates an immune response, releasing inflammatory mediators such as cytokines and chemokines.^21^ These molecules can propagate damage by attacking both damaged and healthy tissues, creating a pro-inflammatory environment that impairs neuroplasticity, the brain’s ability to form new neural connections essential for recovery.^22^ Inflammation also encourages the formation of scar tissue (gliosis), which further hinders the brain’s ability to regenerate. Moreover, the breakdown of blood components, such as hemoglobin, releases iron, which catalyzes the production of reactive oxygen species.^23^ These free radicals cause oxidative stress, leading to lipid peroxidation, which damages cell membranes and further contributes to neuronal death.^24^ The combined effects of inflammation and oxidative stress create a toxic environment that slows down recovery and may lead to long-term functional impairments.

Moreover, asymptomatic HI and SAH complicates stroke management by making it difficult to balance anticoagulation therapy and blood pressure control. Physicians often take a more cautious approach to using anticoagulants, which can help prevent future strokes, but this can also increase the risk of ischemic events, negatively impacting recovery. Blood pressure management is also tricky—lowering it too much could worsen the hemorrhage, but not lowering it enough could reduce blood flow to the brain, hindering recovery.^25^ Additionally, the psychological stress of imaging-confirmed hemorrhage, even if asymptomatic, may slow recovery.^26^ These combined factors—microstructural damage, vascular issues, biological responses, and psychological stress—explain the worse outcomes seen in patients with asymptomatic HI and SAH, emphasizing the need for careful monitoring and possibly revising treatment approaches.

### Limitations

This study has several important limitations. First, although we applied advanced statistical methods such as PSM and IPTW to adjust for confounders, the retrospective nature of this analysis introduces potential bias. Despite these adjustments, the possibility of unmeasured or residual confounding cannot be entirely excluded. Second, the study was conducted across tertiary hospitals in China, which may limit the generalizability of our findings to other populations and health care settings, particularly in regions with different stroke management protocols. Additionally, the sample size, although adequate for detecting significant associations, may limit the power of certain subgroup analyses, and further studies with larger cohorts are needed to confirm our findings.

## Conclusions

In this secondary analysis of a randomized clinical trial, asymptomatic HI and SAH were associated with significantly worse long-term outcomes in patients undergoing mechanical thrombectomy for acute ischemic stroke. Although traditionally considered low risk, these hemorrhages were shown to negatively affect recovery.

## Notes

Supplement 1.
Trial Protocol

Supplement 2. **eFigure 1.** Forest Plot for Stratified Analysis of Small-Volume aICH and mRS Score of 0 or 1 at 90 Days by Subgroup **eFigure 2.** Forest Plot for Stratified Analysis of Small-Volume aICH and mRS Score of 0 to 2 at 90 Days by Subgroup

Supplement 3.
Data Sharing Statement
