# Error in Title

**DOI:** 10.1001/jamanetworkopen.2025.13651

**Published:** 2025-04-28

In the Original Investigation titled “Asymptomatic Hemorrhagic Events and Functional Outcomes in Acute Stroke: A Secondary Analysis of the DIRECT-MI Randomized Clinical Trial,”^1^ published March 28, 2025, there was an error in the title. DIRECT-MI should have been DIRECT-MT. This article has been corrected.^1^
