# Supplementary material for: Asymptomatic Hemorrhagic Events and Functional Outcomes in Acute Stroke: A Secondary Analysis of the DIRECT-MT Randomized Clinical Trial
Source: JAMA Netw Open. 2025 Mar 28;8(3):e252411. doi: 10.1001/jamanetworkopen.2025.2411 (PMC11953756; doi:10.1001/jamanetworkopen.2025.2411)
Supplement: Supplement 1. — Trial Protocol [file jamanetwopen-e252411-s001.pdf]

Protocol

This trial protocol has been provided by the authors to give readers additional information about their work.

Protocol for: Yang P, Zhang Y, Zhang L, et al. Endovascular thrombectomy with or without intravenous alteplase in acute stroke. N Engl J Med 2020;382:1981-93. DOI: 10.1056/NEJMoa2001123

This supplement contains the following items:

1. Original protocol in English (page 1 to 49), final protocol in English (page 50 to 97), summary of amendments in English (page 98 to 109)
2. Original statistical analysis plan in English (page 110 to 148), final statistical analysis plan in English (page 149 to 189), summary of amendments in English (page 151)

Direct Intra-arterial thrombectomy in order to Revascularize AIS patients with large vessel occlusion Efficiently in Chinese Tertiary hospitals: a Multicenter randomized clinical Trial (DIRECT-MT)

Project leaders:

Liu Jianmin,Deng Benqiang

Charles Majoie, Yvo Roos

Sponsor: Changhai Hospital Affiliated to the Second Military Medical University

CRO: Cardiovascular Chinese Research Center

Protocol No.: CH01

Version No.: V1.0

Date: December 1, 2017

Statement of Confidentiality

The information contained in this document involves trade secrets and business information, which is confidential or proprietary and shall not be disclosed by any person unless required by regulations. Any individual who has access to the document shall be aware that the document is proprietary or confidential and shall not disclose the document without authorization. The above restrictions on the confidentiality of the document will apply to any proprietary or confidential data provided to you in the future.

Protocol title:

Direct Intra-arterial thrombectomy in order to Revascularize AIS patients with large vessel occlusion Efficiently in Chinese Tertiary hospitals: a Multicenter randomized clinical Trial (DIRECT-MT)

Protocol ID	CH01
Version	1.0
Date	December 1, 2017
Project leaders	Prof Liu Jianmin, Changhai Hospital Affiliated to the Second Military Medical University<br><br>Prof Deng Benqiang, Changhai Hospital Affiliated to the Second Military Medical University<br><br>Prof dr Charles Majoie, Academisch Medisch Centrum bij de Universitieit van Amsterdam (AMC)<br><br>Prof dr Yvo Roos, Academisch Medisch Centrum bij de Universitieit van Amsterdam (AMC)
Coordinating investigators	Prof Yang Pengfei, Changhai Hospital Affiliated to the Second Military Medical University<br><br>Prof Zhang Yongwei, Changhai Hospital Affiliated to the Second Military Medical University
Sponsor	Changhai Hospital Affiliated to the Second Military Medical University
Subsidizing parties	Stroke Prevention and Control Engineering Commission of National Health and Family Planning Commission of the People's Republic of China, Wu Jieping Medical Foundation
CRO	Cardiovascular Chinese Research Center

Protocol Signature Sheet

Name	Signature	Date
Sponsor:<br><br>Changhai Hospital Affiliated to the Second Military Medical University		

I have read this trial protocol carefully and recognize that this protocol covers all the necessary contents for the implementation of the trial. I will conduct the study according to the protocol and complete the study within the specified period of time.

I will provide copies of this study protocol and all relevant information to all staff who assist me in conducting this study. I will discuss these materials with them to ensure that they fully understand the test drug and how to conduct the trial.

Name	Signature	Date
[Principal investigators]:		

Contents

List of abbreviations and definitions of terms **7**
SUMMARY **9**
1. Introduction and rationale **11**
2. Study objectives **12**
3. Study design **12**
4. Study population **12**
4.1. Population (Base) 12
4.2. Participating centers and center eligibility 13
4.3. Inclusion criteria 13
4.4. Exclusion criteria 14
4.5. Sample size calculation 14
5. Treatment of subjects **15**
5.1. Investigational treatment 15
5.2. Use of co-intervention 15
5.3. Escape medication 15
6. Investigational product **15**
6.1. Name and description of investigational product 15
6.2. Summary of findings from clinical studies 16
6.3. Summary of known and potential risks and benefits 16
6.4. Description and justification of route of administration and dosage 16
7. Non-investigational product **16**
7.1. Name and description of non-investigational products 16
7.2. Summary of findings from clinical studies 16
7.3. Summary of known and potential risks and benefits 17
8. Method **17**
8.1. Study outcomes 17
8.1.1. Main study outcome 17
8.1.2. Secondary outcomes 17
8.1.3. Safety outcomes 18
8.1.4. Other study parameters 18
8.2. Randomization, blinding and treatment allocation 18
8.3. Study procedures 19
8.4. Withdrawal of individual subjects 19
8.5. Premature termination of the study 19
9. Safety reporting **19**
9.1. Temporary halt for reasons of subject safety 19

9.2. AEs, SAEs and SUSARs 19
9.2.1. Adverse events (AEs) 19
9.2.2. Serious adverse events (SAEs) 20
9.3. Follow-up of adverse events 20
9.4. Data Safety Monitoring Board (DSMB) 20
10. Statistical analysis 21
10.1. Statistical analysis 21
10.2. Subgroup analysis 21
10.3. Interim analysis 22
11. Ethical considerations 22
11.1. Regulation statement 22
11.2. Recruitment and consent 22
11.3. Problems of minors or incapacitated subjects 22
11.4. Benefits and risks assessment, group relatedness 23
11.5. Compensation for injury 23
12. Administrative aspects, monitoring and publication 23
12.1. Handling and storage of data and documents 23
12.2. Monitoring and quality assurance 23
12.3. Amendment 23
12.4. Annual progress report 23
12.5. Temporary halt and (prematurely) end of study report 24
12.6. Public disclosure and publication policy 24
13. References 24
14. Table 29
Table 1 Modified Rankin Scale [35] 29
Table 2 Extended Treatment In Cerebral Ischemia (Etici) Scale [36] 30
Table 3 NIH Stroke Scale 31
Table 4 Barthel Index [40] 35
Table 5 EUROQOL 5D-5L [39] 37
Table 6 Clot Burden Score for CTA and MRA [46] 39
Table 7 Collateral Score[43] 39
Table 8 Classification pf Infarct in a New Territory [42] 40
Table 9 Report of Suspicious Medical Device Adverse Events 41
15. Figure 43
Figure 1 DIRECT-MT Trial Logo 43
Figure 2 Patient Flow in the Trial 43
16. Appendix 44

16.1 Study committees 44
Steering Committee 44
Data Safety Monitoring Board 44
Imaging Assessment Committee 44
Adverse Event Adjudication Committee 44
16.2 DIRECT-MT recommendations of the Steering Committee with regard to type of mechanical thrombectomy and use of thrombolytic agents during endovascular procedures. 44
General 44
Neuroimaging 45
Additional thrombolytic agents, dose and type 45
Type of mechanical thrombectomy device(s) 45
16.3 Imaging requirements 45
16.3.1 Minimum baseline imaging requirements 45
When 46
How 46
16.3.2 Intervention-related angiographic imaging 46
When 46
How 47
16.3.3 Minimum follow-up imaging requirements 48
When 48
How 48

List of abbreviations and definitions of terms

AE	Adverse event
AIS	Acute ischemic stroke
AR	Adverse Reaction
ASA	Acetyl salicylic acid
CT	Computed tomography
CTA	Computed tomography angiography
CV	Curriculum Vitae
DSMB	Data Safety Monitoring Board
EC	Ethics committee
EU	European Union
GCP	Good Clinical Practice
IAT	Intra-arterial treatment
IB	Investigator's Brochure
ICF	Informed Consent Form
ICH	Intracerebral hemorrhage
IMP	Investigational Medicinal Product
IU	International standard unit
IV	Intravenous
MRI	Magnetic resonance imaging
NIHSS	NIH Stroke Scale test
(S) AE	(Serious) adverse event
sICH	Symptomatic intracerebral hemorrhage
Sponsor	The sponsor is the party that commissions the organization or performance of the research, for example a pharmaceutical company, academic hospital, scientific organization or investigator. A party that provides funding for a study

	but does not commission is not regarded as the sponsor, but referred to as a subsidizing party.
SUSAR	Suspected unexpected serious adverse reaction
tPA	Tissue plasminogen activator

SUMMARY

Protocol title: Direct Intra-arterial thrombectomy in order to Revascularize AIS patients with large vessel occlusion Efficiently in Chinese Tertiary hospitals: a Multicenter randomized clinical Trial (DIRECT-MT)

Rationale: Intra-arterial treatment (IAT) by means of retrievable stents has been proven safe and effective in patients with acute ischemic stroke with confirmed large vessel occlusion of the anterior circulation and in whom the procedure can be started within 6 hours from onset. Despite recanalization, a considerable proportion of patients do not recover. This can be attributed to potential adverse effects of the intravenous treatment (IVT) prior to IAT. These effects could include neurotoxicity, blood brain barrier leakage and thrombus fragmentation through softening of the thrombus.

Another reason for non-recovery in MRCLEAN was the occurrence of symptomatic intracranial hemorrhage (sICH) in 7% of patients, which was fatal in 65%. sICH occurred as often in the intervention as in the control group, suggesting that this complication could not be attributed to the IAT, but rather to pre-treatment with IVT. Therefore, we hypothesize that direct IAT may lead to an 8% absolute increase in good outcome compared to IAT preceded by IVT.

Objective: To assess the effect of direct IAT compared to IVT followed by IAT, in patients with acute ischemic stroke, caused by a CTA-confirmed occlusion of the anterior circulation (ICA-T/L, M1, proximal M2) on functional outcome.

Study design: This is a parallel group, randomized clinical trial of direct IAT versus IVT with IAT. The trial has observer blind assessment of the primary outcome and of neuro-imaging at baseline and follow up.

Study population: Patients with acute ischemic stroke and a confirmed anterior circulation occlusion by CTA. Initiation of IVT must be feasible within 4.5 hours from symptom onset. Age must be 18 or over and NIHSS 2 or more.

INCLUSION CRITERIA

- a clinical diagnosis of acute ischemic stroke,
- caused by a large vessel occlusion of the anterior circulation (distal intracranial carotid artery or middle M1/proximal M2) cerebral artery confirmed by CTA,
- CT or MRI ruling out intracranial hemorrhage,
- eligible for IVT and IAT (within 4.5 hours after symptom onset),
- a score of at least 2 on the NIH Stroke Scale,
- age of 18 years or older,
- written informed consent.

EXCLUSION CRITERIA

- Pre-stroke disability which interferes with the assessment of functional outcome at 90 days, i.e. mRS >2
- Any contra-indication for IVT, according to guidelines of the American Heart Association, i.e.:
    - arterial blood pressure exceeding 185/110 mmHg
    - blood glucose less than 2.7 or over 22.2 mmol/L
    - cerebral infarction in the previous 6 weeks with residual neurological deficit or signs of recent infarction on neuro-imaging
    - serious head trauma in the previous 3 months
    - major surgery or serious trauma in the previous 2 weeks
    - gastrointestinal or urinary tract hemorrhage in the previous 3 weeks
    - previous intracerebral hemorrhage
    - use of anticoagulant with INR exceeding 1.7
    - known thrombocyte count less than 100 x 10^9/L
    - treatment with direct thrombin or factor X inhibitors
    - treatment with heparin (APTT exceeds the upper limit of normal value) in the previous 48 hours.

Intervention: The intervention group will undergo immediate IAT using a stent retriever, as recommended by the steering committee. The standard care group will receive IVT 0.9 mg/kg with a maximum dose of 90 mg in one hour, followed by IAT using a stent retriever. We strive to reduce delays associated with IVT administration to a minimum to adequately assess the effect of IVT itself with IAT.

Main study parameters/outcomes: The primary effect parameter will be the common odds ratio, estimated with ordinal logistic regression, which represents the shift on the full distribution of the modified Rankin Scale at 3 months. The estimate will be adjusted for the known prognostic variables age, pre-stroke mRS, time from onset to randomization, stroke severity (NIHSS) and collaterals and adjusted and unadjusted estimates with corresponding 95% confidence intervals will be reported.

Secondary outcomes include mortality at 90 days, stroke severity at 24-72 hours and 5-7 days, recanalization on CTA at 24-72 hours, dichotomous clinical outcome on the mRS and infarct size at 5-7 days. Safety outcomes include rate of sICH.

1. Introduction and rationale

Stroke is a major cause of death and disability. The latest National Epidemiological Survey of Stroke in China [1] (Ness-China) showed that: the standardized prevalence, incidence and mortality of stroke in China in 2013 were 1114.8/100 thousand person/year, 246.8/100 thousand person/year and 114.8/100 thousand person/year respectively. In 1985, the prevalence of stroke in China was only 365/100 thousand person/year [2]. In the case of a gradual decline in the incidence and mortality of stroke in European and American countries, the incidence of Chinese people gradually increased at a rate of 8.7% per year, which was significantly higher than the overall annual incidence of stroke in the world [3-5].

Early 2015, the outlook of acute stroke changed dramatically over the course of a few months. It was shown that patients with acute ischemic stroke (AIS) caused by a large vessel occlusion of the anterior circulation benefit from intra-arterial treatment (IAT). IAT using a stent retriever leads to an absolute increase in good functional outcome in 15% to 25% of patients treated within 6 hours. This was first reported in the MR CLEAN trial and later confirmed in 4 other trials [6-10].

In randomized trials of acute ischemic stroke, intravenous thrombolysis (IVT) with alteplase strongly reduced the risk of a poor outcome [11, 12]. However, two thirds of the patients treated with IVT within 3 hours of stroke onset in these trials were dead or dependent at the end of follow-up. In the MR CLEAN trial, 67% of the patients in the endovascular treatment group were dead or dependent at three months. The high risk of a poor outcome, even after these acute revascularization strategies, may to a large extent be explained by no-reflow. No-reflow has been linked to distal micro vascular damage or dysfunction as a result of tissue necrosis and cell death, or the intervention simply being late.

Currently the role of IVT in acute ischemic stroke treatment with IAT is unclear. The incidence of bleeding complications was similar in MR CLEAN to the frequency in the NINDS IVT trial and SITS MOST registry [13, 14]. In MR CLEAN, the occurrence of symptomatic intracranial hemorrhage (sICH) (7%, fatal in 65%) was similar between the intervention and the control group, suggesting that this complication could not be attributed to the IAT, but rather to pre-treatment with IVT. In 2017, a retrospective ACTUAL study based on Chinese population showed that the incidence of sICH in direct endovascular treatment group and bridging treatment group was higher than that in RCT study of previous IAT (13.8% and 13.0%) [15-20]; at the same time, the incidence of aICH in the intravascular treatment group was significantly lower than that in the bridging treatment group (28.3% vs. 44.9%, P=0.01). This may be related to the distribution characteristics of the cause of stroke in Chinese population. In the Asian population ischemic stroke reported, the proportion of intracranial atherosclerotic stenosis was as high as 30 - 50%, which was significantly higher than that of other populations [21-25]. The high incidence of intracranial atherosclerotic stenosis implied that the use proportion of intracranial stent implantation and GP2b3a receptor antagonist increased significantly. In ACTUAL study, the proportion of stent implantation in direct endovascular treatment group and bridging treatment group was 22.5% and 23.2% respectively, and the use proportion of GP2b3a receptor antagonists was 20.3% and 10.9% respectively. Whether atherosclerotic stenosis can affect the efficacy of IVT, and whether the increase in the proportion of stent implantation will

increase the incidence of ICH after IVT, which are currently unknown and need to be studied. The incidence of sICH between the two groups was similar, and whether suggesting that the occurrence of sICH could not be attributed to the IAT, but rather to pre-treatment with IVT. Further, IVT could have other potential deleterious effects such as neurotoxicity and loss of blood brain barrier integrity. (26) If IVT softens the thrombus prior to IAT, this could also lead to increased fragmentation rates, making successful reperfusion more difficult to achieve. Last, but not least, we know from EM scanning studies that fibrin forms around the struts of a stent retriever when in position. Systemic alteplase treatment may impair this fibrin formation and adversely affect the thrombectomy results[1].

We hypothesize that direct IAT, without pretreatment with IVT, in selected patients may lead to an 8% absolute increase in good outcome because of a reduction in the occurrence of sICH and an increase in treatment effect of IAT.

MR CLEAN is the earliest and only completed RCT study on the evaluation of the efficacy of IAT. This study intends to conduct in-depth cooperation with MR CLEAN study team in the Netherlands, and conducts an international prospective multi-center randomized controlled study in both locations to explore the differences in the clinical outcome between the two to answer the concept whether the clinical outcome of this direct IAT is better than that of the current treatment by comparing direct IAT with IVT and IAT bridging treatments, and the efficacy of stents in different populations (12).

2. Study objectives

The primary objective of this trial is to assess the effect of direct IAT compared with IVT followed by IAT, on functional outcome in patients with AIS, caused by an anterior circulation occlusion that is confirmed by neuro-imaging.

The secondary objective is to explore for superiority of direct IAT relative to IVT followed by IAT.

The tertiary objective is to assess the effect of direct IAT compared with IVT with IAT on neurological recovery (NIHSS), infarct size and occurrence of sICH.

The fourth objective is to collect thrombi and to analyze them with respect to their potential for treatment effect modification.

3. Study design

This is a multicenter phase IV prospective randomized clinical trial with open-label treatment and blinded outcome assessment (PROBE). The study will run for 4 years in intervention centers.

4. Study population

4.1. Population (Base)

[1] Personal communication:A. van Es, B.Emmer, B van der Kallen,G Lycklama and H van Beusekom 2016.Submitted to neurology

The latest National Epidemiological Survey of Stroke in China [(1)] (Ness-China) showed that: the standardized prevalence, incidence and mortality of stroke in China in 2013 were 1114.8/100 thousand person/year, 246.8/100 thousand person/year and 114.8/100 thousand person/year respectively. In 1985, the prevalence of stroke in China was only 365/100 thousand person/year [(2)]. In the case of a gradual decline in the incidence and mortality of stroke in European and American countries, the incidence of Chinese people gradually increased at a rate of 8.7% per year, which was significantly higher than the overall annual incidence of stroke in the world [(3-5)].

4.2. Participating centers and center eligibility

To be fully eligible for participation in the trial and to include patients in the trial, centers should meet the following minimum criteria:

- Local tertiary hospitals;
- Centers with experience in conducting acute stroke trials;
- It can simultaneously perform intravenous thrombolysis and endovascular thrombectomy, and completes more than 30 endovascular treatment of acute ischemic stroke each year,
- DNT <60min; DTP <90min
- The intervention team should have experience with endovascular interventions for cerebrovascular disease (IAT, carotid stenting or aneurysm coiling), peripheral artery disease, or coronary artery disease, and the stroke team (which includes neurologists and interventionists) should have previous experience with intra-arterial treatment,
- The intervention team should make use of one or more of the devices that have been approved by CFDA. Use of other devices is not allowed in the trial.
- At least one member of the intervention team should have previous experience with the particular device.

Note: Patients may only be included in the trial when the intervention team that will actually treat the patient includes at least one interventionist with previous experience with IAT.

4.3. Inclusion criteria

In order to be eligible to participate in this study, a subject must meet all of the following criteria:

- a clinical diagnosis of acute ischemic stroke;
- caused by a large vessel occlusion of the anterior circulation (distal intracranial carotid artery or middle M1/proximal M2) cerebral artery confirmed by CTA;
- CT or MRI ruling out intracranial hemorrhage;
- eligible for IVT and IAT (within 4.5 hours after symptom onset);
- NIHSS ≥ 2;

- age of 18 years or older;
- written informed consent.

4.4. Exclusion criteria

A potential subject who meets any of the following criteria will be excluded from participation in this study:

- Pre-stroke disability which interferes with the assessment of functional outcome at 90 days, i.e. mRS >2;
- Any contra-indication for IVT, according to guidelines of the American Heart Association [27], i.e.:
  - blood pressure > 185/110 mmHg,
  - blood glucose < 2.7 or > 22.2 mmol/L,
  - cerebral infarction in the previous 6 weeks with residual neurological deficit or signs of recent infarction on neuro-imaging,
  - serious head trauma in the previous 3 months,
  - major surgery or serious trauma in the previous 2 weeks,
  - gastrointestinal or urinary tract hemorrhage in the previous 3 weeks,
  - previous intracerebral hemorrhage,
  - use of anticoagulant with INR exceeding 1.7,
  - known thrombocyte count less than 100 × 10^9/L
  - treatment with direct thrombin or factor X inhibitors,
  - treatment with heparin (APTT exceeds the upper limit of normal value) in the previous 48 hours.

4.5. Sample size calculation

We based our estimations on the distribution of the modified Rankin Scale (mrS) in the control group of the trial, which we derived from the intervention group of the MR CLEAN trial [9]: mRS 0: 3%; mRS 1: 9%; mRS 2: 21%; mRS 3: 18%; mRS 4: 22%; mRS 5: 6% and mRS 6: 21%. We assumed a favorable treatment effect with a common odds ratio (cOR) of 1.54, which corresponds to an absolute risk difference of having a score on the modified Rankin Scale of 0-2 of approximately 8%. The main purpose is to demonstrate non-inferiority, that is, the lower limit of the 95% confidence interval does not cross the pre-specified cOR non-inferiority Cutoff of 0.8. In a simulation with 5000 runs we computed the proportion of positive trials, for a given sample size. This yielded a sample size of 680, providing 99% power to detect a true treatment effect, with two-sided alpha =0.05. In the analysis we will use covariate adjustment, which reduces the required

sample size with 25% [28, 29]. Therefore, the aim is to include 540 patients, 270 in each group of the trial, considering a dropout rate of 5%.

5. Treatment of subjects

5.1. Investigational treatment

Patients in the control group will receive IVT (alteplase) according to the guidelines of the American Heart Association. [27] Patients in the intervention group will not receive this treatment (nor placebo) and proceed directly with IAT. Patients in both groups will undergo IAT. Please note that to assess the effect of IVT itself and not the applied treatment strategy, we strive to reduce delays in the control group due to IVT administration to an absolute minimum. Remaining differences between treatment groups in time from randomization to groin puncture will be recorded. All stent retriever devices for IAT, which are approved by CFDA for this purpose, are allowed in the trial as a first line of defense.

Other mechanical devices (aspiration devices) are allowed as a second option, when the first device has failed according to the interventionist, usually after 3 passes. The further choice of the particular device for a certain patient is left to the discretion of the interventionist.

The target time from study noncontrast CT to groin puncture will be as fast as possible. All patients must undergo groin puncture within a median of 60 minutes after non-contrast CT acquisition.

5.2. Use of co-intervention

No standard co-medication is advised by the steering committee. Antiplatelet or antithrombotic treatment will generally be started at 24 hours after the intervention, according to national protocols.

5.3. Escape medication

If deemed by the interventionist, local application (intra-arterial) of alteplase is allowed in any of the patients included in the DIRECT-MT. Patients in the direct IAT group in whom good recanalization (eTICI 2b-3) was not reached, may be treated afterwards with 0.9 mg/kg IVT if the 4.5 hour window or maximum dose is not exceeded. Patients who have been pre-treated with i.v. alteplase should not receive more than 30mg alteplase during intra-arterial treatment. The steering committee recommends that the alteplase is delivered in shots of 5 mg in 5-10 minute intervals.

In individual cases, an equivalent dose of 400,000 U urokinase, delivered in shots of 50.000 - 100.000 U, in 5-10 minutes time intervals, is also accepted as escape medication.

Vessel patency should be checked after each shot.

6. Investigational product

6.1. Name and description of investigational product

The comparator in this trial is IVT with alteplase (actilyse). The intervention is omitting IVT before

IAT.

6.2. Summary of findings from clinical studies

The value of IVT in patients with AIS has been determined in multiple RCTs with a potential treatment window up to 4.5 hours after symptom onset [30, 31]. It has been a standard care for several years. All trials investigating the benefit of IAT in AIS had a control group consisting of patients receiving usual care [6-9, 32]. This meant that few patients were treated directly, without prior IVT. In MR CLEAN, this concerned only 55 patients (11%). Subgroup analysis showed a similar effect size in patients not treated with IVT (OR = 2.06 [95% Confidence Interval (CI): 0.69-6.13]) as in patients pretreated with IVT (OR =1.71 [95% CI: 1.22-2.40]). [9] REVASCAT showed comparable results: 56 patients not treated with IVT (OR = 2.6 [95% CI: 1.0-7.1]) as to 76 patients who were pretreated (OR = 1.4 [95% CI: 0.8-2.6]). [7] Moreover, in ESCAPE, patients without IV pretreatment seemed to benefit (OR = 2.6 [95% CI: 1.1-5.9]) from endovascular treatment. [6] When we combined the published data there is no heterogeneity (p= 0.78). In a fixed effect model, the effect estimate is quite precise and statistically significant (OR = 2.3 [95% CI: 1.5-3.7]). We believe that the data from these three randomized controlled trials show that patients not pretreated with IVT may benefit from intervention.

6.3. Summary of known and potential risks and benefits

For known possible undesirable effects of actilyse, see the summary of product characteristics supplied.

6.4. Description and justification of route of administration and dosage

The route and dosage of administration are based on the American Heart Association guidelines.

7. Non-investigational product

7.1. Name and description of non-investigational products

Stent-retrievers for IAT are the background treatment in this trial. The devices listed below may be used as primary device for IAT.

Device name	Manufacturer	Description
**Solitaire**	Medtronic / Covidien	Retrievable stent
**Trevo stent**	Stryker	Retrievable stent
**Revive stent**	Codman/DePuy-Synthes	Retrievable stent

7.2. Summary of findings from clinical studies

Seven randomized clinical trials that predominantly used stent thrombectomy have been carried out. [6-10, 33, 34] All trials showed a beneficial effect of intervention compared to usual care, which most often included treatment with iv-alteplase. The effect size ranged from 11 to approximately 25% increase in proportion of non-disabled patients at 3 months after randomization. [6-10, 33, 34]The

treatment is already established as standard of care. [32] As stated in paragraph 7.3, the subgroup analyses of recent trials suggest that patients not pretreated with IVT may benefit from intervention.

7.3. Summary of known and potential risks and benefits

The potential benefits of the intervention have been described in 3.3. The potential risks consist of intracranial and extracranial hemorrhage and hemorrhagic infarction, procedure related risks such as dissection, perforation and infarctions in other vascular territories, and postprocedural events such as infections. In the 5 trials, the risks of hemorrhage and hemorrhagic infarction were equal for both the intervention group as the control group. Postprocedural events such as pneumonia and other infections occurred in similar frequencies in both groups, and procedure-related events were infrequent.

8. Method

8.1. Study outcomes

8.1.1. Main study outcome

The primary outcome is the score on the modified Rankin Scale (Table 1 in Appendix) at 90 days (± 14 days). [35] The mRS is the preferred disability parameter for clinical trials in stroke. The mRS is an ordinal hierarchical scale incorporating six categories from 0 up to and including 5, and describes the range of disability encountered post stroke. 'Death' is assigned a score of 6. Assessment of outcome on the mRS will be performed by independent assessors, blinded to the allocated and actually received treatment. Their assessment will be based on standardized reports of a telephone interview by trained research personnel who are not aware of treatment allocation.

8.1.2. Secondary outcomes

Secondary outcomes are the following:

- Death within 90 days (± 14 days)
- Pre-interventional recanalization
- eTICI score on final angiography of IAT. [36] (Table 2 in Appendix)
- Recanalization rate at 24-72 hours, assessed with CTA
- Score on the NIHSS at 24-72 hours and 5-7 days, or at discharge. [37] (Table 3 in Appendix)
- Final infarct volume at 5-7 days. Final infarct volume will be assessed with the use of an automated, validated algorithm. [38] Infarct size at day 5-7 will be compared with plain CT and perfusion CT results (if available) at baseline.
- Dichotomized mRS of 0-1 vs. 2-6 at 90 days (± 14 days)
- Dichotomized mRS of 0-2 vs. 3-6 at 90 days (± 14 days)

– Dichotomized mRS of 0-3 vs. 4-6 at 90 days (± 14 days)

– Score on the EQ5D-5L and Barthel index at 90 days (± 14 days) [39] [40]

8.1.3. Safety outcomes

– Hemorrhages according to the Heidelberg criteria [40]

– sICH scored according to the Heidelberg criteria [41]

– Embolization in new territory on angiography during IAT

– Occurrence of aneurysma spurium

– Occurrence of groin hematoma

– Infarction in new territory at 5-7 days [42] (Table 8 in Appendix)

– Death from all causes within 90 days (± 14 days)

8.1.4. Other study parameters

Baseline parameters that will be recorded include age; sex; previous stroke; conditions such as hypertension, diabetes mellitus, atrial fibrillation, myocardial infarction; smoking status; medication including antihypertensive treatment, antiplatelet agents and anticoagulants; vital parameters such as blood pressure, body temperature; weight and height; neurological examinations including NIHSS; laboratory examination including INR, APTT, C-reactive protein, glucose, creatinine; and imaging results on admission (e.g. clot burden score, table 6 in Appendix).

We will record the actually received dose, type and timing of iv thrombolytic medication.

Additionally, we will record time from onset to ER, CT, randomization, start of IAT, first reperfusion and end of procedure. The devices and the order in which they are used will be recorded, and the type of anesthesia (if any) and sedation will be noted.

Last, during the 90 day study period, information regarding the direct treatment cost will be collected.

8.2. Randomization, blinding and treatment allocation

The randomization procedure will be computer- and web-based. Randomization is allowed when the occlusion has been established by CTA. Randomization will be stratified by center.

It will not be possible to view the treatment allocation before the patient is registered in the study database, nor will it be possible to remove the patient from the study after treatment assignment has become known. Both patient and treating physician will be aware of the treatment assignment. Information on outcome at three months will be assessed through standardized forms and procedures, by a trained investigator blinded for treatment allocation. Interviews will be recorded. Assessors who are blinded to the treatment allocation will perform assessment of outcome on the

modified Rankin scale on this information. Results of neuro-imaging will be also assessed in a blinded manner. Information on treatment allocation will be kept separate from the main study database. The steering committee will be kept unaware of the results of interim analyses of efficacy and safety. An independent trial statistician will combine data on treatment allocation with the clinical data in order to report to the data monitoring committee (DSMB).

8.3. Study procedures

All patients will undergo assessment of the NIHSS at baseline, 24-72 hours and 5-7 days, which is routine in clinical procedure. It will be carried out by certified assessors. Patients will undergo NCCT and CTA at baseline. After 24-72 hours CTA is repeated to determine recanalization. At 5-7 days, patients will undergo NCCT to assess infarct size.

In addition, this trial also makes use of “waste material”: blood aspirated during intervention with retrieved thrombi during intervention. These thrombosis will be stored in the participating study centers for follow-up analysis.

8.4. Withdrawal of individual subjects

Subjects can leave the study at any time for any reason if they wish to do so without any consequences. The investigator can decide to withdraw a subject from the study for urgent medical reasons. The data from subjects who do not provide consent will be treated anonymously, and used for baseline analysis to further describe this population. At the time of analysis, missing data are interpolated, including the final mRS score. The key part of personal data will be cleared.

8.5. Premature termination of the study

The study will only be terminated prematurely if the Data Safety Monitoring Board recommends stopping. In case of premature termination of the study, the database will be closed after 90 days assessment of the last enrolled patient and results will be reported.

9. Safety reporting

9.1. Temporary halt for reasons of subject safety

The sponsor will suspend the study if there is sufficient ground that continuation of the study will jeopardize subject health or safety. The sponsor will notify the undue delay caused by temporary halt as well as the reason for such an action. The study will be suspended pending further review by the EC. The investigator should ensure that all subjects are kept informed.

9.2. AEs, SAEs and SUSARs

9.2.1. Adverse events (AEs)

Adverse events are defined as any undesirable experience occurring to a subject during the study, whether or not considered related to trial procedure. All adverse events reported spontaneously by

the subject or observed by the investigator or his staff will be recorded.

9.2.2. Serious adverse events (SAEs)

A serious adverse event is any unfavourable medical occurrence or effect as follows

- Results in death;
- Life threatening (at the time of the event);
- Require inpatient hospitalization or prolongation of existing inpatients' hospitalization.
- congenital anomaly or birth defect;
- results in persistent or significant disability or incapacity;
- that required medical or surgical intervention to preclude of;

Any other important medical event that did not result in any of the outcomes listed above due to medical or surgical intervention but could have been based upon appropriate medical judgment. An elective hospital admission will not be considered as a serious adverse event.

Serious adverse events will be immediately, after coming to notice of the investigator, reported to the trial coordinator, who is 24/7 available.

The investigator will report the following SAEs occurring in the study period to the sponsor without undue delay of obtaining knowledge of the events: Death from any cause; symptomatic intracranial hemorrhage scored, extracranial hemorrhage, aspiration pneumonia, allergic contrast reactions, new ischemic stroke in different vascular territory.

Technical complications or vascular damage at the target lesion such as perforation or dissection that do not lead to clinically detectable SAE and neurological deterioration not caused by intracranial hemorrhage, new ischemic stroke, but are considered as consistent with the natural course of the ischemic stroke and its treatment, will not be reported immediately.

Since all subjects are treated with IAT, SAEs of this study are reported using the "Suspicious Medical Device Adverse Event Report Form" (Table 9 in the appendix). The investigator should report to the sponsor and ethics committee within 24 hours of SAEs.

9.3. Follow-up of adverse events

All AEs will be followed until they have abated, or until a stable situation has been reached.

Depending on the event, follow up may require additional tests or medical procedures as indicated, and/or referral to the general physician or a medical specialist.

SAEs need to be reported till the end of the study in China, as defined in the protocol.

9.4. Data Safety Monitoring Board (DSMB)

In order to increase the safety of the intervention, the trial will be monitored by an independent DSMB. The DSMB will be chaired by a neurologist, and include a neuro-interventionist and an independent methodologist/statistician. The DSMB will meet frequently, at least annually or after inclusion of the next 100 patients (whichever comes first) and assess the occurrence of adverse events by center and by procedure. During the period of patient enrollment into the study, interim analyses of mortality and of any other information that is available on major outcomes (including serious adverse events believed to be due to treatment) will be supplied, in strict confidence, to the chairman of the DSMB, along with any other analyses that the DSMB may request. In the light of these analyses, DSMB will advise the chairman of the Steering Committee if, in their view, the randomized comparisons in DIRECT-MT have provided both (i) "proof beyond reasonable doubt" that for all, or for some specific types of patients, one particular treatment is clearly indicated or clearly contraindicated in terms of a net difference in outcome, and (ii) evidence that might reasonably be expected to materially influence patient management. Appropriate criteria of proof beyond reasonable doubt cannot be specified precisely, but a difference of at least 3 standard deviations in an interim analysis of a major outcome may be needed to justify halting, or modifying, the study prematurely. This criterion has the practical advantage that the number of interim analyses is of little importance.

The advice(s) of the DSMB will be sent to the sponsor of the study by the chair of the steering committee. Should the sponsor decide not to fully implement the advice of the DSMB, the sponsor will send the advice to the EC, including a note to substantiate why (part of) the advice of the DSMB will not be followed.

10. Statistical analysis

10.1. Statistical analysis

The primary effect parameter will be the common odds ratio, estimated with ordinal logistic regression, which represents the shift on the 7-category mRS scale measured at 3 months. The estimate will be adjusted for the known prognostic variables age, pre-stroke mRS, time from onset to randomization, stroke severity (NIHSS) and collaterals and adjusted and unadjusted estimates with corresponding 95% confidence intervals will be reported. To explore for non-inferiority, we will analyze whether the lower bound of the 95% CI crossed 0.8, our pre-specified non-inferiority margin.

If applicable, the secondary outcomes will be analyzed using linear, logistic, or ordered regression analysis method, with the same correction method as the primary outcomes.

All analyses will be performed according to the intention-to-treat principle. Baseline data by treatment allocation will be reported with statistical procedures. Missing values for baseline characteristics will be reported. Missing baseline characteristics will be imputed using regression imputation. Pre-defined subgroups will be analyzed by testing for interaction between the specific baseline characteristic and treatment.

10.2. Subgroup analysis

The effect of intervention on the modified Rankin Scale will be analyzed in subgroups determined by the following variables:

- Tertiles of time from onset of symptoms to randomization, groin puncture and revascularization
- Extracranial carotid obstruction
- Occlusion location
- Collateral grades 0 to 3 as defined by Tan et al. (43) (Table 7 in Appendix)
- Thrombus perviousness (44)

10.3. Interim analysis

See Paragraph 9.4.

11. Ethical considerations

11.1. Regulation statement

The study will be conducted according to the principles of the Declaration of Helsinki (October 2013) (45)

11.2. Recruitment and consent

Following Article 21 of "Standard for quality management of medical device clinical trials" (June 1, 2016)[2], the investigators should adequately explain the details of the clinical trial, including known, foreseeable risks and possible adverse event, etc., to the subject or to the guardians of subjects without capacity for civil conduct or with limited capacity for civil conduct. After full and detailed explanation, the subjects or their guardians sign the name and date in the informed consent form, and the investigators also need to sign the name and date in the informed consent form.

In view of half of the AIS patients have language impairment, lack of sense of disease, or other acute cognitive symptoms, following the first paragraph of Article 23 of "Standard for quality management of medical device clinical trials" (June 1, 2016), for incapacitated subjects, if the ethics committee agrees in principle, and investigators believe that subjects participating in clinical trials are in their own interest, they can also enter the clinical trial, but their guardians should sign the name and date before the trial;

11.3. Problems of minors or incapacitated subjects

Minors (under 18 years old) will not be included in this trial. In the trial, about 50% of patients have language defects due to stroke, and about a quarter of the patients may suffer from a certain degree of lack of sense of disease. In such case, we will inform the patient and the legal representative, and

[2] http://www.sda.gov.cn/WS01/CL1101/148101.html

seek the latter's written consent, as described in 11.2.

11.4. Benefits and risks assessment, group relatedness

The expected benefit from direct intra-arterial treatment compared to IVT followed by IAT may amount to 8% absolute increase in independent living at 3 months. Patients who have been allocated to the control group will be given usual treatment according to international, national and local guidelines. This includes treatment with IVT, followed by IAT.

11.5. Compensation for injury

Each participating center has purchased liability insurance. This insurance provides cover for damage to research subjects through injury or death caused by the study.

The insurance applies to the damage that becomes apparent during the study or within 4 years after the end of the study.

12. Administrative aspects, monitoring and publication

12.1. Handling and storage of data and documents

All data will be entered into a web-based database (OpenClinica) by local research personnel. Subject records are coded by a unique study number. The local investigators will keep a list showing codes and names. Unique documents with identifying information will be stored separately from the study database in digital files, categorized by study number on a secure drive system, only accessible to the study coordinator.

12.2. Monitoring and quality assurance

The monitors will arrange visits according to the speed of enrollment of each center and the deviations found in the past. In principle, the inspection visit will be arranged within 5 working days of the center enrollment. The monitor will validate informed consent and source data for all subjects. The monitoring data including but not limited to: in-patient medical records, outpatient medical records, follow-up medical records, imaging materials and evaluation forms, etc. At the same time, the monitor will check the integrity and consistency of OpenClinica data entry.

12.3. Amendment

Amendments are changes made to the research protocol after a favorable opinion by EC has been given. All amendments will be notified to the EC that gave a favorable opinion.

12.4. Annual progress report

The sponsor/investigator will submit a summary of the progress of the trial to the EC once a year. Information should be provided: the date of inclusion of the first subject, numbers of subjects included and numbers of subjects that have completed the trial, serious adverse events/ serious

adverse reactions, other problems and amendments.

12.5. Temporary halt and (prematurely) end of study report

The investigator/sponsor will notify the EC of the end of the study within a period of 8 weeks. The end of the study is defined as the last patient's last visit.

The sponsor will notify the EC immediately of a temporary halt of the study, including the reason of such an action.

In case the study is ended prematurely, the sponsor will notify the EC within 15 days, including the reasons for the premature termination.

Within one year after the end of the study, the investigator/sponsor will submit a final study report with the results of the study, including any publications/abstracts of the study, to the EC and the Competent Authority.

The insurance applies to the damage that becomes apparent during the study or within 4 years after the end of the study.

12.6. Public disclosure and publication policy

The trial will be registered in clinicaltrials.gov.

The study database will be closed within one month after the last scheduled follow-up date of the last included patient. A manuscript which at least describes the study and the answer to the primary research question will be submitted to a major clinical journal within 3 months from closure of the database. The manuscript will be shared with the financial sponsor(s) one month before submission, but the financial sponsor(s) will have no influence on its contents.

Anonymous data can be requested from the PI with a detailed description containing the aims and methods of the study for which the data are intended to be used. Data will be made available for this purpose at least 18 months after publication of the main report. Data may also be shared with non-commercial parties for scientific purposes, including individual patient meta-analyses, and with commercial parties for regulatory purposes.

These purposes should be specified in the informed consent form.

13. References

1. Wang W, Jiang B, Sun H, et al. Prevalence, Incidence, and Mortality of Stroke in China: Results from a Nationwide Population-Based Survey of 480 687 Adults. Circulation. 2017;135(8):759-71.

2. Wang C, Li SW, Wu SW, et al. Epidemiological survey of neurological disorders in six urban areas of the People's Republic of China. Zhong-Hua-Shen-Jing-Wai-Ke-Za-Zhi. 1985;1:2-7.

3. Feigin VL, Lawes CM, Bennett DA, et al. Worldwide stroke incidence and early case fatality reported in 56 population-based studies: a systematic review. Lancet Neurol. 2009;8(4):355-69.

4. Thrift AG, Thayabaranathan T, Howard G, et al. Global stroke statistics. Int J Stroke. 2017;12(1):13-32.

5. Kochanek KD, Murphy SL, Xu J, et al. Deaths: Final Data for 2014. Natl Vital Stat Rep. 2016;65(4):1-122.

6. Goyal M, Demchuk AM, Menon BK, Eesa M, Rempel JL, Thornton J, et al. Randomized assessment of rapid endovascular treatment of ischemic stroke. N Engl J Med. 2015;372(11):1019-30.

7. Jovin TG, Chamorro A, Cobo E, de Miquel MA, Molina CA, Rovira A, et al. Thrombectomy within 8 hours after symptom onset in ischemic stroke. N Engl J Med. 2015;372(24):2296-306.

8. Saver JL, Goyal M, Bonafe A, Diener HC, Levy EI,Pereira VM, et al. Stent-retriever thrombectomy after intravenous t-PA vs. t-PA alone in stroke. N Engl J Med. 2015;372(24):2285-95.

9. Brkhemer OA, Fransen PS, Beumer D, van den BergLA, Lingsma HF, Yoo AJ, et al. A randomized trial of intraarterial treatment for acute ischemic stroke. N Engl J Med. 2015;372(1):11-20.

10. Cmpbell BC, Mitchell PJ, Kleinig TJ, Dewey HM, Churilov L, Yassi N, et al.

Endovascular therapy for ischemic stroke with perfusion-imaging selection. N Engl J Med. 2015;372(11):1009-18.

11. Emberson J, Lees KR, Lyden P, Blackwell L, Albers G, Bluhmki E, et al. Effect of treatment delay, age, and stroke severity on the effects of intravenous thrombolysis with alteplase for acute ischaemic stroke: a meta-analysis of individual patient data from randomised trials. The Lancet. 2014;384(9958):1929-35.

12. Wardlaw JM, Murray V, Berge E, del Zoppo G, Sandercock P, Lindley RL, et al. Recombinant tissue plasminogen activator for acute ischaemic stroke: an updated systematic review and meta-analysis. Lancet. 2012;379(9834):2364-72.

13. Group TNt-PSS. Intracerebral Hemorrhage After Intravenous t-PA Therapy for Ischemic Stroke. Stroke. 1997;28(11):2109-18.

14. Mazya M, Egido JA, Ford GA, Lees KR, Mikulik R,Toni D, et al. Predicting the risk of symptomatic intracerebral hemorrhage in ischemic stroke treated with intravenous alteplase: safe Implementation of Treatments in Stroke (SITS) symptomatic intracerebral hemorrhage risk score. Stroke. 2012;43(6):1524-31.

15. ACTUAL Investigators. Direct endovascular treatment: an alternative for bridging therapy in anterior circulation large-vessel occlusion stroke. Eur J Neurol. 2017 Jul;24(7):935-943.

16. Berkhemer OA, Fransen PS, Beumer D, et al. A randomized trial of intraarterial treatment for acute ischemic stroke. N Engl J Med 2015; 372: 11–20.

17. Campbell BC, Mitchell PJ, Kleinig TJ, et al. Endovascular therapy for ischemic stroke with perfusion-imaging selection. N Engl J Med 2015; 372: 1009–1018.

18. Goyal M, Demchuk AM, Menon BK, et al. Randomized assessment of rapid endovascular treatment of ischemic stroke. N Engl J Med 2015; 372: 1019–1030.

19. Saver JL, Goyal M, Bonafe A, et al. Stent-retriever thrombectomy after intravenous t-PA vs. t-PA alone in stroke. N Engl J Med 2015; 372: 2285–2295.

20. Jovin TG, Chamorro A, Cobo E, et al. Thrombectomy within 8 hours after symptom onset in ischemic stroke. N Engl J Med 2015; 372: 2296–2306.

21. Arenillas JF. Intracranial atherosclerosis: current concepts. Stroke, 2011,42:S20-23.

22. Holmstedt CA, Turan TN, Chimowitz MI. Atherosclerotic intracranial arterial stenosis: risk factors, diagnosis, and treatment. Lancet Neurol, 2013,12:1106-1114.

23. Wityk RJ, Lehman D, Klag M, Coresh J, Ahn H, Litt B. Race and sex differences in the distribution of cerebral atherosclerosis. Stroke, 1996,27:1974-1980.

24. Sacco RL, Kargman DE, Gu Q, Zamanillo MC. Race-ethnicity and determinants of intracranial atherosclerotic cerebral infarction. The Northern Manhattan Stroke Study. Stroke, 1995,26:14-20.

25. Huang YN, Gao S, Huang Y, et al. Vascular lesion in Chinese patients with transient ischemic attacks. Neurology, 1997, 48(2):524-525.

26. Chandra RV, Leslie-Mazwi TM, Mehta BP, Derdeyn CP, Demchuk AM, Menon BK, et al. Does the use of IV tPA in the current era of rapid and predictable recanalization by mechanical embolectomy represent good value? Journal of NeuroInterventional Surgery. 2016;8(5):443-6.

27. Powers WJ, Derdeyn CP, Biller J, Coffey CS, HohBL, Jauch EC, et al. 2015 American Heart Association/American Stroke Association Focused Update of the 2013 Guidelines for the Early Management of Patients With Acute Ischemic Stroke Regarding Endovascular Treatment: A Guideline for Healthcare Professionals From the American Heart Association/American Stroke Association. Stroke. 2015;46(10):3020-35.

28. Hernandez AV, Steyerberg EW, Butcher I, Mushkudiani N, Taylor GS, Murray GD, et al. Adjustment for strong predictors of outcome in traumatic brain injury trials: 25% reduction in sample size requirements in the IMPACT study. J Neurotrauma. 2006;23(9):1295-303.

29. Lingsma H, Roozenbeek B, Steyerberg E, investigators I. Covariate adjustment increases statistical power in randomized controlled trials. J Clin Epidemiol. 2010;63(12):1391; author reply 2-3.

30. Wahlgren N, Ahmed N, Davalos A, Ford GA, Grond M, Hacke W, et al. Thrombolysis with alteplase for acute ischaemic stroke in the Safe Implementation of Thrombolysis in Stroke-Monitoring Study (SITS-MOST): an observational study. Lancet. 2007;369(9558):275-82.

31. Hacke W, Kaste M, Bluhmki E, Brozman M, DávalosA, Guidetti D, et al. Thrombolysis with Alteplase 3 to 4.5 Hours after Acute Ischemic Stroke. The New England Journal of Medicine. 2008;359(13):1317-29.

32. Campbell BC, Donnan GA, Lees KR, Hacke W, Khatri P, Hill MD, et al. Endovascular stent thrombectomy: the new standard of care for large vessel ischaemic stroke. Lancet Neurol. 2015;14(8):846-54.

33. Bracard S, Ducrocq X, Mas JL, Soudant M, Oppenheim C, Moulin T, et al. Mechanical thrombectomy after intravenous alteplaseversus alteplase alone after stroke (THRACE): a randomised controlled trial. Lancet Neurol. 2016;15(11):1138-47.

34. Muir KW, Ford GA, Messow CM, Ford I, Murray A, Clifton A, et al. Endovascular therapy for acute ischaemic stroke: the Pragmatic Ischaemic Stroke Thrombectomy Evaluation (PISTE) randomised, controlled trial. J Neurol Neurosurg Psychiatry.

2017;88(1):38-44.

35. van Swieten JC, Koudstaal PJ, Visser MC, Schouten HJ, van Gijn J. Interobserver agreement for the assessment of handicap in stroke patients. Stroke. 1988;19(5):604-7.

36. Goyal M, Fargen KM, Turk AS, Mocco J, Liebeskind DS, Frei D, et al. 2C or not 2C: defining an improved revascularization grading scale and the need for standardization of angiography outcomes in stroke trials. J Neurointerv Surg. 2014;6(2):83-6.

37. Brott T, Adams HP, Olinger CP, Marler JR, Barsan WG, Spilker J, et al. Measurements of acute cerebral ifarction: a clinical examination scale Stroke. 1989;20:864-70.

38. Boers AM, Marquering HA, Jochem JJ, Besselink NJ, Berkhemer OA, van der Lugt A, et al. Automated cerebral infarct volume measurement in follow-up noncontrast CT scans of patients with acute ischemic stroke. AJNR Am J Neuroradiol. 2013;34(8):1522-7.

39. Group E. EuroQol--a new facility for the measurement of health-related quality of life. Health Policy. 1990;16(3):199-208.

40. Mahoney FI, Barthel DW. Functional Evaluation: The Barthel Index. Md State Med J. 1965;14:61-5.

41. von Kummer R, Broderick JP, Campbell BC, Demchuk A, Goyal M, Hill MD, et al. The Heidelberg Bleeding Classification: Classification of Bleeding Events After Ischemic Stroke and Reperfusion Therapy. Stroke. 2015;46(10):2981-6.

42. Goyal M, Menon BK, Demchuk A, Saver JL, Eesa M,Majoie C, et al. Proposed methodology and classification of Infarct in New Territory (INT) after endovascular stroke treatment. J Neurointerv Surg. 2017;9(5):449-50.

43. Tan IY, Demchuk AM, Hopyan J, Zhang L, Gladstone D, Wong K, et al. CT angiography clot burden score and collateral score:correlation with clinical and radiologic outcomes in acute middle cerebral artery infarct. AJNR Am J Neuroradiol. 2009;30(3):525-31.

44. Santos EM, Marquering HA, den Blanken MD, Berkhemer OA, Boers AM, Yoo AJ, et al. Thrombus Permeability Is Associated With Improved Functional Outcome and Recanalization in Patients With Ischemic Stroke. Stroke. 2016;47(3):732-41.

45. World Medical A. World Medical Association Declaration of Helsinki: ethical principles for medical research involving human subjects. JAMA. 2013;310(20):2191-4.

46. Puetz V, Dzialowski I, Hill MD, Subramaniam S, Sylaja PN, Krol A, et al. Intracranial thrombus extent predicts clinical outcome, final infarct size and hemorrhagic transformation in ischemic stroke: the clot burden score. Int J Stroke. 2008;3(4):230-6.

14. Table

Table 1 Modified Rankin Scale [35]

The modified Rankin Scale (mRS) is an ordinal hierarchical scale ranging from 0 to 5, with higher scores indicating more severe disability. A score of 6 has been added to signify death.

Category	Short description	Long description
0	No symptoms	No symptoms
1	Symptoms, no disability	Minor symptoms that do not interfere with lifestyle
2	Slight disability	Slight disability, symptoms that lead to some restriction in lifestyle, but do not interfere with the patient's capacity to look after himself.
3	Moderate disability	Moderate disability, symptoms that significantly restrict lifestyle and prevent totally independent existence
4	Moderately severe disability	Moderately severe disability, symptoms that clearly prevent independent existence though not needing constant attention
5	Severe disability	Severe disability, totally dependent patient requiring constant attention day and night.
6	Death	Death

Table 2 Extended Treatment In Cerebral Ischemia (Etici) Scale [36]

eTICI grade	Short description	Long description
**0**	No perfusion	No antegrade flow beyond the point of occlusion
**1**	Limited reperfusion	Antegrade reperfusion past the initial occlusion, but limited distal branch filling with little or slow distal reperfusion
**2a**	<50% reperfusion	Antegrade reperfusion of less than half of the occluded target artery previously ischemic territory (eg, in 1 major division of the MCA and its territory)
**2b**	≥50% and <90% reperfusion	Antegrade reperfusion of more than half of the previously occluded target artery ischemic territory (eg, in 2 major divisions of the MCA and its territories)
**2c**	≥90% reperfusion	Near complete antegrade reperfusion of the previously occluded target artery ischemic territory, except for slow flow or distal emboli in a few distal cortical vessels
**3**	100% reperfusion	Complete antegrade reperfusion of the previously occluded target artery ischemic territory, with absence of visualized occlusion in all distal branches

MCA: middle cerebral artery; eTICI; extended treatment in cerebral ischemia scale

Table 3 NIH Stroke Scale

The NIHSS is an ordinal hierarchical scale to evaluate the severity of stroke by assessing a patient's performance. [23] Scores range from 0 to 42, with higher scores indicating a more severe deficit. Administer stroke scale items in the order listed. Record performance in each category after each subscale exam. Do not go back and change scores. Follow directions provided for each exam technique. Scores should reflect what the patient does, not what the clinician thinks the patient can do. The clinician should record answers while administering the exam and work quickly. Except where indicated, the patient should not be coached (i.e. repeated requests to patient to make a special effort).

Instructions	Scale definition
**1a. Level of consciousness.** The investigator must choose a response if a full evaluation is prevented by such obstacles as an endotracheal tube, language barrier, orotracheal trauma/bandages. A 3 is scored only if the patient makes no movement (other than reflexive posturing) in response to noxious stimulation.	0 = **Alert;** keenly responsive.<br>1 = **Not alert;** but arousable by minor stimulation to obey, answer, or respond.<br>2 = **Not alert;** required repeated stimulation to attend, or is obtunded and requires strong or painful stimulation to make movements (not stereotyped).<br>3 = Responds only with reflex motor or autonomic effects or totally unresponsive, flaccid and areflexic.
**1b. LOC Questions:** The patient is asked the month and his/her age. The answer must be correct – there is not partial credit for being close. Phasic and stuporous patients who do not comprehend the questions will score 2. Patients unable to speak because of endotracheal intubation, orotracheal trauma, severe dysarthria from any cause, language barrier, or any other problem not secondary to aphasia are given a 1. It is important that only the initial answer be graded and that the examiners not "help" the patient with verbal or non-verbal clues.	0 = Answers both questions correctly.<br>1 = Answers one question correctly.<br>2 = Answers neither question correctly.
**1c. LOC Commands:** The patient is asked to open and close the eyes and then to grip and release the non-paretic hand. Substitute another one step command if the hand cannot be used. Credit is given if an unequivocal attempt is made but not completed due to weakness. If the patient does not respond to command, the task should be demonstrated to him or her (pantomime), and the result scored<br>(i.e. follows none, one or two commands). Patients with trauma, amputation, or other physical impediments should be given suitable one-step commands. Only the first attempt is scored.	0 = Performs both tasks correctly.<br>1 = Performs one task correctly.<br>2 = Performs neither task correctly.
**2. Best Gaze:** Only horizontal eye movements will be tested. Voluntary or reflexive	**0= Normal.**<br>1= **Partial gaze palsy;** gaze is abnormal in one or both

(oculocephalic) eye movements will be scored, but caloric testing is not done. If the patient has a conjugate deviation of the eyes that can be overcome by voluntary or reflexive activity, the score will be a 1. If a patient has an isolated peripheral nerve paresis (CN III, IV or VI), score a 1. Gaze is testable in all aphasic patients. Patients with ocular trauma, bandages, preexisting blindness, or other disorder of visual acuity or fields should be tested with reflexive movements, and a choice made by the investigator. Establishing eye contact and then moving about the patient from side to side will occasionally clarify the presence of a partial gaze palsy.	eyes, but forced deviation or total gaze paresis is not present.<br>2= **Forced deviation;** or total gaze paresis not overcome by the oculocephalic maneuver.
**3. Visual:** Visual fields (upper and lower quadrants) are tested by confrontation, using finger counting or visual threat, as appropriate. Patients may be encouraged, but if they look at the side of the moving finger appropriately, this can be scored as normal. If there is unilateral blindness or enucleation, visual fields in the remaining eye are scored. Score 1 only if a clear-cut asymmetry, including quadrantanopia, is found. If patient is blind from any cause, score 3.<br>Double simultaneous stimulation is performed in this case.<br>If there is extinction, the patient receives a 1, and the results are used to respond to item 11.	**0= No visual loss.**<br>**1= Partial hemianopia.**<br>**2= Complete hemianopia.**<br>3= **Bilateral hemianopia**<br>(blind including cortical blindness)
**4. Facial palsy:** Ask or use pantomime to encourage the patient to show teeth or raise eyebrows and close eyes. Score symmetry of grimace in response to noxious stimuli in the poorly response or non-comprehending patient. If facial trauma/bandages, orotracheal tube, tape or other physical barriers obscure the face, these should be removed to the extent possible.	**0 = Normal symmetrical movements.**<br>1= **Minor paralysis**<br>(flattened nasolabial fold, asymmetry on smiling)<br>2= **Partial paralysis** (total or near-total paralysis of lower face)<br>3= **Complete paralysis of one or both sides** (absence of facial movement in the upper and lower face).
**5. Motor arm:** The limb is placed in the appropriate position: extend the arms (palms down) 90 degrees (if sitting) or 45 degrees (if supine). Drift is scored if the arm falls before 10 seconds. The aphasic patient is encouraged using urgency in the voice and pantomime, but not noxious stimulation. Each limb is tested in turn, beginning with the non-paretic arm. Only in the case of amputation or joint fusion at the shoulder, the examiner should record the score as untestable (UN), and clearly write the explanation for this choice.	0= **No drift**; limb holds 90 (or 45) degrees for full 10 seconds.<br>1= **Drift**; limb holds 90 (or 45) degrees, but drifts down before full 10 seconds; does not hit bed or other support.<br>2= **Some effort against gravity;** limb cannot get to or maintain (if cued) 90 (or 45) degrees, drifts down to bed, but has some effort against gravity.<br>3= **No effort against gravity;** limb falls.<br>**4= No movement.**<br>UN = Amputation or joint fusion: explain:<br>**5a = Left Arm.**<br>**5b = Right arm.**

**6. Motor leg:** The limb is placed in the appropriate position: hold the leg at 30 degrees (always tested supine). Drift is scored if the leg falls before 5 seconds. The aphasic patient is encouraged using urgency in the voice and pantomime, but not noxious stimulation. Each limb is tested in turn, beginning with the non-paretic leg. Only in the case of amputation or joint fusion at the hip, the examiner should record the score as untestable (UN), and clearly write the explanation for this choice.	0= **No drift;** leg holds 30-degree position for full 5 seconds.<br>1= **Drift;** leg falls by the end of the 5-second period but does not hit bed.<br>2= **Some effort against gravity;** leg falls to bed by 5 seconds, but has some effort against gravity.<br>3= **No effort against gravity;** leg falls to bed immediately.<br>**4= No movement.**<br>UN = Amputation or joint fusion: explain:<br>**6a. Left Leg**<br>**6b. Right Leg.**
**7. Limb ataxia:** This item is aimed at finding evidence of a unilateral cerebellar lesion. Test with eyes open. In case of visual defect, ensure testing is done in intact visual field. The finger-nose-finger and heel-shin tests are performed on both sides, and ataxia is scored only if present out of proportion to weakness. Ataxia is absent in the patient who cannot understand or is paralyzed. Only in the case of amputation or joint fusion, the examiner should record the score as untestable (UN), and clearly write the explanation for this choice. In case of blindness, test by having the patient touch nose from extended arm position.	0= Absent.<br>1**= Present in one limb.**<br>2**= Present in two limbs.**<br>UN = Amputation or joint fusion: explain:
**8. Sensory:** Sensation or grimace to pinprick when tested, or withdrawal from noxious stimulus in the obtunded or aphasic patient. Only sensory loss attributed to stroke is scored as abnormal and the examiner should test as many body areas (arms [not hands], legs, trunk, face) as needed to accurately check for hemisensory loss. A score of 2, 'severe or total sensory loss', should only be given when a severe or total loss of sensation can be clearly demonstrated. Stuporous and aphasic patients will, therefore, probably score 1 or 0. The patient with brainstem stroke who has bilateral loss of sensation is scored 2. If the patient does not respond and is quadriplegic, score 2. Patients in a coma (item 1a=3) are automatically given a 2 on this item.	0= **Normal;** no sensory loss.<br>1= **Mild-to-moderate sensory loss;** patients feels pinprick is less sharp or is dull on the affected side; or there is a loss of superficial pain with pinprick, but patient is aware of being touched.<br>2= **Severe to total sensory loss;** patient is not aware of being touched in the face, arm and leg.
**9. Best language:** A great deal of information about comprehension will be obtained during the preceding sections of the examination. For this scale item, the patient is asked to describe what is happening in the attached picture, to name the items on the attached naming sheet and to read from the attached list of sentences. Comprehension is judged from responses here, as well as to all of the commands in the	0= **No aphasia;** normal<br>1= **Mild-to-moderate aphasia;** some obvious loss of fluency or facility of comprehension, without significant limitation on ideas expressed or form of expression. Reduction of speech and/or comprehension, however, makes conservation about provided materials difficult or impossible. For example, in conversation about provided materials, examiner can identify picture or naming card

preceding general neurological exam. If visual loss interferes with the tests, ask the patient to identify objects placed in the hand, repeat, and produce speech. The intubated patient should be asked to write. The patient in a coma (item 1a=3) will automatically score 3 on this item. The examiner must choose a score for the patient with stupor or limited cooperation, but a score of 3 should be used only if the patient is mute and follows no one-step commands.	content from patient's response.<br>2= **Severe aphasia;** all communication is through fragmentary expression; great need for inference, questioning, and guessing by the listener. Range of information that can be exchanged is limited; listener carries burden of communication. Examiner cannot identify materials provided from patient response.<br>3 = **Mute, global aphasia:** no usable speech or auditory comprehension.
**10. Dysarthria:** If patient is thought to be normal, an adequate sample of speech must be obtained by asking patient to read or repeat words from the attached list. If the patient has severe aphasia, the clarity of articulation of spontaneous speech can be rated. Only if patient is intubated or has other physical barriers to producing speech, the examiner should record the score as untestable (UN), and clearly write an explanation for this choice. Do not tell the patient why he or she is being tested.	**0= Normal.**<br>1= **Mild-to-moderate dysarthria;** patient slurs at least some words and, at worst, can be understood by some difficulty.<br>2= **Severe dysarthria:** patient's speech is so slurred as to be unintelligible in the absence of or out of proportion to any dysphasia, or is mute/anarthric.<br>UN = Intubated or other physical barrier.
**11. Extinction and Inattention** (formerly Neglect): Sufficient information to identify neglect may be obtained during the prior testing. If the patient has a severe visual loss preventing visual double simultaneous stimulation, and the cutaneous stimuli are normal, the score is normal. If the patient has aphasia but does appear to attend to both sides, the score is normal. The presence of visual spatial neglect or anosagnosia may also be taken as evidence of abnormality. Since the abnormality is scored only if present, the item is never untestable.	**0= No abnormality.**<br>1= **Visual, tactile, auditory, spatial, or personal inattention** or extinction to bilateral simultaneous stimulation in one of the sensory modalities.<br>2= **Profound hemi-inattention or extinction to more than one modality;** does not recognize own hand or orients to only one side of space.

Table 4 Barthel Index [40]

The Barthel index (BI) is an ordinal scale used to measure performance in 10 activities of daily living (ADL). Test scores range from 0 to 100, with higher scores indicating better performance in these activities.

Category	Scale definition
Feeding	0 = unable<br>5 = needs help cutting, spreading butter, etc., or requires modified diet<br>10 = independent
Bathing	0 = dependent<br>5 = independent (or in shower)
Grooming	0 = needs to help with personal care<br>5 = independent face/hair/teeth/shaving (implements provided)
Dressing	0 = dependent<br>5 = needs help but can do about half unaided<br>10 = independent (including buttons, zips, laces, etc.)
Bowels	0 = incontinent (or needs to be given enemas)<br>5 = occasional accident<br>10 = continent
Bladder	0 = incontinent, or catheterized and unable to manage alone<br>5 = occasional accident<br>10 = continent
Toilet use	0 = dependent<br>5 = needs some help, but can do something alone<br>10 = independent (on and off, dressing, wiping)
Transfers (bed to chair and back)	0 = unable, no sitting balance<br>5 = major help (one or two people, physical), can sit<br>10 = minor help (verbal or physical)<br>15 = independent
Mobility (on level surfaces)	0 = immobile or < 50 yards<br>5 = wheelchair independent, including corners, > 50 yards<br>10 = walks with help of one person (verbal or physical) > 50 yards<br>15 = independent (but may use any aid; for example, stick) > 50 yards
Stairs	0 = unable<br>5 = needs help (verbal, physical, carrying aid)<br>10 = independent

Guidelines

1. The index should be used as a record of what a patient does, not as a record of what a patient could do.
2. The main aim is to establish degree of independence from any help, physical or verbal, however minor and for whatever reason.
3. The need for supervision renders the patient not independent.
4. A patient's performance should be established using the best available evidence. Asking the patient, friends/relatives and nurses are the usual sources, but direct observation and common sense are also important. However direct testing is not needed.
5. Usually the patient's performance over the preceding 24-48 hours is important, but occasionally longer periods will be relevant.
6. Middle categories imply that the patient supplies over 50 per cent of the effort.
7. Use of aids to be independent is allowed.

Table 5 EUROQOL 5D-5L [39]

The EuroQoL 5-dimensions 5-level (EQ-5D-5L) questionnaire is a standardized measure of health outcome that has been used extensively in patients with stroke.

Under each heading, please tick the ONE box that best describes your health TODAY.

Mobility

I have no problems in walking about ☐

I have slight problems in walking about ☐

I have moderate problems in walking about ☐

I have severe problems in walking about ☐

I am unable to walk about ☐

Self-care

I have no problems washing or dressing myself ☐

I have slight problems washing or dressing myself ☐

I have moderate problems washing or dressing myself ☐

I have severe problems washing or dressing myself ☐

I am unable to wash or dress myself ☐

Usual activities (e.g. work, study, housework, family or leisure activities)

I have no problems doing my usual activities ☐

I have slight problems doing my usual activities ☐

I have moderate problems doing my usual activities ☐

I have severe problems doing my usual activities ☐

I am unable to do my usual activities ☐

Pain/discomfort

I have no pain or discomfort	☐
I have slight pain or discomfort	☐
I have moderate pain or discomfort	☐
I have severe pain or discomfort	☐
I have extreme pain or discomfort	☐

Anxiety/depression

I am not anxious or depressed	☐
I am slightly anxious or depressed	☐
I am moderately anxious or depressed	☐
I am severely anxious or depressed	☐
I am extremely anxious or depressed	☐

Table 6 Clot Burden Score for CTA and MRA [46]

No contrast agent filling	Score	
Supraclinoid internal carotid artery	2	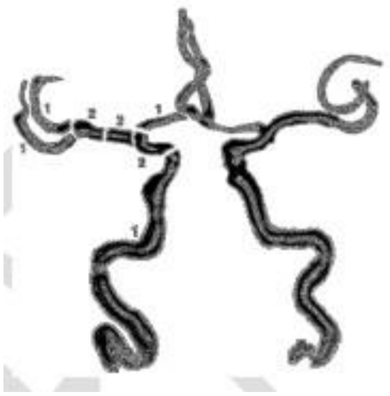
Proximal M1	2	
Distal M1	2	
Infraclinoid internal carotid artery	1	
A1 branch	1	
M2 branch	1	
**Total score: 10 – Sum**	**Total**	

Table 7 Collateral Score[43]

Category	Score	Description
**None**	0	Absent collaterals
**Poor**	1	Collaterals filling ≤50% of the occluded territory
**Intermediate**	2	Collaterals filing >50%, but <100% of the occluded territory
**Good**	3	Collaterals filling 100% of the occluded territory

Table 8 Classification pf Infarct in a New Territory [42]

Classification based on size		Classification based on catheter manipulation across territory ostium	
**Type I**	≤2 mm diffusion lesion (unidentifiable on NCCT)	**Type A**	Catheter was manipulated past the ostium of the new territory (e.g. large ACA infarct in a patient with an initial M1 occlusion): greater likelihood that infarct is related to the procedure
**Type II**	>2 mm to ≤ 20 mm lesion (potentially difficult to identify on CT scan)	**Type B**	Catheter was not manipulated past the ostium of the new territory (e.g. left PICA infarct in a patient with an initial right M1 occlusion): lower likelihood that infarct is related to procedure
**Type III**	Large (> 20 mm) infarct		

Glossary: NCCT: Non contrast computed tomography; CTA: Computed tomography angiogram; IAT: intra-arterial treatment; NIHSS: National Institutes of Health Stroke Scale.

Table 9 Report of Suspicious Medical Device Adverse Events

Report of Suspicious Medical Device Adverse Events

Report date: Code: ☐ ☐ ☐ ☐ ☐ ☐ ☐ ☐ ☐ ☐ ☐

Report source: ☐ Manufacturer ☐ Distributor ☐ User Unit name:

Contact address: Post code: Contact Tel.:

**A. Patient**			**C. Medical device**
1. Name	2. Age	3. Gender:<br>☐ Male ☐ Female	11. Product name:
4. Disease to be treated or expected effect:			12. Trade name:
**B. Overview of adverse event**			13. Registration No.:
5. Main conditions of the event:			14. Name of the manufacturer:<br>Address of the manufacturer:<br>Telephone of the manufacture:
6. Event occurrence date:<br>7. Time of discovery or knowledge:			15. Model/specification:<br>Product number:<br>Lot number:
8. Place where the medical device is actually used:<br>☐ Medical institution ☐ Home<br>☐ Others (please specify):			**15.** Operator:<br>☐ Professional ☐ Non-professional<br>☐ Patient ☐ Others (specific information):
9. Consequence<br>☐ Death____________________(specific time);<br>☐ Life threatening;<br>☐ Permanent injury to the functional structure of the body;			17. Expiration date:<br>18. Production date:<br>19. Discontinuation date:<br>20. Implantation date (if implanted):
☐ May lead to permanent injury to the functional structure of the body;<br>☐ Need internal and surgical treatment to avoid the			21. Preliminary cause analysis of the event:

above permanent injury;<br><br>☐ Others (details should be given in "Event description").	
10. Event description: (Including at least the device usage time, purpose of use, usage basis, usage situation, adverse event occurred, impact on the victim, treatment measures taken, and the joint use of devices)	22. Preliminary handling of the event:
	23. Reporting progress of the event<br>☐ User has been notified ☐ Manufacturer has been notified<br>☐ Distributor has been notified ☐ Pharmaceutical supervision department has been notified
	**D. Relevance evaluation**
	(1) Was there any reasonable chronological sequence between the using of medical device and occurred/possible injury event? Yes ☐ No ☐<br><br>(2) Did the occurred/possible injury event belong to the injury type that may be caused by the medical device used? Yes ☐ No ☐ Not clear ☐<br><br>(3) Could the occurred/possible injury event be explained by combining the effect of drug and/or device, patient's condition or other non-medical device factors?<br>Yes ☐ No ☐ Not clear ☐<br><br>Evaluation conclusion: Very likely ☐ Possible ☐ Doubtful □ Undeterminable ☐
	**E. AE assessment**
	24. Evaluation opinions of provincial monitoring technical site (attached pages are acceptable):
	25. Evaluation opinions of national monitoring technical site (attached pages are acceptable):

Reporter: Physician ☐ Technician ☐ Nurse ☐ Others ☐

Signature of reporter: **Prepared by China Food and Drug Administration**

15. Figure

Figure 1 DIRECT-MT Trial Logo

DIRECT-MT

Figure 2 Patient Flow in the Trial

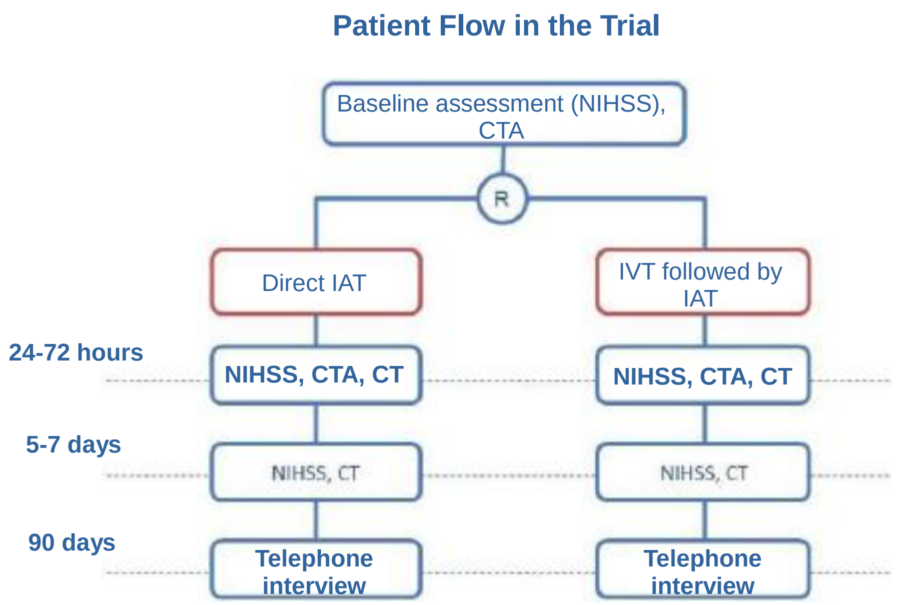

Glossary: CTA: Computed tomography angiogram; IAT: intra-arterial treatment; IVT: intravenous thrombolysis; NIHSS: National Institutes of Health Stroke Scale.

16. Appendix

16.1 Study committees

Steering Committee

Chairman: Prof Liu Jianmin, Changhai Hospital Affiliated to the Second Military Medical University

Members: Prof Deng Benqiang, Changhai Hospital Affiliated to the Second Military Medical University; Prof Yang Pengfei, Changhai Hospital Affiliated to the Second Military Medical University; Prof Zhang Yongwei, Changhai Hospital Affiliated to the Second Military Medical University; and Prof Hong Bo, Changhai Hospital Affiliated to the Second Military Medical University

Data Safety Monitoring Board

To be announced

Imaging Assessment Committee

To be announced

Adverse Event Adjudication Committee

16.2 DIRECT-MT recommendations of the Steering Committee with regard to type of mechanical thrombectomy and use of thrombolytic agents during endovascular procedures.

To be announced

General

Inclusion in the trial, randomization, and subsequent endovascular treatment with/without prior IVT should be started as soon as possible after presentation in all eligible patients. The time-path below gives an indication about how soon the following steps need to take place in the most optimal situation.

The optimal time-path for treatment and inclusion in DIRECT-MT of patients with acute ischemic stroke and relevant intracranial large vessel occlusion of the anterior circulation is listed below. The target time from study non-contrast CT to groin puncture will be as fast as possible. All patients must undergo groin puncture within a median of 60 minutes after non-contrast CT acquisition.

Procedures	Time path
**Arrival at ER**	0
**Randomization**	10
**Start neuroimaging**	10 min
**Start IV alteplase**	20 min

**(if so randomized)**	
**Groin puncture**	70 min

Neuroimaging

Neuroimaging studies to assess vessel patency should be done before or simultaneously with treatment with intravenous (IV) alteplase, in order not to lose time and brain. We aim to not cause any delay prior to intra-arterial treatment, by infusion of IV alteplase.

Additional thrombolytic agents, dose and type

If deemed indicated by the interventionist, local application (intra-arterial) alteplase is allowed in any of the patients included in the DIRECT-MT.

Patients who have been pre-treated with IV alteplase should not receive more than 30 mg alteplase during intra-arterial treatment. The steering committee recommends that the alteplase is delivered in shots of 5 mg, in 5-10 minutes time intervals. In individual cases, an equivalent dose of 400,000 U urokinase, delivered in shots of 50.000 - 100.000 U, in 5-10 minutes time intervals, is also accepted as escape medication. Vessel patency should be checked after each shot.

Type of mechanical thrombectomy device(s)

All stent retriever and aspiration devices for IAT, which are approved for this purpose by CFDA, and have been approved for use in the study by the steering committee are allowed in the trial as a first line of defense and are listed below:

Device name	Manufacturer	Description
**Solitaire**	Medtronic / Covidien	Retrievable stent
**Trevo stent**	Stryker	Retrievable stent
**Revive stent**	Codman/DePuy-Synthes	Retrievable stent

A second device is allowed as a second option, when the first device has failed according to the interventionist. The further choice of the particular device for a certain patient is left to the discretion of the interventionist.

16.3 Imaging requirements

16.3.1 Minimum baseline imaging requirements

When

1) Before randomization, a NCCT and CTA should be performed to assess eligibility for the study.

How

1. Pre-randomization NCCT:
    1. The thickness of the NCCT scanning layer is recommended to be 5 mm, and 5-8 mm is also acceptable.
    2. The NCCT study should include the whole head.
2. Pre-randomization CTA:
    1. The CTA study should cover the whole area from the aortic arch to the vertex, and intracranial part only is also acceptable.
    2. The CTA study should include thin slices (maximum of 1.0 mm)
    3. The CTA study should include the following reconstructions
        - i. Axial maximum intensity projection (MIP),
            1. MIP slab thickness: 25 mm
            2. Overlap: 5 mm
        - ii. Coronal MIP
            1. MIP slab thickness: 25 mm
            2. Overlap: 5 mm
3. After acquisition
    1. All images (both NCCT and CTA) should be saved to the DICOM format
    2. All available series should be sent to the core lab for assessment, including thin slice series (for thrombus assessment).

16.3.2 Intervention-related angiographic imaging

When

1) Before the intervention complete AP and Lateral angiograms (of whole head and including venous phase) should be performed to evaluate the site of vessel occlusion, extent of thrombus, territories involved, concomitant pathologies and to assess collateral flow.

2) After each passage of a mechanical or aspirational device, a control angiogram should be performed.

3) After each bolus of (a rescue) thrombolytic agent, a control angiogram should be performed.

4) At the end of the procedure complete AP and Lateral angiograms (of whole head and including venous phase) should be repeated. Without these complete runs, optimal TICI scoring is not possible

How

Pre-intervention and end-of-procedure angiogram:

a. Angiograms should be performed through the guiding catheter

b. Baseline and final AP views and lateral views of the intracranial arteries are mandatory. Both are required to assess reperfusion after the procedure.

c. Baseline and final angiograms should include both the arterial and venous phases of the injection to evaluate the collateral pathways and perfusion of the distal vascular bed.

d. Baseline and final angiograms should include the internal carotid artery feeding the target vessel as demonstrated on CTA.

e. Baseline and final angiograms should include the common carotid and internal carotid artery in case of occlusion, dissection or severe stenosis in the carotid feeding the target vessel as demonstrated on CTA.

f. Angiograms should be performed via the guiding catheter with the same catheter position and same views before and after the procedures to adequately assess the results of therapy.

After each device placement:

g. A non-contrast radiograph should be obtained

h. At least one view at the discretion of the interventionalist

After each passage of mechanical or aspirational device or bolus of (rescue) thrombolytic agent :

i. Angiograms should be performed through the guiding catheter

j. At least one view, at the discretion of the interventionalist.

After the procedure

k. Complete series of the angiograms and microcatheter injections (when performed) should be saved according to the DICOM standard.

l. All series should be forwarded to the imaging assessment committee.

16.3.3 Minimum follow-up imaging requirements

When

1) 24-72 hours after undergoing endovascular treatment, a NCCT and CTA should be performed to assess treatment efficacy.
2) 5-7 days after undergoing endovascular treatment, or before discharge a NCCT should be performed to assess final lesion volume and potential hemorrhages complications.
3) If clinically required (i.e. in cases of clinical deterioration of the patient) additional imaging as needed, at the discretion of the treating physician is acquired.

How

24-72 hours NCCT:

1. The thickness of the NCCT scanning layer is recommended to be 5 mm, and 5-8 mm is also acceptable.
2. The NCCT study should include the whole head.

24-72 hours CTA:

3. The CTA study should cover the whole area from the aortic arch to the vertex, and intracranial part only is also acceptable.
4. The CTA study should include thin slices (maximum of 1.0 mm)
5. The CTA study should include the following reconstructions
    i. Axial maximum intensity projection (MIP),
        1. MIP slab thickness: 25 mm
        2. Overlap: 5 mm
    ii. Coronal MIP
        1. MIP slab thickness: 25 mm
        2. Overlap: 5 mm

5-7 days NCCT (or before discharge)

6. The NCCT study should contain both thick (5mm) and thin slices (maximum of 2.5mm).
7. The NCCT study should include the whole head.
8. In addition, clinically required imaging is at the discretion of the treating physician.
9. After acquisition, all images (NCCT, CTA, and additional imaging) should be saved to

the DICOM file format

10. All available series should be sent to the core lab for assessment, including thin slice series (for thrombus assessment).

Direct Intra-arterial thrombectomy in order to Revascularize AIS patients with large vessel occlusion Efficiently in Chinese Tertiary hospitals: a Multicenter randomized clinical Trial (DIRECT-MT)

Project leaders:

Liu Jianmin,Deng Benqiang

Charles Majoie, Yvo Roos

Sponsor: Changhai Hospital Affiliated to the Second Military Medical University

CRO: Cardiovascular Chinese Research Center

Protocol No.: CH01

Version No.: V3.0

Date: August 20, 2019

Statement of Confidentiality

The information contained in this document involves trade secrets and business information, which is confidential or proprietary and shall not be disclosed by any person unless required by regulations. Any individual who has access to the document shall be aware that the document is proprietary or confidential and shall not disclose the document without authorization. The above restrictions on the confidentiality of the document will apply to any proprietary or confidential data provided to you in the future.

Protocol title:

Direct Intra-arterial thrombectomy in order to Revascularize AIS patients with large vessel occlusion Efficiently in Chinese Tertiary hospitals: a Multicenter randomized clinical Trial (DIRECT-MT)

Protocol ID	CH01
Version	3.0
Date	August 20, 2019
Project leaders	Prof dr Liu Jianmin, Changhai Hospital Affiliated to the Second Military Medical University<br><br>Prof dr Deng Benqiang, Changhai Hospital Affiliated to the Second Military Medical University<br><br>Prof dr Charles Majoie, Academisch Medisch Centrum bij de Universitieit van Amsterdam (AMC)<br><br>Prof dr Yvo Roos, Academisch Medisch Centrum bij de Universitieit van Amsterdam (AMC)
Coordinating investigators	Prof dr Yang Pengfei, Changhai Hospital Affiliated to the Second Military Medical University<br><br>Prof dr Zhang Yongwei, Changhai Hospital Affiliated to the Second Military Medical University
Sponsor	Changhai Hospital Affiliated to the Second Military Medical University
Subsidizing parties	Stroke Prevention and Control Engineering Commission of National Health and Family Planning Commission of the People's Republic of China, Wu Jieping Medical Foundation
CRO	Cardiovascular Chinese Research Center

Protocol Signature Sheet

Name	Signature	Date
Sponsor:<br><br>Changhai Hospital Affiliated to the Second Military Medical University		

I have read this trial protocol carefully and recognize that this protocol covers all the necessary contents for the implementation of the trial. I will conduct the study according to the protocol and complete the study within the specified period of time.

I will provide copies of this study protocol and all relevant information to all staff who assist me in conducting this study. I will discuss these materials with them to ensure that they fully understand the test drug and how to conduct the trial.

Name	Signature	Date
[Principal investigators]:		

Contents

List of abbreviations and definitions of terms **56**

SUMMARY **58**

1. Introduction and rationale **60**

2. Study objectives **61**

3. Study design **61**

4. Study population **61**

4.1. Population (Base) 61

4.2. Participating centers and center eligibility 61

4.3. Inclusion criteria 62

4.4. Exclusion criteria 62

4.5. Sample size calculation 63

5. Treatment of subjects **63**

5.1. Investigational treatment 63

5.2. Use of co-intervention 64

5.3. Escape medication 64

6. Investigational product **64**

6.1. Name and description of investigational product 64

6.2. Summary of findings from clinical studies 64

6.3. Summary of known and potential risks and benefits 65

6.4. Description and justification of route of administration and dosage 65

7. Non-investigational product **65**

7.1. Name and description of non-investigational products 65

7.2. Summary of findings from clinical studies 65

7.3. Summary of known and potential risks and benefits 65

8. Method **65**

8.1. Study outcomes 65

8.1.1. Main study outcome 65

8.1.2. Secondary outcomes 66

8.1.3. Safety outcomes 66

8.1.4. Other study parameters 66

8.2. Randomization, blinding and treatment allocation 67

8.3. Study procedures 67

8.4. Withdrawal of individual subjects 67

8.5. Premature termination of the study 67

9. Safety reporting **68**

9.1. Temporary halt for reasons of subject safety 68

9.2. AEs, SAEs and SUSARs 68
9.2.1. Adverse events (AEs) 68
9.2.2. Serious adverse events (SAEs) 68
9.3. Follow-up of adverse events 69
9.4. Data Safety Monitoring Board (DSMB) 69
10. Statistical analysis 69
10.1. Statistical analysis 69
10.2. Subgroup analysis 70
10.3. Interim analysis 70
11. Ethical considerations 70
11.1. Regulation statement 70
11.2. Recruitment and consent 70
11.3. Problems of minors or incapacitated subjects 71
11.4. Benefits and risks assessment, group relatedness 71
11.5. Compensation for injury 71
12. Administrative aspects, monitoring and publication 71
12.1. Handling and storage of data and documents 71
12.2. Monitoring and quality assurance 71
12.3. Amendment 72
12.4. Annual progress report 72
12.5. Temporary halt and (prematurely) end of study report 72
12.6. Public disclosure and publication policy 72
13. References 73
14. Table 77
Table 1 Modified Rankin Scale [35] 77
Table 2 Extended Treatment In Cerebral Ischemia (Etici) Scale [36] 78
Table 3 NIH Stroke Scale 79
Table 4 Barthel Index [40] 83
Table 5 EUROQOL 5D-5L [39] 85
Table 6 Clot Burden Score for CTA and MRA [46] 87
Table 7 Collateral Score[43] 87
Table 8 Classification pf Infarct in a New Territory [42] 88
Table 9 Report of Suspicious Medical Device Adverse Events 89
15. Figure 91
Figure 1 DIRECT-MT Trial Logo 91
Figure 2 Patient Flow in the Trial 91
16. Appendix 92
16.1 Study committees 92

Steering Committee 92
Data Safety Monitoring Board 92
Imaging Assessment Committee 92
Adverse Event Adjudication Committee 92
Outcome Committee 93
16.2 DIRECT-MT recommendations of the Steering Committee with regard to type of mechanical thrombectomy and use of thrombolytic agents during endovascular procedures. 93
General 93
Neuroimaging 93
Additional thrombolytic agents, dose and type 93
Type of mechanical thrombectomy device(s) 93
16.3 Imaging requirements 94
16.3.1 Minimum baseline imaging requirements 94
When 94
How 94
16.3.2 Intervention-related angiographic imaging 94
When 94
How 95
16.3.3 Minimum follow-up imaging requirements 96
When 96
How 96

List of abbreviations and definitions of terms

AE	Adverse event
AIS	Acute ischemic stroke
AR	Adverse Reaction
ASA	Acetyl salicylic acid
CT	Computed tomography
CTA	Computed tomography angiography
CV	Curriculum Vitae
DSMB	Data Safety Monitoring Board
EC	Ethics committee
EU	European Union
GCP	Good Clinical Practice
IAT	Intra-arterial treatment
IB	Investigator's Brochure
ICF	Informed Consent Form
ICH	Intracerebral hemorrhage
IMP	Investigational Medicinal Product
IU	International standard unit
IV	Intravenous
MRI	Magnetic resonance imaging
NIHSS	NIH Stroke Scale test
(S) AE	(Serious) adverse event
sICH	Symptomatic intracerebral hemorrhage
Sponsor	The sponsor is the party that commissions the organization or performance of the research, for example a pharmaceutical company, academic hospital, scientific organization or investigator. A party that provides funding for a study but does not commission is not regarded as the sponsor, but referred to as a subsidizing party.

SUSAR	Suspected unexpected serious adverse reaction
tPA	Tissue plasminogen activator

SUMMARY

Protocol title: Direct Intra-arterial thrombectomy in order to Revascularize AIS patients with large vessel occlusion Efficiently in Chinese Tertiary hospitals: a Multicenter randomized clinical Trial (DIRECT-MT)

Rationale: Intra-arterial treatment (IAT) by means of retrievable stents has been proven safe and effective in patients with acute ischemic stroke with confirmed large vessel occlusion of the anterior circulation and in whom the procedure can be started within 6 hours from onset. Despite recanalization, a considerable proportion of patients do not recover. This can be attributed to potential adverse effects of the intravenous treatment (IVT) prior to IAT. These effects could include neurotoxicity, blood brain barrier leakage and thrombus fragmentation through softening of the thrombus.

Another reason for non-recovery in MRCLEAN was the occurrence of symptomatic intracranial hemorrhage (sICH) in 7% of patients, which was fatal in 65%. sICH occurred as often in the intervention as in the control group, suggesting that this complication could not be attributed to the IAT, but rather to pre-treatment with IVT. The HERMES study showed that the incidence of symptomatic intracranial hemorrhage was about 4.4% in the western population. Considering the high rate of intracranial atherosclerosis in Chinese population, the clinical prognosis after thrombectomy may be slightly better. Therefore, we hypothesize that direct IAT may lead to a 4% absolute increase in good outcome compared to IAT preceded by IVT.

Objective: To assess the effect of direct IAT compared to IVT followed by IAT, in patients with acute ischemic stroke, caused by a CTA-confirmed occlusion of the anterior circulation (intracranial segment of ICA, M1, proximal M2) on functional outcome.

Study design: This is a parallel group, randomized clinical trial of direct IAT versus IVT with IAT. The trial has observer blind assessment of the primary outcome and of neuro-imaging at baseline and follow up.

Study population: Patients with acute ischemic stroke and a confirmed anterior circulation occlusion by CTA. Initiation of IVT must be feasible within 4.5 hours from symptom onset. Age must be 18 or over and NIHSS 2 or more.

INCLUSION CRITERIA

- a clinical diagnosis of acute ischemic stroke,
- caused by a large vessel occlusion of the anterior circulation (intracranial segment of ICA or middle M1/proximal M2) cerebral artery confirmed by CTA,
- CT or MRI ruling out intracranial hemorrhage,
- eligible for IVT and IAT (within 4.5 hours after symptom onset),
- a score of at least 2 on the NIH Stroke Scale,

- age of 18 years or older,
- written informed consent.

EXCLUSION CRITERIA

- Pre-stroke disability which interferes with the assessment of functional outcome at 90 days, i.e. mRS >2
- Any contra-indication for IVT, according to guidelines of the American Heart Association, i.e.:
    - arterial blood pressure exceeding 185/110 mmHg
    - blood glucose less than 2.7 or over 22.2 mmol/L
    - cerebral infarction in the previous 6 weeks with residual neurological deficit or signs of recent infarction on neuro-imaging
    - serious head trauma in the previous 3 months
    - major surgery or serious trauma in the previous 2 weeks
    - gastrointestinal or urinary tract hemorrhage in the previous 3 weeks
    - previous intracerebral hemorrhage
    - use of anticoagulant with INR exceeding 1.7
    - known thrombocyte count less than 100 x 10^9/L
    - treatment with direct thrombin or factor X inhibitors
    - treatment with heparin (APTT exceeds the upper limit of normal value) in the previous 48 hours.

Intervention: The intervention group will undergo immediate IAT using a stent retriever, as recommended by the steering committee. The standard care group will receive IVT 0.9 mg/kg with a maximum dose of 90 mg in one hour, followed by IAT using a stent retriever. We strive to reduce delays associated with IVT administration to a minimum to adequately assess the effect of IVT itself with IAT.

Main study parameters/outcomes: The primary effect parameter will be the common odds ratio, estimated with ordinal logistic regression, which represents the shift on the full distribution of the modified Rankin Scale at 3 months. The estimate will be adjusted for the known prognostic variables age, pre-stroke mRS, time from onset to randomization, stroke severity (NIHSS) and collaterals and adjusted and unadjusted estimates with corresponding 95% confidence intervals will be reported.

Secondary outcomes include mortality at 90 days, stroke severity at 24$\pm$6 hours and 5-7 days, recanalization on CTA at 24-72 hours, dichotomous clinical outcome on the mRS and infarct size at 5-7 days. Safety outcomes include rate of sICH.

1. Introduction and rationale

Stroke is a major cause of death and disability. The latest National Epidemiological Survey of Stroke in China [1] (Ness-China) showed that: the standardized prevalence, incidence and mortality of stroke in China in 2013 were 1114.8/100 thousand person/year, 246.8/100 thousand person/year and 114.8/100 thousand person/year respectively. In 1985, the prevalence of stroke in China was only 365/100 thousand person/year [2]. In the case of a gradual decline in the incidence and mortality of stroke in European and American countries, the incidence of Chinese people gradually increased at a rate of 8.7% per year, which was significantly higher than the overall annual incidence of stroke in the world [3-5].

Early 2015, the outlook of acute stroke changed dramatically over the course of a few months. It was shown that patients with acute ischemic stroke (AIS) caused by a large vessel occlusion of the anterior circulation benefit from intra-arterial treatment (IAT). IAT using a stent retriever leads to an absolute increase in good functional outcome in 15% to 25% of patients treated within 6 hours. This was first reported in the MR CLEAN trial and later confirmed in 4 other trials [6-10].

In randomized trials of acute ischemic stroke, intravenous thrombolysis (IVT) with alteplase strongly reduced the risk of a poor outcome [11, 12]. However, two thirds of the patients treated with IVT within 3 hours of stroke onset in these trials were dead or dependent at the end of follow-up. In the MR CLEAN trial, 67% of the patients in the endovascular treatment group were dead or dependent at three months. The high risk of a poor outcome, even after these acute revascularization strategies, may to a large extent be explained by no-reflow. No-reflow has been linked to distal micro vascular damage or dysfunction as a result of tissue necrosis and cell death, or the intervention simply being late.

Currently the role of IVT in acute ischemic stroke treatment with IAT is unclear. The incidence of bleeding complications was similar in MR CLEAN to the frequency in the NINDS IVT trial and SITS MOST registry [13, 14]. In MR CLEAN, the occurrence of symptomatic intracranial hemorrhage (sICH) (7%, fatal in 65%) was similar between the intervention and the control group, suggesting that this complication could not be attributed to the IAT, but rather to pre-treatment with IVT. According to the meta-analysis of the five RCT results, the incidence of symptomatic intracranial hemorrhage in westerners was 4.4%. However, there are differences in the pathogenesis of stroke between eastern and western populations. In 2017, a retrospective ACTUAL study based on Chinese population showed that 44.3% acute intracranial artery occlusion is caused by atherosclerosis, which was significantly higher than westerners. At the same time, there was no significant difference in the incidence of sICH between direct endovascular treatment group and bridging treatment group. This may remind us that the increased proportion of acute intracranial atherosclerotic occlusion did not significantly influence the incidence of sICH. Whether the increase of the stent implantation proportion will affect clinical outcome is unknown. In the ACTUAL study, the incidence of aICH in the intravascular treatment group was significantly lower than that in the bridging treatment group (28.3% vs. 44.9%, P=0.01). whether the increase in the proportion of stent implantation will increase the incidence of ICH after IVT, which are currently unknown and need to be studied.

According to the above comprehensive analysis, we hypothesize that direct IAT, without

pretreatment with IVT, in selected patients may lead to a 4% absolute increase in good outcome because of a reduction in the occurrence of sICH and an increase in treatment effect of IAT.

MR CLEAN is the earliest and only completed RCT study on the evaluation of the efficacy of IAT. This study intends to conduct in-depth cooperation with MR CLEAN study team in the Netherlands, and conducts an international prospective multi-center randomized controlled study in both locations to explore the differences in the clinical outcome between the two to answer the concept whether the clinical outcome of this direct IAT is better than that of the current treatment by comparing direct IAT with IVT and IAT bridging treatments, and the efficacy of stents in different populations [(12)].

2. Study objectives

The primary objective of this trial is to assess the effect of direct IAT compared with IVT followed by IAT, on functional outcome in patients with AIS, caused by an anterior circulation occlusion that is confirmed by CTA.

The secondary objective is to explore for superiority of direct IAT relative to IVT followed by IAT.

The tertiary objective is to assess the effect of direct IAT compared with IVT with IAT on neurological recovery (NIHSS), infarct size and occurrence of sICH.

The fourth objective is to collect thrombi and to analyze them with respect to their potential for treatment effect modification.

3. Study design

This is a multicenter phase IV prospective randomized clinical trial with open-label treatment and blinded outcome assessment (PROBE). The study will run for 4 years in intervention centers.

4. Study population

4.1. Population (Base)

The latest National Epidemiological Survey of Stroke in China [(1)] (Ness-China) showed that: the standardized prevalence, incidence and mortality of stroke in China in 2013 were 1114.8/100 thousand person/year, 246.8/100 thousand person/year and 114.8/100 thousand person/year respectively. In 1985, the prevalence of stroke in China was only 365/100 thousand person/year [(2)]. In the case of a gradual decline in the incidence and mortality of stroke in European and American countries, the incidence of Chinese people gradually increased at a rate of 8.7% per year, which was significantly higher than the overall annual incidence of stroke in the world [(3-5)].

4.2. Participating centers and center eligibility

To be fully eligible for participation in the trial and to include patients in the trial, centers should meet the following minimum criteria:

- Local tertiary hospitals;

- Centers with experience in conducting acute stroke trials;
- It can simultaneously perform intravenous thrombolysis and endovascular thrombectomy, and completes more than 30 endovascular treatment of acute ischemic stroke each year,
- The intervention team should have experience with endovascular interventions for cerebrovascular disease (IAT, carotid stenting or aneurysm coiling), peripheral artery disease, or coronary artery disease, and the stroke team (which includes neurologists and interventionists) should have previous experience with intra-arterial treatment,
- The intervention team should make use of one or more of the devices that have been approved by CFDA. Use of other devices is not allowed in the trial.
- At least one member of the intervention team should have previous experience with the particular device.

Note: Patients may only be included in the trial when the intervention team that will actually treat the patient includes at least one interventionist with previous experience with IAT.

4.3. Inclusion criteria

In order to be eligible to participate in this study, a subject must meet all of the following criteria:

- a clinical diagnosis of acute ischemic stroke;
- caused by a large vessel occlusion of the anterior circulation (distal intracranial carotid artery or middle M1/proximal M2) cerebral artery confirmed by CTA;
- CT or MRI ruling out intracranial hemorrhage;
- eligible for IVT and IAT (within 4.5 hours after symptom onset);
- NIHSS ≥ 2;
- age of 18 years or older;
- written informed consent.

4.4. Exclusion criteria

A potential subject who meets any of the following criteria will be excluded from participation in this study:

- Pre-stroke disability which interferes with the assessment of functional outcome at 90 days, i.e. mRS >2;
- Any contra-indication for IVT, according to guidelines of the American Heart Association [27], i.e.:
  - blood pressure > 185/110 mmHg,
  - blood glucose < 2.7 or > 22.2 mmol/L,

- cerebral infarction in the previous 6 weeks with residual neurological deficit or signs of recent infarction on neuro-imaging,
- serious head trauma in the previous 3 months,
- major surgery or serious trauma in the previous 2 weeks,
- gastrointestinal or urinary tract hemorrhage in the previous 3 weeks,
- previous intracerebral hemorrhage,
- use of anticoagulant with INR exceeding 1.7,
- known thrombocyte count less than 100 × 10^9/L
- treatment with direct thrombin or factor X inhibitors,
- treatment with heparin (APTT exceeds the upper limit of normal value) in the previous 48 hours.

4.5. Sample size calculation

We based our estimations on the distribution of the modified Rankin Scale (mrS) in the control group of the trial, which we derived from the intervention group of the MR CLEAN trial [(9)]: mRS 0: 3%; mRS 1: 9%; mRS 2: 21%; mRS 3: 18%; mRS 4: 22%; mRS 5: 6% and mRS 6: 21%. We assumed a favorable treatment effect with a common odds ratio (cOR) of 1.163, corresponding to a 4% absolute increase in the rate of mRS scores of 0-2. The main purpose is to demonstrate non-inferiority, that is, the lower limit of the 95% confidence interval does not cross the pre-specified cOR non-inferiority Cutoff of 0.8. In a simulation with 5000 runs we computed the proportion of positive trials, for a given sample size. A sample size of 710 was determined to detect the pre-defined non-inferiority with a power of 80% and two-sided alpha of 0.05. Using covariate adjustment with at most 25%, a conservative 15% sample size reduction can be achieved, plus 5% dropout rate, leading to a final sample size of 636, 318 per arm.

5. Treatment of subjects

5.1. Investigational treatment

Patients in the control group will receive IVT (alteplase) according to the guidelines of the American Heart Association. [(27)] Patients in the intervention group will not receive this treatment (nor placebo) and proceed directly with IAT. Patients in both groups will undergo IAT. Please note that to assess the effect of IVT itself and not the applied treatment strategy, we strive to reduce delays in the control group due to IVT administration to an absolute minimum. Remaining differences between treatment groups in time from randomization to groin puncture will be recorded. All stent retriever devices for IAT, which are approved by CFDA for this purpose, are allowed in the trial as a first line of defense.

Other mechanical devices (aspiration devices) are allowed as a second option, when the first device has failed according to the interventionist, usually after 3 passes. The further choice of the particular

device for a certain patient is left to the discretion of the interventionist.

The target time from study randomization to groin puncture will be as fast as possible. All patients must undergo groin puncture within a median of 60 minutes after randomization.

5.2. Use of co-intervention

No standard co-medication is advised by the steering committee. Antiplatelet or antithrombotic treatment will generally be started at 24 hours after the intervention, according to national protocols.

5.3. Escape medication

If deemed by the interventionist, local application (intra-arterial) of alteplase is allowed in any of the patients included in the DIRECT-MT. Patients in the direct IAT group in whom good recanalization (eTICI 2b-3) was not reached, may be treated afterwards with 0.9 mg/kg IVT if the 4.5 hour window or maximum dose is not exceeded. Patients who have been pre-treated with i.v. alteplase should not receive more than 30mg alteplase during intra-arterial treatment. The steering committee recommends that the alteplase is delivered in shots of 5 mg in 5-10 minute intervals.

In individual cases, an equivalent dose of 400,000 U urokinase, delivered in shots of 50.000 - 100.000 U, in 5-10 minutes time intervals, is also accepted as escape medication.

Vessel patency should be checked after each shot.

6. Investigational product

6.1. Name and description of investigational product

The comparator in this trial is IVT with alteplase (actilyse). The intervention is omitting IVT before IAT.

6.2. Summary of findings from clinical studies

The value of IVT in patients with AIS has been determined in multiple RCTs with a potential treatment window up to 4.5 hours after symptom onset [30, 31]. It has been a standard care for several years. All trials investigating the benefit of IAT in AIS had a control group consisting of patients receiving usual care [6-9, 32]. This meant that few patients were treated directly, without prior IVT. In MR CLEAN, this concerned only 55 patients (11%). Subgroup analysis showed a similar effect size in patients not treated with IVT (OR = 2.06 [95% Confidence Interval (CI): 0.69-6.13]) as in patients pretreated with IVT (OR =1.71 [95% CI: 1.22-2.40]). [9] REVASCAT showed comparable results: 56 patients not treated with IVT (OR = 2.6 [95% CI: 1.0-7.1]) as to 76 patients who were pretreated (OR = 1.4 [95% CI: 0.8-2.6]). [7] Moreover, in ESCAPE, patients without IV pretreatment seemed to benefit (OR = 2.6 [95% CI: 1.1-5.9]) from endovascular treatment. [6] When we combined the published data there is no heterogeneity (p= 0.78). In a fixed effect model, the effect estimate is quite precise and statistically significant (OR = 2.3 [95% CI: 1.5-3.7]). We believe that the data from these three randomized controlled trials show that patients not pretreated with IVT may benefit from intervention.

6.3. Summary of known and potential risks and benefits

For known possible undesirable effects of actilyse, see the summary of product characteristics supplied.

6.4. Description and justification of route of administration and dosage

The route and dosage of administration are based on the American Heart Association guidelines.

7. Non-investigational product

7.1. Name and description of non-investigational products

Stent-retrievers for IAT are the background treatment in this trial. The devices approved by CFDA during the research may be used as primary device for IAT (not limited to the table listed below)

Device name	Manufacturer	Description
**Solitaire**	Medtronic / Covidien	Retrievable stent
**Trevo**	Stryker	Retrievable stent
**Revive**	Johnson & Johnson/ Cerenovus	Retrievable stent

7.2. Summary of findings from clinical studies

Seven randomized clinical trials that predominantly used stent thrombectomy have been carried out. [6-10, 33, 34] All trials showed a beneficial effect of intervention compared to usual care, which most often included treatment with iv-alteplase. The effect size ranged from 11 to approximately 25% increase in proportion of non-disabled patients at 3 months after randomization. [6-10, 33, 34]The treatment is already established as standard of care. [32] As stated in paragraph 7.3, the subgroup analyses of recent trials suggest that patients not pretreated with IVT may benefit from intervention.

7.3. Summary of known and potential risks and benefits

The potential benefits of the intervention have been described in 3.3. The potential risks consist of intracranial and extracranial hemorrhage and hemorrhagic infarction, procedure related risks such as dissection, perforation and infarctions in other vascular territories, and postprocedural events such as infections. In the 5 trials, the risks of hemorrhage and hemorrhagic infarction were equal for both the intervention group as the control group. Postprocedural events such as pneumonia and other infections occurred in similar frequencies in both groups, and procedure-related events were infrequent.

8. Method

8.1. Study outcomes

8.1.1. Main study outcome

The primary outcome is the score on the modified Rankin Scale (Table 1 in Appendix) at 90 days (± 14 days). [35] The mRS is the preferred disability parameter for clinical trials in stroke. The mRS is an ordinal hierarchical scale incorporating six categories from 0 up to and including 5, and describes the range of disability encountered post stroke. 'Death' is assigned a score of 6. Assessment of outcome on the mRS will be performed by independent assessors, blinded to the allocated and actually received treatment. Their assessment will be based on standardized reports of a telephone interview by trained research personnel who are not aware of treatment allocation.

8.1.2. Secondary outcomes

Secondary outcomes are the following:

- Death within 90 days (± 14 days)
- Pre-interventional recanalization
- eTICI score on final angiography of IAT. [36] (Table 2 in Appendix)
- Recanalization rate at 24-72 hours, assessed with CTA
- Score on the NIHSS at 24±6 hours and 5-7 days. [37] (Table 3 in Appendix)
- Final infarct volume at 5-7 days. Final infarct volume will be assessed with the use of an automated, validated algorithm. [38] Infarct size at day 5-7 will be compared with plain CT and perfusion CT results (if available) at baseline.
- Dichotomized mRS of 0-1 vs. 2-6 at 90 days (± 14 days)
- Dichotomized mRS of 0-2 vs. 3-6 at 90 days (± 14 days)
- Dichotomized mRS of 0-3 vs. 4-6 at 90 days (± 14 days)
- Score on the EQ5D-5L and Barthel index at 90 days (± 14 days) [39] [40]

8.1.3. Safety outcomes

- Hemorrhages according to the Heidelberg criteria [40]
- sICH scored according to the Heidelberg criteria [41]
- Embolization in new territory on angiography during IAT
- Occurrence of aneurysma spurium
- Occurrence of groin hematoma
- Infarction in new territory at 5-7 days [42] (Table 8 in Appendix)
- Death from all causes within 90 days (± 14 days)

8.1.4. Other study parameters

Baseline parameters that will be recorded include age; sex; previous stroke; conditions such as hypertension, diabetes mellitus, atrial fibrillation, myocardial infarction; smoking status; medication including antiplatelet agents and anticoagulants; vital parameters such as blood pressure, body

temperature; weight and height; neurological examinations including NIHSS; laboratory examination including INR, APTT, PLT, glucose, creatinine; and imaging results on admission (e.g. clot burden score, table 6 in Appendix).

We will record the actually received dose, type and timing of iv thrombolytic medication.

Additionally, we will record time from onset to ER, CT, randomization, start of IAT, first reperfusion and end of procedure. The devices and the order in which they are used will be recorded, and the type of anesthesia (if any) and sedation will be noted.

8.2. Randomization, blinding and treatment allocation

The randomization procedure will be computer and web-based. Randomization is allowed when the occlusion has been established by CTA. Randomization will be stratified by center.

It will not be possible to view the treatment allocation before the patient is registered in the study database, nor will it be possible to remove the patient from the study after treatment assignment has become known. Both patient and treating physician will be aware of the treatment assignment. Information on outcome at three months will be assessed through standardized forms and procedures, by a trained investigator blinded for treatment allocation. Interviews will be recorded. Assessors who are blinded to the treatment allocation will perform assessment of outcome on the modified Rankin scale on this information. Results of neuro-imaging will be also assessed in a blinded manner. Information on treatment allocation will be kept separate from the main study database. The steering committee will be kept unaware of the results of interim analyses of efficacy and safety. An independent trial statistician will combine data on treatment allocation with the clinical data in order to report to the data monitoring committee (DSMB).

8.3. Study procedures

All patients will undergo assessment of the NIHSS at baseline, 24±6 hours and 5-7 days, which is routine in clinical procedure. It will be carried out by certified assessors. Patients will undergo NCCT and CTA at baseline. After 24-72 hours CTA is repeated to determine recanalization. At 5-7 days, patients will undergo NCCT to assess infarct size.

In addition, this trial also makes use of “waste material”: retrieved thrombi during intervention. These thromboses will be stored in the participating study centers for follow-up analysis.

8.4. Withdrawal of individual subjects

Subjects can leave the study at any time for any reason if they wish to do so without any consequences. The investigator can decide to withdraw a subject from the study for urgent medical reasons. The data from subjects who do not provide consent will be treated anonymously, and used for baseline analysis to further describe this population. At the time of analysis, missing data are interpolated, including the final mRS score. The key part of personal data will be cleared.

8.5. Premature termination of the study

The study will only be terminated prematurely if the Data Safety Monitoring Board recommends stopping. In case of premature termination of the study, the database will be closed after 90 days assessment of the last enrolled patient and results will be reported.

9. Safety reporting

9.1. Temporary halt for reasons of subject safety

The sponsor will suspend the study if there is sufficient ground that continuation of the study will jeopardize subject health or safety. The sponsor will notify the undue delay caused by temporary halt as well as the reason for such an action. The study will be suspended pending further review by the EC. The investigator should ensure that all subjects are kept informed.

9.2. AEs, SAEs and SUSARs

9.2.1. Adverse events (AEs)

Adverse events are defined as any undesirable experience occurring to a subject during the study, whether or not considered related to trial procedure. All adverse events reported spontaneously by the subject or observed by the investigator or his staff will be recorded.

9.2.2. Serious adverse events (SAEs)

A serious adverse event is any unfavourable medical occurrence or effect as follows

- Results in death;
- Life threatening (at the time of the event);
- Require inpatient hospitalization or prolongation of existing inpatients' hospitalization.
- congenital anomaly or birth defect;
- results in persistent or significant disability or incapacity;
- that required medical or surgical intervention to preclude of;

Any other important medical event that did not result in any of the outcomes listed above due to medical or surgical intervention but could have been based upon appropriate medical judgment.

An elective hospital admission will not be considered as a serious adverse event. Technical complications or vascular damage at the target lesion such as perforation or dissection that do not lead to clinically detectable SAE and neurological deterioration not caused by intracranial hemorrhage, new ischemic stroke, but are considered as consistent with the natural course of the ischemic stroke and its treatment, will not be reported immediately.

Serious adverse events will be immediately, after coming to notice of the investigator, reported to the site EC and sponsor.

The investigator will report the following SAEs occurring in the study period to the sponsor without undue delay of obtaining knowledge of the events: Death from any cause; symptomatic intracranial hemorrhage scored, extracranial hemorrhage, aspiration pneumonia, allergic contrast reactions, new ischemic stroke in different vascular territory.

SAEs of this study are reported using the "Suspicious Medical Device Adverse Event Report Form" (Table 9 in the appendix).

9.3. Follow-up of adverse events

All AEs will be followed until they have abated, or until a stable situation has been reached.

Depending on the event, follow up may require additional tests or medical procedures as indicated, and/or referral to the general physician or a medical specialist.

SAEs need to be reported till the end of the study in China, as defined in the protocol.

9.4. Data Safety Monitoring Board (DSMB)

In order to increase the safety of the intervention, the trial will be monitored by an independent DSMB. The DSMB will be chaired by a neurologist, and include a neuro-interventionist and an independent methodologist/statistician. The DSMB plans to conduct two interim analyses to evaluate the treatment effect and the incidence of adverse reactions according to the procedure at the end of the 90-day follow-up of 1/3 and 2/3 subjects, respectively. During the period of patient enrollment into the study, interim analyses of mortality and of any other information that is available on major outcomes (including serious adverse events believed to be due to treatment) will be supplied, in strict confidence, to the chairman of the DSMB, along with any other analyses that the DSMB may request. In the light of these analyses, DSMB will advise the chairman of the Steering Committee if, in their view, the randomized comparisons in DIRECT-MT have provided both (i) "proof beyond reasonable doubt" that for all, or for some specific types of patients, one particular treatment is clearly indicated or clearly contraindicated in terms of a net difference in outcome, and (ii) evidence that might reasonably be expected to materially influence patient management. Appropriate criteria of proof beyond reasonable doubt cannot be specified precisely, but a difference of at least 3 standard deviations in an interim analysis of a major outcome may be needed to justify halting, or modifying, the study prematurely. This criterion has the practical advantage that the number of interim analyses is of little importance.

The advice(s) of the DSMB will be sent to the sponsor of the study by the chair of the steering committee. Should the sponsor decide not to fully implement the advice of the DSMB, the sponsor will send the advice to the EC, including a note to substantiate why (part of) the advice of the DSMB will not be followed.

10. Statistical analysis

10.1. Statistical analysis

The primary effect parameter will be the common odds ratio, estimated with ordinal logistic regression, which represents the shift on the 7-category mRS scale measured at 3 months. The

estimate will be adjusted for the known prognostic variables age, pre-stroke mRS, time from onset to randomization, stroke severity (NIHSS) and collaterals and adjusted and unadjusted estimates with corresponding 95% confidence intervals will be reported. To explore for non-inferiority, we will analyze whether the lower bound of the 95% CI crossed 0.8, our pre-specified non-inferiority margin.

If applicable, the secondary outcomes will be analyzed using linear, logistic, or ordered regression analysis method, with the same correction method as the primary outcomes.

All analyses will be performed according to the intention-to-treat principle. Baseline data by treatment allocation will be reported with statistical procedures. Missing values for baseline characteristics will be reported. Missing baseline characteristics will be imputed using regression imputation. Pre-defined subgroups will be analyzed by testing for interaction between the specific baseline characteristic and treatment.

10.2. Subgroup analysis

The effect of intervention on the modified Rankin Scale will be analyzed in subgroups determined by the following variables:

- Tertiles of time from onset of symptoms to randomization, groin puncture and revascularization
- Ipsilateral extracranial carotid tandem lesion
- Occlusion location
- Collateral grades 0 to 3 as defined by Tan et al. [43] (Table 7 in Appendix)
- Thrombus characteristics (thrombus perviousness [44], clot burden, density)
- Large vessel occlusion due to different etiologies

10.3. Interim analysis

See Paragraph 9.4.

11. Ethical considerations

11.1. Regulation statement

The study will be conducted according to the principles of the Declaration of Helsinki (October 2013) [45]

11.2. Recruitment and consent

Following Article 21 of "Standard for quality management of medical device clinical trials" (June 1, 2016)[3], the investigators should adequately explain the details of the clinical trial, including known,

[3] http://www.sda.gov.cn/WS01/CL1101/148101.html

foreseeable risks and possible adverse event, etc., to the subject or to the guardians of subjects without capacity for civil conduct or with limited capacity for civil conduct. After full and detailed explanation, the subjects or their guardians sign the name and date in the informed consent form, and the investigators also need to sign the name and date in the informed consent form.

11.3. Problems of minors or incapacitated subjects

Minors (under 18 years old) will not be included in this trial. In the trial, about 50% of patients have language defects due to stroke, and about a quarter of the patients may suffer from a certain degree of lack of sense of disease. In such case, following the first paragraph of Article 23 of "Standard for quality management of medical device clinical trials" (June 1, 2016), for incapacitated subjects, if the ethics committee agrees in principle, and investigators believe that subjects participating in clinical trials are in their own interest, they can also enter the clinical trial, but their guardians should sign the name and date before the trial.

11.4. Benefits and risks assessment, group relatedness

The expected benefit from direct intra-arterial treatment compared to IVT followed by IAT may amount to 4% absolute increase in independent living at 3 months. Patients who have been allocated to the control group will be given usual treatment according to international, national and local guidelines. This includes treatment with IVT, followed by IAT.

11.5. Compensation for injury

Each participating center has purchased liability insurance. This insurance provides cover for damage to research subjects through injury or death caused by the study.

12. Administrative aspects, monitoring and publication

12.1. Handling and storage of data and documents

All data will be entered into a web-based database (EDC) by local research personnel. Subject records are coded by a unique study number. The local investigators will keep a list showing codes and names. Unique documents with identifying information will be stored separately from the study database in digital files, categorized by study number on a secure drive system, only accessible to the study coordinator.

12.2. Monitoring and quality assurance

The monitors will arrange visits according to the speed of enrollment of each center and the deviations found in the past. In principle, the inspection visit will be arranged within 5 working days of the center enrollment. The monitor will validate informed consent and source data for all subjects. The monitoring data including but not limited to: in-patient medical records, outpatient medical records, follow-up medical records, imaging materials and evaluation forms, etc. At the same time, the monitor will check the integrity and consistency of EDC data entry.

12.3. Amendment

Amendments are changes made to the research protocol after a favorable opinion by EC has been given. All amendments will be notified to the EC that gave a favorable opinion.

12.4. Annual progress report

The sponsor/investigator will submit a summary of the progress of the trial to the EC once a year. Information should be provided: the date of inclusion of the first subject, numbers of subjects included and numbers of subjects that have completed the trial, serious adverse events/ serious adverse reactions, other problems and amendments.

12.5. Temporary halt and (prematurely) end of study report

The investigator/sponsor will notify the EC of the end of the study within a period of 8 weeks. The end of the study is defined as the last patient's last visit.

The sponsor will notify the EC immediately of a temporary halt of the study, including the reason of such an action.

In case the study is ended prematurely, the sponsor will notify the EC within 15 days, including the reasons for the premature termination.

Within one year after the end of the study, the investigator/sponsor will submit a final study report with the results of the study, including any publications/abstracts of the study, to the EC and the Competent Authority.

The insurance applies to the damage that becomes apparent during the study or within 4 years after the end of the study.

12.6. Public disclosure and publication policy

The trial has been registered in clinicaltrials.gov. Clinicaltrials: NCT03469206

The study database will be closed within one month after the last scheduled follow-up date of the last included patient. A manuscript which at least describes the study and the answer to the primary research question will be submitted to a major clinical journal within 3 months from closure of the database. The manuscript will be shared with the financial sponsor(s) one month before submission, but the financial sponsor(s) will have no influence on its contents.

Anonymous data can be requested from the PI with a detailed description containing the aims and methods of the study for which the data are intended to be used. Data will be made available for this purpose at least 18 months after publication of the main report. Data may also be shared with non-commercial parties for scientific purposes, including individual patient meta-analyses, and with commercial parties for regulatory purposes.

These purposes should be specified in the informed consent form.

13. References

1. Wang W, Jiang B, Sun H, et al. Prevalence, Incidence, and Mortality of Stroke in China: Results from a Nationwide Population-Based Survey of 480 687 Adults. Circulation. 2017;135(8):759-71.

2. Wang C, Li SW, Wu SW, et al. Epidemiological survey of neurological disorders in six urban areas of the People's Republic of China. Zhong-Hua-Shen-Jing-Wai-Ke-Za-Zhi. 1985;1:2-7.

3. Feigin VL, Lawes CM, Bennett DA, et al. Worldwide stroke incidence and early case fatality reported in 56 population-based studies: a systematic review. Lancet Neurol. 2009;8(4):355-69.

4. Thrift AG, Thayabaranathan T, Howard G, et al. Global stroke statistics. Int J Stroke. 2017;12(1):13-32.

5. Kochanek KD, Murphy SL, Xu J, et al. Deaths: Final Data for 2014. Natl Vital Stat Rep. 2016;65(4):1-122.

6. Goyal M, Demchuk AM, Menon BK, Eesa M, Rempel JL, Thornton J, et al. Randomized assessment of rapid endovascular treatment of ischemic stroke. N Engl J Med. 2015;372(11):1019-30.

7. Jovin TG, Chamorro A, Cobo E, de Miquel MA, Molina CA, Rovira A, et al. Thrombectomy within 8 hours after symptom onset in ischemic stroke. N Engl J Med. 2015;372(24):2296-306.

8. Saver JL, Goyal M, Bonafe A, Diener HC, Levy EI,Pereira VM, et al. Stent-retriever thrombectomy after intravenous t-PA vs. t-PA alone in stroke. N Engl J Med. 2015;372(24):2285-95.

9. Brkhemer OA, Fransen PS, Beumer D, van den BergLA, Lingsma HF, Yoo AJ, et al. A randomized trial of intraarterial treatment for acute ischemic stroke. N Engl J Med. 2015;372(1):11-20.

10. Cmpbell BC, Mitchell PJ, Kleinig TJ, Dewey HM, Churilov L, Yassi N, et al.

Endovascular therapy for ischemic stroke with perfusion-imaging selection. N Engl J Med. 2015;372(11):1009-18.

11. Emberson J, Lees KR, Lyden P, Blackwell L, Albers G, Bluhmki E, et al. Effect of treatment delay, age, and stroke severity on the effects of intravenous thrombolysis with alteplase for acute ischaemic stroke: a meta-analysis of individual patient data from randomised trials. The Lancet. 2014;384(9958):1929-35.

12. Wardlaw JM, Murray V, Berge E, del Zoppo G, Sandercock P, Lindley RL, et al. Recombinant tissue plasminogen activator for acute ischaemic stroke: an updated systematic review and meta-analysis. Lancet. 2012;379(9834):2364-72.

13. Group TNt-PSS. Intracerebral Hemorrhage After Intravenous t-PA Therapy for Ischemic Stroke. Stroke. 1997;28(11):2109-18.

14. Mazya M, Egido JA, Ford GA, Lees KR, Mikulik R,Toni D, et al. Predicting the risk of symptomatic intracerebral hemorrhage in ischemic stroke treated with intravenous alteplase: safe Implementation of Treatments in Stroke (SITS) symptomatic intracerebral hemorrhage risk score. Stroke. 2012;43(6):1524-31.

15. ACTUAL Investigators. Direct endovascular treatment: an alternative for bridging therapy in anterior circulation large-vessel occlusion stroke. Eur J Neurol. 2017 Jul;24(7):935-943.

16. Berkhemer OA, Fransen PS, Beumer D, et al. A randomized trial of intraarterial treatment for acute ischemic stroke. N Engl J Med 2015; 372: 11–20.

17. Campbell BC, Mitchell PJ, Kleinig TJ, et al. Endovascular therapy for ischemic stroke with perfusion-imaging selection. N Engl J Med 2015; 372: 1009–1018.

18. Goyal M, Demchuk AM, Menon BK, et al. Randomized assessment of rapid endovascular treatment of ischemic stroke. N Engl J Med 2015; 372: 1019–1030.

19. Saver JL, Goyal M, Bonafe A, et al. Stent-retriever thrombectomy after intravenous t-PA vs. t-PA alone in stroke. N Engl J Med 2015; 372: 2285–2295.

20. Jovin TG, Chamorro A, Cobo E, et al. Thrombectomy within 8 hours after symptom onset in ischemic stroke. N Engl J Med 2015; 372: 2296–2306.

21. Arenillas JF. Intracranial atherosclerosis: current concepts. Stroke, 2011,42:S20-23.

22. Holmstedt CA, Turan TN, Chimowitz MI. Atherosclerotic intracranial arterial stenosis: risk factors, diagnosis, and treatment. Lancet Neurol, 2013,12:1106-1114.

23. Wityk RJ, Lehman D, Klag M, Coresh J, Ahn H, Litt B. Race and sex differences in the distribution of cerebral atherosclerosis. Stroke, 1996,27:1974-1980.

24. Sacco RL, Kargman DE, Gu Q, Zamanillo MC. Race-ethnicity and determinants of intracranial atherosclerotic cerebral infarction. The Northern Manhattan Stroke Study. Stroke, 1995,26:14-20.

25. Huang YN, Gao S, Huang Y, et al. Vascular lesion in Chinese patients with transient ischemic attacks. Neurology, 1997, 48(2):524-525.

26. Chandra RV, Leslie-Mazwi TM, Mehta BP, Derdeyn CP, Demchuk AM, Menon BK, et al. Does the use of IV tPA in the current era of rapid and predictable recanalization by mechanical embolectomy represent good value? Journal of NeuroInterventional Surgery. 2016;8(5):443-6.

27. Powers WJ, Derdeyn CP, Biller J, Coffey CS, HohBL, Jauch EC, et al. 2015 American Heart Association/American Stroke Association Focused Update of the 2013 Guidelines for the Early Management of Patients With Acute Ischemic Stroke Regarding Endovascular Treatment: A Guideline for Healthcare Professionals From the American Heart Association/American Stroke Association. Stroke. 2015;46(10):3020-35.

28. Hernandez AV, Steyerberg EW, Butcher I, Mushkudiani N, Taylor GS, Murray GD, et al. Adjustment for strong predictors of outcome in traumatic brain injury trials: 25% reduction in sample size requirements in the IMPACT study. J Neurotrauma. 2006;23(9):1295-303.

29. Lingsma H, Roozenbeek B, Steyerberg E, investigators I. Covariate adjustment increases statistical power in randomized controlled trials. J Clin Epidemiol. 2010;63(12):1391; author reply 2-3.

30. Wahlgren N, Ahmed N, Davalos A, Ford GA, Grond M, Hacke W, et al. Thrombolysis with alteplase for acute ischaemic stroke in the Safe Implementation of Thrombolysis in Stroke-Monitoring Study (SITS-MOST): an observational study. Lancet. 2007;369(9558):275-82.

31. Hacke W, Kaste M, Bluhmki E, Brozman M, DávalosA, Guidetti D, et al. Thrombolysis with Alteplase 3 to 4.5 Hours after Acute Ischemic Stroke. The New England Journal of Medicine. 2008;359(13):1317-29.

32. Campbell BC, Donnan GA, Lees KR, Hacke W, Khatri P, Hill MD, et al. Endovascular stent thrombectomy: the new standard of care for large vessel ischaemic stroke. Lancet Neurol. 2015;14(8):846-54.

33. Bracard S, Ducrocq X, Mas JL, Soudant M, Oppenheim C, Moulin T, et al. Mechanical thrombectomy after intravenous alteplaseversus alteplase alone after stroke (THRACE): a randomised controlled trial. Lancet Neurol. 2016;15(11):1138-47.

34. Muir KW, Ford GA, Messow CM, Ford I, Murray A, Clifton A, et al. Endovascular therapy for acute ischaemic stroke: the Pragmatic Ischaemic Stroke Thrombectomy Evaluation (PISTE) randomised, controlled trial. J Neurol Neurosurg Psychiatry.

2017;88(1):38-44.

35. van Swieten JC, Koudstaal PJ, Visser MC, Schouten HJ, van Gijn J. Interobserver agreement for the assessment of handicap in stroke patients. Stroke. 1988;19(5):604-7.

36. Goyal M, Fargen KM, Turk AS, Mocco J, Liebeskind DS, Frei D, et al. 2C or not 2C: defining an improved revascularization grading scale and the need for standardization of angiography outcomes in stroke trials. J Neurointerv Surg. 2014;6(2):83-6.

37. Brott T, Adams HP, Olinger CP, Marler JR, Barsan WG, Spilker J, et al. Measurements of acute cerebral ifarction: a clinical examination scale Stroke. 1989;20:864-70.

38. Boers AM, Marquering HA, Jochem JJ, Besselink NJ, Berkhemer OA, van der Lugt A, et al. Automated cerebral infarct volume measurement in follow-up noncontrast CT scans of patients with acute ischemic stroke. AJNR Am J Neuroradiol. 2013;34(8):1522-7.

39. Group E. EuroQol--a new facility for the measurement of health-related quality of life. Health Policy. 1990;16(3):199-208.

40. Mahoney FI, Barthel DW. Functional Evaluation: The Barthel Index. Md State Med J. 1965;14:61-5.

41. von Kummer R, Broderick JP, Campbell BC, Demchuk A, Goyal M, Hill MD, et al. The Heidelberg Bleeding Classification: Classification of Bleeding Events After Ischemic Stroke and Reperfusion Therapy. Stroke. 2015;46(10):2981-6.

42. Goyal M, Menon BK, Demchuk A, Saver JL, Eesa M,Majoie C, et al. Proposed methodology and classification of Infarct in New Territory (INT) after endovascular stroke treatment. J Neurointerv Surg. 2017;9(5):449-50.

43. Tan IY, Demchuk AM, Hopyan J, Zhang L, Gladstone D, Wong K, et al. CT angiography clot burden score and collateral score:correlation with clinical and radiologic outcomes in acute middle cerebral artery infarct. AJNR Am J Neuroradiol. 2009;30(3):525-31.

44. Santos EM, Marquering HA, den Blanken MD, Berkhemer OA, Boers AM, Yoo AJ, et al. Thrombus Permeability Is Associated With Improved Functional Outcome and Recanalization in Patients With Ischemic Stroke. Stroke. 2016;47(3):732-41.

45. World Medical A. World Medical Association Declaration of Helsinki: ethical principles for medical research involving human subjects. JAMA. 2013;310(20):2191-4.

46. Puetz V, Dzialowski I, Hill MD, Subramaniam S, Sylaja PN, Krol A, et al. Intracranial thrombus extent predicts clinical outcome, final infarct size and hemorrhagic transformation in ischemic stroke: the clot burden score. Int J Stroke. 2008;3(4):230-6.

14. Table

Table 1 Modified Rankin Scale [35]

The modified Rankin Scale (mRS) is an ordinal hierarchical scale ranging from 0 to 5, with higher scores indicating more severe disability. A score of 6 has been added to signify death.

Category	Short description	Long description
0	No symptoms	No symptoms
1	Symptoms, no disability	Minor symptoms that do not interfere with lifestyle
2	Slight disability	Slight disability, symptoms that lead to some restriction in lifestyle, but do not interfere with the patient's capacity to look after himself.
3	Moderate disability	Moderate disability, symptoms that significantly restrict lifestyle and prevent totally independent existence
4	Moderately severe disability	Moderately severe disability, symptoms that clearly prevent independent existence though not needing constant attention
5	Severe disability	Severe disability, totally dependent patient requiring constant attention day and night.
6	Death	Death

Table 2 Extended Treatment In Cerebral Ischemia (Etici) Scale [36]

eTICI grade	Short description	Long description
**0**	No perfusion	No antegrade flow beyond the point of occlusion
**1**	Limited reperfusion	Antegrade reperfusion past the initial occlusion, but limited distal branch filling with little or slow distal reperfusion
**2a**	<50% reperfusion	Antegrade reperfusion of less than half of the occluded target artery previously ischemic territory (eg, in 1 major division of the MCA and its territory)
**2b**	≥50% and <90% reperfusion	Antegrade reperfusion of more than half of the previously occluded target artery ischemic territory (eg, in 2 major divisions of the MCA and its territories)
**2c**	≥90% reperfusion	Near complete antegrade reperfusion of the previously occluded target artery ischemic territory, except for slow flow or distal emboli in a few distal cortical vessels
**3**	100% reperfusion	Complete antegrade reperfusion of the previously occluded target artery ischemic territory, with absence of visualized occlusion in all distal branches

MCA: middle cerebral artery; eTICI; extended treatment in cerebral ischemia scale

Table 3 NIH Stroke Scale

The NIHSS is an ordinal hierarchical scale to evaluate the severity of stroke by assessing a patient's performance. [23] Scores range from 0 to 42, with higher scores indicating a more severe deficit. Administer stroke scale items in the order listed. Record performance in each category after each subscale exam. Do not go back and change scores. Follow directions provided for each exam technique. Scores should reflect what the patient does, not what the clinician thinks the patient can do. The clinician should record answers while administering the exam and work quickly. Except where indicated, the patient should not be coached (i.e. repeated requests to patient to make a special effort).

Instructions	Scale definition
**1a. Level of consciousness.** The investigator must choose a response if a full evaluation is prevented by such obstacles as an endotracheal tube, language barrier, orotracheal trauma/bandages. A 3 is scored only if the patient makes no movement (other than reflexive posturing) in response to noxious stimulation.	0 = **Alert;** keenly responsive.<br>1 = **Not alert;** but arousable by minor stimulation to obey, answer, or respond.<br>2 = **Not alert;** required repeated stimulation to attend, or is obtunded and requires strong or painful stimulation to make movements (not stereotyped).<br>3 = Responds only with reflex motor or autonomic effects or totally unresponsive, flaccid and areflexic.
**1b. LOC Questions:** The patient is asked the month and his/her age. The answer must be correct – there is not partial credit for being close. Phasic and stuporous patients who do not comprehend the questions will score 2. Patients unable to speak because of endotracheal intubation, orotracheal trauma, severe dysarthria from any cause, language barrier, or any other problem not secondary to aphasia are given a 1. It is important that only the initial answer be graded and that the examiners not "help" the patient with verbal or non-verbal clues.	0 = Answers both questions correctly.<br>1 = Answers one question correctly.<br>2 = Answers neither question correctly.
**1c. LOC Commands:** The patient is asked to open and close the eyes and then to grip and release the non-paretic hand. Substitute another one step command if the hand cannot be used. Credit is given if an unequivocal attempt is made but not completed due to weakness. If the patient does not respond to command, the task should be demonstrated to him or her (pantomime), and the result scored<br>(i.e. follows none, one or two commands). Patients with trauma, amputation, or other physical impediments should be given suitable one-step commands. Only the first attempt is scored.	0 = Performs both tasks correctly.<br>1 = Performs one task correctly.<br>2 = Performs neither task correctly.
**2. Best Gaze:** Only horizontal eye movements will be tested. Voluntary or reflexive (oculocephalic) eye movements will be scored,	**0= Normal.**<br>1= **Partial gaze palsy;** gaze is abnormal in one or both eyes, but forced deviation or total gaze paresis is not

but caloric testing is not done. If the patient has a conjugate deviation of the eyes that can be overcome by voluntary or reflexive activity, the score will be a 1. If a patient has an isolated peripheral nerve paresis (CN III, IV or VI), score a 1. Gaze is testable in all aphasic patients. Patients with ocular trauma, bandages, preexisting blindness, or other disorder of visual acuity or fields should be tested with reflexive movements, and a choice made by the investigator. Establishing eye contact and then moving about the patient from side to side will occasionally clarify the presence of a partial gaze palsy.	present.<br>2= **Forced deviation;** or total gaze paresis not overcome by the oculocephalic maneuver.
**3. Visual:** Visual fields (upper and lower quadrants) are tested by confrontation, using finger counting or visual threat, as appropriate. Patients may be encouraged, but if they look at the side of the moving finger appropriately, this can be scored as normal. If there is unilateral blindness or enucleation, visual fields in the remaining eye are scored. Score 1 only if a clear-cut asymmetry, including quadrantanopia, is found. If patient is blind from any cause, score 3.<br>Double simultaneous stimulation is performed in this case.<br>If there is extinction, the patient receives a 1, and the results are used to respond to item 11.	**0= No visual loss.**<br>**1= Partial hemianopia.**<br>**2= Complete hemianopia.**<br>3= **Bilateral hemianopia**<br>(blind including cortical blindness)
**4. Facial palsy:** Ask or use pantomime to encourage the patient to show teeth or raise eyebrows and close eyes. Score symmetry of grimace in response to noxious stimuli in the poorly response or non-comprehending patient. If facial trauma/bandages, orotracheal tube, tape or other physical barriers obscure the face, these should be removed to the extent possible.	**0 = Normal symmetrical movements.**<br>1= **Minor paralysis**<br>(flattened nasolabial fold, asymmetry on smiling)<br>2= **Partial paralysis** (total or near-total paralysis of lower face)<br>3= **Complete paralysis of one or both sides** (absence of facial movement in the upper and lower face).
**5. Motor arm:** The limb is placed in the appropriate position: extend the arms (palms down) 90 degrees (if sitting) or 45 degrees (if supine). Drift is scored if the arm falls before 10 seconds. The aphasic patient is encouraged using urgency in the voice and pantomime, but not noxious stimulation. Each limb is tested in turn, beginning with the non-paretic arm. Only in the case of amputation or joint fusion at the shoulder, the examiner should record the score as untestable (UN), and clearly write the explanation for this choice.	0= **No drift**; limb holds 90 (or 45) degrees for full 10 seconds.<br>1= **Drift**; limb holds 90 (or 45) degrees, but drifts down before full 10 seconds; does not hit bed or other support.<br>2= **Some effort against gravity;** limb cannot get to or maintain (if cued) 90 (or 45) degrees, drifts down to bed, but has some effort against gravity.<br>3= **No effort against gravity;** limb falls.<br>**4= No movement.**<br>UN = Amputation or joint fusion: explain:<br>**5a = Left Arm.**<br>**5b = Right arm.**
**6. Motor leg:** The limb is placed in the appropriate position: hold the leg at 30 degrees	0= **No drift;** leg holds 30-degree position for full 5 seconds.

(always tested supine). Drift is scored if the leg falls before 5 seconds. The aphasic patient is encouraged using urgency in the voice and pantomime, but not noxious stimulation. Each limb is tested in turn, beginning with the non-paretic leg. Only in the case of amputation or joint fusion at the hip, the examiner should record the score as untestable (UN), and clearly write the explanation for this choice.	1= **Drift;** leg falls by the end of the 5-second period but does not hit bed.<br>2= **Some effort against gravity;** leg falls to bed by 5 seconds, but has some effort against gravity.<br>3= **No effort against gravity;** leg falls to bed immediately.<br>**4= No movement.**<br>UN = Amputation or joint fusion: explain:<br>**6a. Left Leg**<br>**6b. Right Leg.**
**7. Limb ataxia:** This item is aimed at finding evidence of a unilateral cerebellar lesion. Test with eyes open. In case of visual defect, ensure testing is done in intact visual field. The finger-nose-finger and heel-shin tests are performed on both sides, and ataxia is scored only if present out of proportion to weakness. Ataxia is absent in the patient who cannot understand or is paralyzed. Only in the case of amputation or joint fusion, the examiner should record the score as untestable (UN), and clearly write the explanation for this choice. In case of blindness, test by having the patient touch nose from extended arm position.	0= Absent.<br>1**= Present in one limb.**<br>2**= Present in two limbs.**<br>UN = Amputation or joint fusion: explain:
**8. Sensory:** Sensation or grimace to pinprick when tested, or withdrawal from noxious stimulus in the obtunded or aphasic patient. Only sensory loss attributed to stroke is scored as abnormal and the examiner should test as many body areas (arms [not hands], legs, trunk, face) as needed to accurately check for hemisensory loss. A score of 2, 'severe or total sensory loss', should only be given when a severe or total loss of sensation can be clearly demonstrated. Stuporous and aphasic patients will, therefore, probably score 1 or 0. The patient with brainstem stroke who has bilateral loss of sensation is scored 2. If the patient does not respond and is quadriplegic, score 2. Patients in a coma (item 1a=3) are automatically given a 2 on this item.	0= **Normal;** no sensory loss.<br>1= **Mild-to-moderate sensory loss;** patients feels pinprick is less sharp or is dull on the affected side; or there is a loss of superficial pain with pinprick, but patient is aware of being touched.<br>2= **Severe to total sensory loss;** patient is not aware of being touched in the face, arm and leg.
**9. Best language:** A great deal of information about comprehension will be obtained during the preceding sections of the examination. For this scale item, the patient is asked to describe what is happening in the attached picture, to name the items on the attached naming sheet and to read from the attached list of sentences. Comprehension is judged from responses here, as well as to all of the commands in the preceding general neurological exam. If visual loss interferes with the tests, ask the patient to identify objects placed in the hand, repeat, and	0= **No aphasia;** normal<br>1= **Mild-to-moderate aphasia;** some obvious loss of fluency or facility of comprehension, without significant limitation on ideas expressed or form of expression. Reduction of speech and/or comprehension, however, makes conservation about provided materials difficult or impossible. For example, in conversation about provided materials, examiner can identify picture or naming card content from patient's response.<br>2= **Severe aphasia;** all communication is through fragmentary expression; great need for inference,

produce speech. The intubated patient should be asked to write. The patient in a coma (item 1a=3) will automatically score 3 on this item. The examiner must choose a score for the patient with stupor or limited cooperation, but a score of 3 should be used only if the patient is mute and follows no one-step commands.	questioning, and guessing by the listener. Range of information that can be exchanged is limited; listener carries burden of communication. Examiner cannot identify materials provided from patient response.<br>3 = **Mute, global aphasia:** no usable speech or auditory comprehension.
**10. Dysarthria:** If patient is thought to be normal, an adequate sample of speech must be obtained by asking patient to read or repeat words from the attached list. If the patient has severe aphasia, the clarity of articulation of spontaneous speech can be rated. Only if patient is intubated or has other physical barriers to producing speech, the examiner should record the score as untestable (UN), and clearly write an explanation for this choice. Do not tell the patient why he or she is being tested.	**0= Normal.**<br>1= **Mild-to-moderate dysarthria;** patient slurs at least some words and, at worst, can be understood by some difficulty.<br>2= **Severe dysarthria:** patient's speech is so slurred as to be unintelligible in the absence of or out of proportion to any dysphasia, or is mute/anarthric.<br>UN = Intubated or other physical barrier.
**11. Extinction and Inattention** (formerly Neglect): Sufficient information to identify neglect may be obtained during the prior testing. If the patient has a severe visual loss preventing visual double simultaneous stimulation, and the cutaneous stimuli are normal, the score is normal. If the patient has aphasia but does appear to attend to both sides, the score is normal. The presence of visual spatial neglect or anosagnosia may also be taken as evidence of abnormality. Since the abnormality is scored only if present, the item is never untestable.	**0= No abnormality.**<br>1= **Visual, tactile, auditory, spatial, or personal inattention** or extinction to bilateral simultaneous stimulation in one of the sensory modalities.<br>2= **Profound hemi-inattention or extinction to more than one modality;** does not recognize own hand or orients to only one side of space.

Table 4 Barthel Index [40]

The Barthel index (BI) is an ordinal scale used to measure performance in 10 activities of daily living (ADL). Test scores range from 0 to 100, with higher scores indicating better performance in these activities.

Category	Scale definition
Feeding	0 = unable<br>5 = needs help cutting, spreading butter, etc., or requires modified diet<br>10 = independent
Bathing	0 = dependent<br>5 = independent (or in shower)
Grooming	0 = needs to help with personal care<br>5 = independent face/hair/teeth/shaving (implements provided)
Dressing	0 = dependent<br>5 = needs help but can do about half unaided<br>10 = independent (including buttons, zips, laces, etc.)
Bowels	0 = incontinent (or needs to be given enemas)<br>5 = occasional accident<br>10 = continent
Bladder	0 = incontinent, or catheterized and unable to manage alone<br>5 = occasional accident<br>10 = continent
Toilet use	0 = dependent<br>5 = needs some help, but can do something alone<br>10 = independent (on and off, dressing, wiping)
Transfers (bed to chair and back)	0 = unable, no sitting balance<br>5 = major help (one or two people, physical), can sit<br>10 = minor help (verbal or physical)<br>15 = independent
Mobility (on level surfaces)	0 = immobile or < 50 yards<br>5 = wheelchair independent, including corners, > 50 yards<br>10 = walks with help of one person (verbal or physical) > 50 yards<br>15 = independent (but may use any aid; for example, stick) > 50 yards
Stairs	0 = unable<br>5 = needs help (verbal, physical, carrying aid)<br>10 = independent

Guidelines

8. The index should be used as a record of what a patient does, not as a record of what a patient could do.
9. The main aim is to establish degree of independence from any help, physical or verbal, however minor and for whatever reason.
10. The need for supervision renders the patient not independent.
11. A patient's performance should be established using the best available evidence. Asking the patient, friends/relatives and nurses are the usual sources, but direct observation and common sense are also important. However direct testing is not needed.
12. Usually the patient's performance over the preceding 24-48 hours is important, but occasionally longer periods will be relevant.
13. Middle categories imply that the patient supplies over 50 per cent of the effort.
14. Use of aids to be independent is allowed.

Table 5 EUROQOL 5D-5L [39]

The EuroQoL 5-dimensions 5-level (EQ-5D-5L) questionnaire is a standardized measure of health outcome that has been used extensively in patients with stroke.

Under each heading, please tick the ONE box that best describes your health TODAY.

Mobility

I have no problems in walking about ☐

I have slight problems in walking about ☐

I have moderate problems in walking about ☐

I have severe problems in walking about ☐

I am unable to walk about ☐

Self-care

I have no problems washing or dressing myself ☐

I have slight problems washing or dressing myself ☐

I have moderate problems washing or dressing myself ☐

I have severe problems washing or dressing myself ☐

I am unable to wash or dress myself ☐

Usual activities (e.g. work, study, housework, family or leisure activities)

I have no problems doing my usual activities ☐

I have slight problems doing my usual activities ☐

I have moderate problems doing my usual activities ☐

I have severe problems doing my usual activities ☐

I am unable to do my usual activities ☐

Pain/discomfort

I have no pain or discomfort ☐

I have slight pain or discomfort ☐

I have moderate pain or discomfort ☐

I have severe pain or discomfort ☐

I have extreme pain or discomfort ☐

Anxiety/depression

I am not anxious or depressed ☐

I am slightly anxious or depressed ☐

I am moderately anxious or depressed ☐

I am severely anxious or depressed ☐

I am extremely anxious or depressed ☐

Table 6 Clot Burden Score for CTA and MRA [46]

No contrast agent filling	Score
Supraclinoid internal carotid artery	2
Proximal M1	2
Distal M1	2
Infraclinoid internal carotid artery	1
A1 branch	1
M2 branch	1
**Total score: 10 – Sum**	**Total**

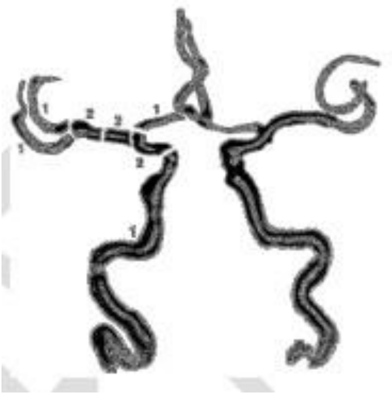

Table 7 Collateral Score [43]

Category	Score	Description
**None**	0	Absent collaterals
**Poor**	1	Collaterals filling ≤50% of the occluded territory
**Intermediate**	2	Collaterals filing >50%, but <100% of the occluded territory
**Good**	3	Collaterals filling 100% of the occluded territory

Table 8 Classification pf Infarct in a New Territory [42]

<table>
<tr><th colspan="2">Classification based on size</th><th colspan="2">Classification based on catheter manipulation across territory ostium</th></tr>
<tr><td>**Type I**</td><td>≤2 mm diffusion lesion (unidentifiable on NCCT)</td><td rowspan="2">**Type A**</td><td rowspan="2">Catheter was manipulated past the ostium of the new territory (e.g. large ACA infarct in a patient with an initial M1 occlusion): greater likelihood that infarct is related to the procedure</td></tr>
<tr><td rowspan="2">**Type II**</td><td rowspan="2">>2 mm to ≤ 20 mm lesion (potentially difficult to identify on CT scan)</td></tr>
<tr><td rowspan="2">**Type B**</td><td rowspan="2">Catheter was not manipulated past the ostium of the new territory (e.g. left PICA infarct in a patient with an initial right M1 occlusion): lower likelihood that infarct is related to procedure</td></tr>
<tr><td>**Type III**</td><td>Large (> 20 mm) infarct</td></tr>
</table>

Glossary: NCCT: Non contrast computed tomography; CTA: Computed tomography angiogram; IAT: intra-arterial treatment; NIHSS: National Institutes of Health Stroke Scale.

Table 9 Report of Suspicious Medical Device Adverse Events

Report of Suspicious Medical Device Adverse Events

Report date: Code: ☐ ☐ ☐ ☐ ☐ ☐ ☐ ☐ ☐ ☐ ☐

Report source: ☐ Manufacturer ☐ Distributor ☐ User Unit name:

Contact address: Post code: Contact Tel.:

**C. Patient**			**C. Medical device**
1. Name	2. Age	3. Gender:<br>☐ Male ☐ Female	11. Product name:
4. Disease to be treated or expected effect:			12. Trade name:
**D. Overview of adverse event**			13. Registration No.:
5. Main conditions of the event:			14. Name of the manufacturer:<br>Address of the manufacturer:<br>Telephone of the manufacture:
6. Event occurrence date:<br>7. Time of discovery or knowledge:			15. Model/specification:<br>Product number:<br>Lot number:
8. Place where the medical device is actually used:<br>☐ Medical institution ☐ Home<br>☐ Others (please specify):			**15.** Operator:<br>☐ Professional ☐ Non-professional<br>☐ Patient ☐ Others (specific information):
9. Consequence<br>☐ Death_________________________(specific time);<br>☐ Life threatening;<br>☐ Permanent injury to the functional structure of the body;<br>☐ May lead to permanent injury to the functional structure of the body;<br>☐ Need internal and surgical treatment to avoid the above permanent injury;			17. Expiration date:<br>18. Production date:<br>19. Discontinuation date:<br>20. Implantation date (if implanted):<br>21. Preliminary cause analysis of the event:

☐ Others (details should be given in "Event description").	
10. Event description: (Including at least the device usage time, purpose of use, usage basis, usage situation, adverse event occurred, impact on the victim, treatment measures taken, and the joint use of devices)	22. Preliminary handling of the event:
	23. Reporting progress of the event<br>☐ User has been notified ☐ Manufacturer has been notified<br>☐ Distributor has been notified ☐ Pharmaceutical supervision department has been notified
	**D. Relevance evaluation**
	(4) Was there any reasonable chronological sequence between the using of medical device and occurred/possible injury event? Yes ☐ No ☐<br>(5) Did the occurred/possible injury event belong to the injury type that may be caused by the medical device used? Yes ☐ No ☐ Not clear ☐<br>(6) Could the occurred/possible injury event be explained by combining the effect of drug and/or device, patient's condition or other non-medical device factors?<br>Yes ☐ No ☐ Not clear ☐<br>Evaluation conclusion: Very likely ☐ Possible ☐ Doubtful □ Undeterminable ☐
	**E. AE assessment**
	24. Evaluation opinions of provincial monitoring technical site (attached pages are acceptable):
	25. Evaluation opinions of national monitoring technical site (attached pages are acceptable):

Reporter: Physician ☐ Technician ☐ Nurse ☐ Others ☐

Signature of reporter: **Prepared by China Food and Drug Administration**

17. Figure

Figure 1 DIRECT-MT Trial Logo

Figure 2 Patient Flow in the Trial

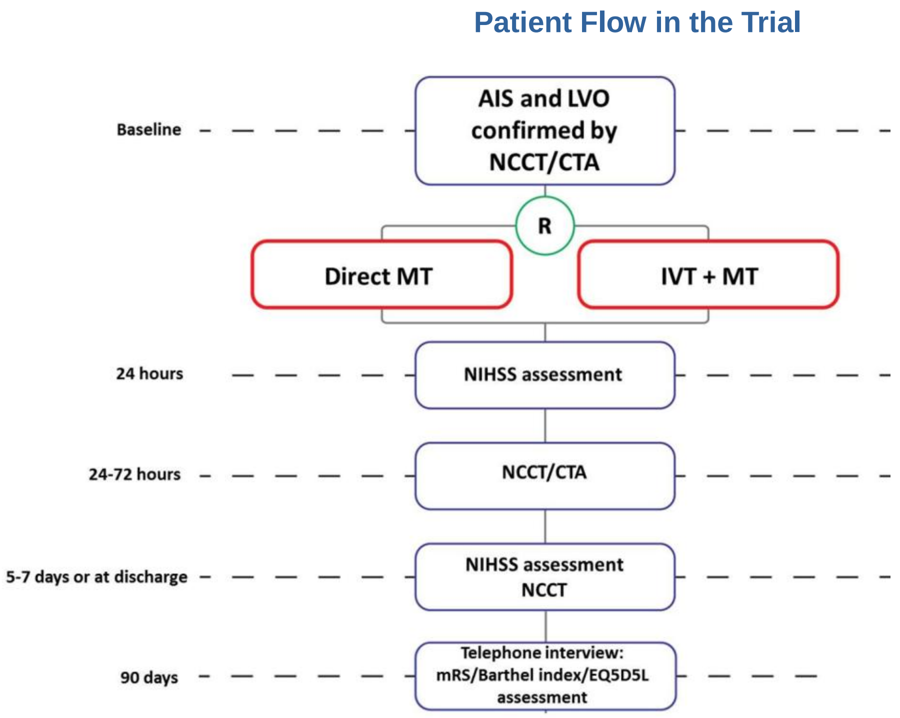

Glossary: CTA: Computed tomography angiogram; IAT: intra-arterial treatment; IVT: intravenous thrombolysis; NIHSS: National Institutes of Health Stroke Scale.

18. Appendix

16.1 Study committees

Steering Committee

Chairman: Prof Liu Jianmin, Changhai Hospital Affiliated to the Second Military Medical University
Members: Prof Deng Benqiang, Changhai Hospital Affiliated to the Second Military Medical University; Prof Charles Majoie, Academisch Medisch Centrum bij de Universitieit van Amsterdam (AMC); and Prof Yvo Roos, Academisch Medisch Centrum bij de Universitieit van Amsterdam (AMC)

Data Safety Monitoring Board

Chairman: Prof. Craig Anderson, The George Institute for Global Health at Peking University Health Science Center
Members: Prof. Miao Zhongrong, Beijing Tiantan Hospital Affiliated to Capital Medical University; Prof. He Jia, Department of Health Statistics of Second Military Medical University

Imaging Assessment Committee

Chairman: Prof. Jianping Lu, Department of Radiology, Changhai Hospital, Naval Medical University
Members:
Prof. Bing Tian, Department of Radiology, Changhai Hospital, Naval Medical University
Yongxin Zhang, Department of Neurosurgery, Changhai Hospital, Naval Medical University
Lei Zhang, Department of Neurosurgery, Changhai Hospital, Naval Medical University
Hao Wang, Department of Neurology, Linyi People's Hospital
Zhang Shi, Department of Radiology, Changhai Hospital, Naval Medical University
Wenjia Peng, Department of Radiology, Changhai Hospital, Naval Medical University
Xuefeng Zhang, Department of Radiology, Changhai Hospital, Naval Medical University
Xia Tian, Department of Radiology, Changhai Hospital, Naval Medical University
Tengfei Zhou, Department of Radiology, Henan Provincial People's Hospital
Xiaoquan Xu, Department of Radiology, Jiangsu Provincial People's Hospital
Shenqiang Yan, Department of Neurology, Second Affiliated Hospital; Zhejiang University College of Medicine
Jun Ke, Department of Radiology, First Affiliated Hospital, Soochow University
Guang Zhang, Department of Neurosurgery, First Affiliated Hospital, Harbin Medical University
Jun Shi, Core lab, Cardiovascular Chinese research center (CCRC)
Fang Li, Core lab, Cardiovascular Chinese research center (CCRC)
Xin Wang, Core lab, Cardiovascular Chinese research center (CCRC)

Adverse Event Adjudication Committee

Chairman: Prof. Fang Qi, The First Affiliated Hospital of Soochow University

Members: Prof. Zhang Yongwei, Changhai Hospital Affiliated to the Second Military Medical University; Prof. Fu Jianhui, Huashan Hospital Affiliated to Fudan University

Outcome Committee

Chairman: Prof. Li Yansheng, Renji Hospital of Shanghai Jiaotong University School of Medicine
Members: Prof. Zhang Ping, Changhai Hospital Affiliated to the Second Military Medical University; Prof. Zhang Yingying, Huadong Hospital Affiliated to Fudan University.

16.2 DIRECT-MT recommendations of the Steering Committee with regard to type of mechanical thrombectomy and use of thrombolytic agents during endovascular procedures.

General

Inclusion in the trial, randomization, and subsequent endovascular treatment with/without prior IVT should be started as soon as possible after presentation in all eligible patients.

The target time from study randomization to groin puncture will be as fast as possible. All patients would be better to undergo groin puncture within a median of 60 minutes after randomization.

Neuroimaging

Neuroimaging studies to assess vessel patency should be done before or simultaneously with treatment with intravenous (IV) alteplase, in order not to lose time and brain. We aim to not cause any delay prior to intra-arterial treatment, by infusion of IV alteplase.

Additional thrombolytic agents, dose and type

If deemed indicated by the interventionist, local application (intra-arterial) alteplase is allowed in any of the patients included in the DIRECT-MT.

Patients who have been pre-treated with IV alteplase should not receive more than 30 mg alteplase during intra-arterial treatment. The steering committee recommends that the alteplase is delivered in shots of 5 mg, in 5-10 minutes time intervals. In individual cases, an equivalent dose of 400,000 U urokinase, delivered in shots of 50.000 - 100.000 U, in 5-10 minutes time intervals, is also accepted as escape medication. Vessel patency should be checked after each shot.

Type of mechanical thrombectomy device(s)

All stent retriever and aspiration devices for IAT, which are approved for this purpose by CFDA, and have been approved for use in the study by the steering committee are allowed in the trial as a first line of defense and are listed below:

Device name	Manufacturer	Description
**Solitaire**	Medtronic / Covidien	Retrievable stent
**Trevo**	Stryker	Retrievable stent

**Revive**	Johnson & Johnson/ Cerenovus	Retrievable stent

A second device is allowed as a second option, when the first device has failed according to the interventionist. The further choice of the particular device for a certain patient is left to the discretion of the interventionist.

16.3 Imaging requirements

16.3.1 Minimum baseline imaging requirements

When

1) Before randomization, a NCCT and CTA should be performed to assess eligibility for the study.

How

4. Pre-randomization NCCT:

    3. The thickness of the NCCT scanning layer is recommended to be 5 mm, and 5-8 mm is also acceptable.

    4. The NCCT study should include the whole head.

5. Pre-randomization CTA:

    4. The CTA study should cover the whole area from the aortic arch to the vertex, and intracranial part only is also acceptable.

    5. The CTA study should include thin slices (maximum of 1.0 mm)

    6. The CTA study should include the following reconstructions

        iii. Axial maximum intensity projection (MIP),

            3. MIP slab thickness: 25 mm

            4. Overlap: 5 mm

        iv. Coronal MIP

            3. MIP slab thickness: 25 mm

            4. Overlap: 5 mm

6. After acquisition

    3. All images (both NCCT and CTA) should be saved to the DICOM format

    4. All available series should be sent to the core lab for assessment, including thin slice series (for thrombus assessment).

16.3.2 Intervention-related angiographic imaging

When

5) Before the intervention complete AP and Lateral angiograms (of whole head and including venous phase) should be performed to evaluate the site of vessel occlusion, extent of thrombus, territories involved, concomitant pathologies and to assess collateral flow.

6) After each passage of a mechanical or aspirational device, a control angiogram should be performed.

7) After each bolus of (a rescue) thrombolytic agent, a control angiogram should be performed.

8) At the end of the procedure complete AP and Lateral angiograms (of whole head and including venous phase) should be repeated. Without these complete runs, optimal TICI scoring is not possible

How

Pre-intervention and end-of-procedure angiogram:

m. Angiograms should be performed through the guiding catheter

n. Baseline and final AP views and lateral views of the intracranial arteries are mandatory. Both are required to assess reperfusion after the procedure.

o. Baseline and final angiograms should include both the arterial and venous phases of the injection to evaluate the collateral pathways and perfusion of the distal vascular bed.

p. Baseline and final angiograms should include the internal carotid artery feeding the target vessel as demonstrated on CTA.

q. Baseline and final angiograms should include the common carotid and internal carotid artery in case of occlusion, dissection or severe stenosis in the carotid feeding the target vessel as demonstrated on CTA.

r. Angiograms should be performed via the guiding catheter with the same catheter position and same views before and after the procedures to adequately assess the results of therapy.

After each device placement:

s. A non-contrast radiograph should be obtained

t. At least one view at the discretion of the interventionalist

After each passage of mechanical or aspirational device or bolus of (rescue) thrombolytic agent :

u. Angiograms should be performed through the guiding catheter

v. At least one view, at the discretion of the interventionalist.

After the procedure

w. Complete series of the angiograms and microcatheter injections (when performed)

should be saved according to the DICOM standard.

x. All series should be forwarded to the imaging assessment committee.

16.3.3 Minimum follow-up imaging requirements

When

4) 24-72 hours after undergoing endovascular treatment, a NCCT and CTA should be performed to assess treatment efficacy.

5) 5-7 days after undergoing endovascular treatment, or before discharge a NCCT should be performed to assess final lesion volume and potential hemorrhages complications.

6) If clinically required (i.e. in cases of clinical deterioration of the patient) additional imaging as needed, at the discretion of the treating physician is acquired.

How

24-72 hours NCCT:

11. The thickness of the NCCT scanning layer is recommended to be 5 mm, and 5-8 mm is also acceptable.
12. The NCCT study should include the whole head.

24-72 hours CTA:

13. The CTA study should cover the whole area from the aortic arch to the vertex, and intracranial part only is also acceptable.
14. The CTA study should include thin slices (maximum of 1.0 mm)
15. The CTA study should include the following reconstructions

    iii. Axial maximum intensity projection (MIP),

    3. MIP slab thickness: 25 mm
    4. Overlap: 5 mm

    iv. Coronal MIP

    3. MIP slab thickness: 25 mm
    4. Overlap: 5 mm

5-7 days NCCT (or before discharge)

16. The NCCT study should contain both thick (5mm) and thin slices (maximum of 2.5mm).
17. The NCCT study should include the whole head.

18. In addition, clinically required imaging is at the discretion of the treating physician.

19. After acquisition, all images (NCCT, CTA, and additional imaging) should be saved to the DICOM file format

20. All available series should be sent to the core lab for assessment, including thin slice series (for thrombus assessment).

Summary of protocol amendments（version 2.0）

	SECTION	Original version	Modified version	Explanation
1	Front cover	Version: 1.0，Date： December 1, 2017	Version: 2.0，Date： August 31, 2018	revised version
2	Summary: Rationale	we hypothesize that direct IAT may lead to **an 8% absolute increase** in good outcome compared to IAT preceded by IVT.	**The HERMES study showed that the incidence of symptomatic intracranial hemorrhage was about 4.4% in the western population. Considering the high rate of intracranial atherosclerosis in Chinese population, the clinical prognosis after thrombectomy may be slightly better.** Therefore, we hypothesize that direct IAT may lead to **a 4% absolute increase** in good outcome compared to IAT preceded by IVT.	Increase the results of the HERMES study and modify the expected value of good prognosis
3	Summary: Objective	To assess the effect of direct IAT compared to IVT followed by IAT, in patients with acute ischemic stroke, caused by a CTA-confirmed occlusion of the anterior circulation (**ICA-T/L**, M1, proximal M2) on functional outcome.	To assess the effect of direct IAT compared to IVT followed by IAT, in patients with acute ischemic stroke, caused by a CTA-confirmed occlusion of the anterior circulation (**intracranial segment of internal carotid artery**, M1, proximal M2) on functional outcome.	More detail
4	Summary: Main study parameters/outcomes	Secondary outcomes include mortality at 90 days, stroke severity at 24-72 hours and 5-7 days, recanalization on CTA at 24-72 hours, dichotomous clinical outcome on the mRS and	Secondary outcomes include mortality at 90 days, stroke severity at 24 ± 6 hours and 5-7 days, recanalization on CTA at 24-72 hours,	Revise the time window for NIHSS score

		infarct size at 5-7 days.	dichotomous clinical outcome on the mRS and infarct size at 5-7 days.	
5	Introduction and rationale	Currently the role of IVT in acute ischemic stroke treatment with IAT is unclear. The incidence of bleeding complications was similar in MR CLEAN to the frequency in the NINDS IVT trial and SITS MOST registry (13, 14). In MR CLEAN, the occurrence of symptomatic intracranial hemorrhage (sICH) (7%, fatal in 65%) was similar between the intervention and the control group, suggesting that this complication could not be attributed to the IAT, but rather to pre-treatment with IVT. In 2017, a retrospective ACTUAL study based on Chinese population showed that the incidence of sICH in direct endovascular treatment group and bridging treatment group was higher than that in RCT study of previous IAT (13.8% and 13.0%) (15-20); at the same time, the incidence of aICH in the intravascular treatment group was significantly lower than that in the bridging treatment group (28.3% vs. 44.9%, P=0.01). This may be related to the distribution characteristics of the cause of stroke in Chinese population. In the Asian population ischemic stroke reported, the proportion of intracranial atherosclerotic stenosis was as high as 30 - 50%, which was significantly higher than that of other populations (21-25). The high incidence of	Currently the role of IVT in acute ischemic stroke treatment with IAT is unclear. The incidence of bleeding complications was similar in MR CLEAN to the frequency in the NINDS IVT trial and SITS MOST registry [13, 14]. In MR CLEAN, the occurrence of symptomatic intracranial hemorrhage (sICH) (7%, fatal in 65%) was similar between the intervention and the control group, suggesting that this complication could not be attributed to the IAT, but rather to pre-treatment with IVT. According to the meta-analysis of the five RCT results, the incidence of symptomatic intracranial hemorrhage in westerners was 4.4%. however, there are differences in the pathogenesis of stroke between eastern and western populations. In 2017, a retrospective ACTUAL study based on Chinese population showed that 44.3% acute intracranial artery occlusion is caused by atherosclerosis, which was significantly higher than westerners. At the	Detail modified

		intracranial atherosclerotic stenosis implied that the use proportion of intracranial stent implantation and GP2b3a receptor antagonist increased significantly. In ACTUAL study, the proportion of stent implantation in direct endovascular treatment group and bridging treatment group was 22.5% and 23.2% respectively, and the use proportion of GP2b3a receptor antagonists was 20.3% and 10.9% respectively. Whether atherosclerotic stenosis can affect the efficacy of IVT, and whether the increase in the proportion of stent implantation will increase the incidence of ICH after IVT, which are currently unknown and need to be studied. The incidence of sICH between the two groups was similar, and whether suggesting that the occurrence of sICH could not be attributed to the IAT, but rather to pre-treatment with IVT. Further, IVT could have other potential deleterious effects such as neurotoxicity and loss of blood brain barrier integrity. (26) If IVT softens the thrombus prior to IAT, this could also lead to increased fragmentation rates, making successful reperfusion more difficult to achieve. Last, but not least, we know from EM scanning studies that fibrin forms around the struts of a stent retriever when in position. Systemic alteplase treatment may impair this fibrin formation	same time, there was no significant difference in the incidence of sICH between direct endovascular treatment group and bridging treatment group. This may remind us that the increased proportion of acute intracranial atherosclerotic occlusion did not significantly influence the incidence of sICH. whether the increase of the stent implantation proportion will affect clinical outcome is unknown. In the ACTUAL study, the incidence of aICH in the intravascular treatment group was significantly lower than that in the bridging treatment group (28.3% vs. 44.9%, P=0.01). whether the increase in the proportion of stent implantation will increase the incidence of ICH after IVT, which are currently unknown and need to be studied. According to the above comprehensive analysis, we hypothesize that direct IAT, without pretreatment with IVT, in selected patients may lead to an 4% absolute increase in good outcome because of a reduction in the occurrence of sICH and an increase in treatment effect of IAT.	

		and adversely affect the thrombectomy results .<br>We hypothesize that direct IAT, without pretreatment with IVT, in selected patients may lead to an 8% absolute increase in good outcome because of a reduction in the occurrence of sICH and an increase in treatment effect of IAT.		
6	Study objectives	The primary objective of this trial is to assess the effect of direct IAT compared with IVT followed by IAT, on functional outcome in patients with AIS, caused by an anterior circulation occlusion that is confirmed by **neuro-imaging**.	The primary objective of this trial is to assess the effect of direct IAT compared with IVT followed by IAT, on functional outcome in patients with AIS, caused by an anterior circulation occlusion that is confirmed by **CTA**.	Clearly define CTA as preoperative imaging evaluation
7	Sample size calculation	We based our estimations on the distribution of the modified Rankin Scale (mrS) in the control group of the trial, which we derived from the intervention group of the MR CLEAN trial (9): mRS 0: 3%; mRS 1: 9%; mRS 2: 21%; mRS 3: 18%; mRS 4: 22%; mRS 5: 6% and mRS 6: 21%. We assumed a favorable treatment effect with a common odds ratio (cOR) of 1.54, which corresponds to an absolute risk difference of having a score on the modified Rankin Scale of 0-2 of approximately 8%. The main purpose is to demonstrate non-inferiority, that is, the lower limit of the 95% confidence interval does not cross the pre-specified cOR non-inferiority Cutoff of 0.8. In a simulation with 5000 runs we computed the	We assumed a favorable treatment effect with a common odds ratio (cOR) of 1.163, corresponding to a 4% absolute increase in the rate of mRS scores of 0-2. The main aim of the trial is to demonstrate non-inferiority. To do so, the lower limit of the two-sided 95% confidence interval of the cOR should not cross the pre-specified non-inferiority boundary of 0.8.<br>In a Monte Carlo simulation with 5000 runs we computed the proportion of positive trials, for a given sample	Recalculate the sample size according to the modified good outcome

		proportion of positive trials, for a given sample size. This yielded a sample size of 680, providing 99% power to detect a true treatment effect, with two-sided alpha =0.05. In the analysis we will use covariate adjustment, which reduces the required sample size with 25% (28, 29). Therefore, the aim is to include 540 patients, 270 in each group of the trial, considering a dropout rate of 5%.	size. A sample size of 710 was determined to detect the pre-defined non-inferiority with a power of 80% and two-sided alpha of 0.05. Using covariate adjustment with at most 25%, a conservative 15% sample size reduction can be achieved, plus 5% dropout rate, leading to a final sample size of 636, 318 per arm.	
8	Investigational treatment	The target time from study non contrast CT to groin puncture will be as fast as possible. All patients must undergo groin puncture within a median of 60 minutes after non-contrast CT acquisition.	The target time from study randomization to groin puncture will be as fast as possible. All patients must undergo groin puncture within a median of 60 minutes after randomization.	Detail modified
9	Name and description of non-investigational products	Stent-retrievers for IAT are the background treatment in this trial. **The devices listed below** may be used as primary device for IAT.<br>Revive stent **Codman/DePuy-Synthes**	Stent-retrievers for IAT are the background treatment in this trial. **The devices approved by CFDA during the research** may be used as primary device for IAT, which is shown in the table below.<br>Revive **Johnson & Johnson/ Cerenovus**	Detail modified
10	Secondary outcomes	Score on the NIHSS at **24-72 hours** and 5-7 days, or at discharge	Score on the NIHSS at **24 ±6 hours** and 5-7 days, or at discharge	Revise the time window for NIHSS score
11	Other study parameters	medication including **antihypertensive treatment**, antiplatelet agents and anticoagulants	medication including ~~**antihypertensive treatment**~~, antiplatelet agents and anticoagulants	There is no need to collect the information on antihypertensive drugs
12	Other study	laboratory examination including	laboratory examination	Detail modified

	parameters	INR, APTT, **C-reactive protein,** glucose, creatinine	including INR, APTT, ~~**C-reactive protein,**~~ glucose, creatinine	
13	Other study parameters	Last, during the 90 day study period, information regarding the direct treatment cost will be collected.	delete	There is no need to collect the treatment cost.
14	Study procedures	All patients will undergo assessment of the NIHSS at baseline, **24-72 hours** and 5-7 days, which is routine in clinical procedure.	All patients will undergo assessment of the NIHSS at baseline, **24±6 hours** and 5-7 days, which is routine in clinical procedure.	Revise the time window for NIHSS score
15	Serious adverse events (SAEs)		The investigators should report to the sponsor within 24 hours after being informed of this adverse event of special interest. If this adverse event of special interest also meets the above SAE definition, it shall also be reported to the Ethics Committee of this site.	Add explanation for sponsor special interest adverse event.
16	Data Safety Monitoring Board (DSMB)	The DSMB will meet frequently, **at least annually or after inclusion of the next 100 patients (whichever comes first)** and assess the occurrence of adverse events by center and by procedure.	DSMB plans to conduct two interim analyses to evaluate the treatment effect and the incidence of adverse reactions according to the procedure at the end of the 90-day follow-up of **1/3 and 2/3 subjects**, respectively.	Revise the interim analyses plan
17	Recruitment and consent	In view of half of the AIS patients have language impairment, lack of sense of disease, or other acute cognitive symptoms, following the first paragraph of Article 23 of "Standard for quality management of medical device clinical trials" (June 1, 2016), for incapacitated subjects, if the ethics committee agrees in	deleted	Detail modified

		principle, and investigators believe that subjects participating in clinical trials are in their own interest, they can also enter the clinical trial, but their guardians should sign the name and date before the trial;		
18	Problems of minors or incapacitated subjects	In such case, we will inform the patient and the legal representative, and seek the latter's written consent, as described in 11.2.	In such case, following the first paragraph of Article 23 of "Standard for quality management of medical device clinical trials" (June 1, 2016), for incapacitated subjects, if the ethics committee agrees in principle, and investigators believe that subjects participating in clinical trials are in their own interest, they can also enter the clinical trial, but their guardians should sign the name and date before the trial.	Add GCP identification
19	Benefits and risks assessment, group relatedness	The expected benefit from direct intra-arterial treatment compared to IVT followed by IAT may amount to **8%** absolute increase in independent living at 3 months.	The expected benefit from direct intra-arterial treatment compared to IVT followed by IAT may amount to **4%** absolute increase in independent living at 3 months.	Revise the expected benefit
20	Compensation for injury	The insurance applies to the damage that becomes apparent during the study or within 4 years after the end of the study.	deleted	deleted
21	Handling and storage of data and documents	All data will be entered into a web-based database (**OpenClinica**) by local research personnel.	All data will be entered into a web-based database (EDC) by local research personnel.	Detail modified
22	Public disclosure and publication policy		NCT03469206<br>Clinicaltrials: NCT03469206	Detail modified
23	Study committees	Steering Committee<br>Members: Prof Deng Benqiang,	Steering Committee<br>Members: Prof Deng	Modify member list

		Changhai Hospital Affiliated to the Second Military Medical University; Prof Yang Pengfei, Changhai Hospital Affiliated to the Second Military Medical University; Prof Zhang Yongwei, Changhai Hospital Affiliated to the Second Military Medical University; and Prof Hong Bo, Changhai Hospital Affiliated to the Second Military Medical University	Benqiang, Changhai Hospital Affiliated to the Second Military Medical University; Prof Hong Bo, Changhai Hospital Affiliated to the Second Military Medical University; Prof Charles Majoie, Academisch Medisch Centrum bij de Universitieit van Amsterdam (AMC); and Prof Yvo Roos, Academisch Medisch Centrum bij de Universitieit van Amsterdam (AMC); and Prof Hong Bo, Changhai Hospital Affiliated to the Second Military Medical University	
24	Study Committee		Data Safety Monitoring Board<br>Chairman: Prof. Craig Anderson, The George Institute for Global Health at Peking University Health Science Center<br>Members: Prof. Miao Zhongrong, Beijing Tiantan Hospital Affiliated to Capital Medical University; Prof. He Jia, Department of Health Statistics of Second Military Medical University	Detail modified
25	Study committee		Outcome committee<br>Chairman: Prof. Li Yansheng, Renji Hospital of Shanghai Jiaotong University School of Medicine<br>Members: Prof. Zhang	Detail modified

<table>
<tr><td></td><td></td><td></td><td>Ping, Changhai Hospital Affiliated to the Second Military Medical University; Prof. Zhang Yingying, Huadong Hospital Affiliated to Fudan University.</td><td></td></tr>
<tr><td>26</td><td>Study committee</td><td></td><td>Adverse Event Adjudication Committee
Chairman: Prof. Fang Qi, The First Affiliated Hospital of Soochow University
Members: Prof. Zhang Yongwei, Changhai Hospital Affiliated to the Second Military Medical University; Prof. Fu Jianhui, Huashan Hospital Affiliated to Fudan University</td><td>Detail modified</td></tr>
<tr><td>27</td><td>General</td><td>Inclusion in the trial, randomization, and subsequent endovascular treatment with/without prior IVT should be started as soon as possible after presentation in all eligible patients. The time-path below gives an indication about how soon the following steps need to take place in the most optimal situation.
<table><tr><th>Procedures</th><th>Time path</th></tr><tr><td>Arrival at ER</td><td>0</td></tr><tr><td>Randomization</td><td>10</td></tr><tr><td>Start neuro imaging</td><td>10 min</td></tr><tr><td>Start IV alteplase

(if so randomized)</td><td>20 min</td></tr><tr><td>Groin puncture</td><td>70 min</td></tr></table></td><td>Inclusion in the trial, randomization, and subsequent endovascular treatment with/without prior IVT should be started as soon as possible after presentation in all eligible patients. The target time from study random to groin puncture will be as fast as possible. All patients would be better to undergo groin puncture within a median of 60 minutes after non-contrast CT acquisition.</td><td>Delete the time-path</td></tr>
<tr><td>28</td><td>Type of mechanical thrombectomy device(s)</td><td>All stent retriever and aspiration devices for IAT, which are approved for this purpose by</td><td>Stent-retrievers for IAT are the preferred treatment in this trial.</td><td>Detail modified</td></tr>
</table>

		CFDA, and have been approved for use in the study by the steering committee are allowed in the trial as a first line of defense and are listed below:<br>Revive stent **Codman/DePuy-Synthes**<br>**A second device** is allowed as a second option, when the first device has failed according to the interventionist. The further choice of the particular device for a certain patient is left to the discretion of the interventionist.	During the study period, all CFDA approved stent-retrievers can be used as the preferred device for IAT, as shown in the following table (not limited to the following table).<br>Revive **Johnson & Johnson/ Cerenovus**<br><br>**Other devices** are allowed as a second option, when the first device has failed according to the interventionist. The further choice of other devices for a certain patient is left to the discretion of the interventionist.	

Summary of protocol amendments（version 3.0）

	SECTION	Original version	Modified version	Explanation
1	Front cover	Version: 2.0<br>Date：AUG 31, 2018	Version: 3.0<br>Date:AUG 20 2019	revised version
2	Subgroup analysis	Extracranial carotid obstruction	Ipsilateral extracranial carotid tandem lesion	To indicate more specifically the subgroup for studies
3	Subgroup analysis	Thrombus perviousness	Thrombus characteristics (thrombus perviousness, clot burden, density)	To describe more specifically the studies of its nature in imaging of thrombosis
4	Subgroup analysis		Large vessel occlusion due to different etiologies	This is a newly added analytical subgroup which has no material impacts on the overall progress of the research
5	Figure2 Patient Flow in the Trial	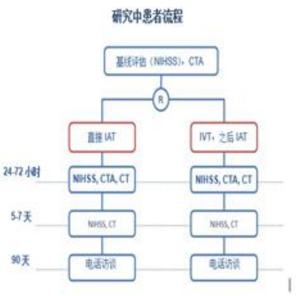	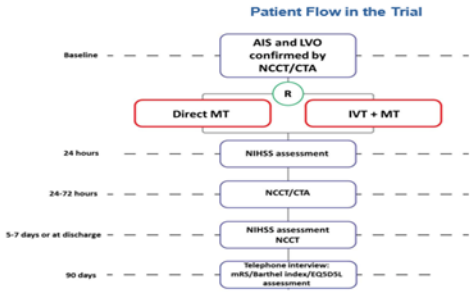	Detail modified
6	Study Committee		Imaging Assessment Committee<br>Chairman: Prof. Jianping Lu, Department of Radiology, Changhai Hospital, Naval Medical University<br>Members:<br>Prof. Bing Tian, Department of Radiology, Changhai Hospital, Naval Medical University	An image evaluation sub-committee was added in the English version

			Yongxin Zhang, Department of Neurosurgery, Changhai Hospital, Naval Medical University<br>Lei Zhang, Department of Neurosurgery, Changhai Hospital, Naval Medical University<br>Hao Wang, Department of Neurology, Linyi People's Hospital<br>Zhang Shi, Department of Radiology, Changhai Hospital, Naval Medical University<br>Wenjia Peng, Department of Radiology, Changhai Hospital, Naval Medical University<br>Xuefeng Zhang, Department of Radiology, Changhai Hospital, Naval Medical University<br>Xia Tian, Department of Radiology, Changhai Hospital, Naval Medical University<br>Tengfei Zhou, Department of Radiology, Henan Provincial People's Hospital<br>Xiaoquan Xu, Department of Radiology, Jiangsu Provincial People's Hospital<br>Shenqiang Yan, Department of Neurology, Second Affiliated Hospital; Zhejiang University College of Medicine<br>Jun Ke, Department of Radiology, First Affiliated Hospital, Soochow University<br>Guang Zhang, Department of Neurosurgery, First Affiliated Hospital, Harbin Medical University<br>Jun Shi, Core lab, Cardiovascular Chinese research center (CCRC)<br>Fang Li, Core lab, Cardiovascular Chinese research center (CCRC)<br>Xin Wang, Core lab, Cardiovascular Chinese research center (CCRC)	

STATISTICAL ANALYSIS PLAN(SAP)

Protocol Number:	CH01
Version Status (Draft /Final / Amendment):	1.0 / Final
Date:	30-Nov-2018

Direct Intra-arterial thrombectomy in order to Revascularize AIS patients with large vessel occlusion Efficiently in Chinese Tertiary hospitals: a Multicenter randomized clinical Trial (DIRECT-MT)

Study Statistician (CRO):

Sam Zhong

Shanghai KNOWLANDS MedPharm Consulting Co., Ltd.

Sponsor:

Changhai Hospital Affiliated to the Second Military Medical University

SIGNATURE PAGE

Direct Intra-arterial thrombectomy in order to Revascularize AIS patients with large vessel occlusion Efficiently in Chinese Tertiary hospitals: a Multicenter randomized clinical Trial (DIRECT-MT)

Study Statistician (CRO)

Sam Zhong<br>Shanghai KNOWLANDS MedPharm Consulting Co., Ltd.	Date

SIGNATURE PAGE

Direct Intra-arterial thrombectomy in order to Revascularize AIS patients with large vessel occlusion Efficiently in Chinese Tertiary hospitals: a Multicenter randomized clinical Trial (DIRECT-MT)

Sponsor

Pengfei Yang	Date
Changhai Hospital Affiliated to the Second Military Medical University	

TABLE OF CONTENTS

TITLE PAGE **110**
TABLE OF CONTENTS **113**
LIST OF ABBREVIATIONS **115**
1 STUDY OVERVIEW **117**
1.1 STUDY DESIGN AND RANDOMIZATION 117
1.2 STUDY OBJECTIVES 118
1.3 STUDY OUTCOMES 118
1.4 SAMPLE SIZE CALCULATION 119
1.5 STUDY PROCEDURES 120
1.5.1 Procedures 120
2 STATISTICAL METHODOLOGY **120**
2.1 STATISTICAL VARIABLES 121
2.1.1 Background and demographic characteristics 121
2.1.2 Efficacy 121
2.1.3 Safety 122
2.1.4 Health economics 123
2.2 STATISTICAL ANALYSIS POPULATION 123
2.2.1 Full analysis set 123
2.2.2 Per-protocol set 124
2.2.3 Subject disposition 124
2.3 STATISTICAL METHODS 125
2.3.1 Demography and baseline characteristics 125
2.3.2 Analysis of efficacy outcomes 125
2.3.3 Study treatment 127
2.3.4 Safety analysis 127
2.3.5 Analysis of quality of life 128
2.4 DATA PROCESSING CONVENTIONS 129
2.4.1 Definition of baseline 129
2.4.2 Missing data 129
2.4.3 Time window 129
2.4.4 Unscheduled visits 129
2.4.5 Centers pooling 129
3 CHANGES TO PLANNED ANALYSES FROM THE PROTOCOL **130**
4 INTERIM ANALYSIS **131**

5 STATISTICAL ANALYSIS SOFTWARE 132

6 REFERENCES 133

7 APPENDIX 134

Appendix table 1 Modified Rankin Scale 134

Appendix table 2 Extended Treatment In Cerebral Ischemia (eTICI) Scale 135

Appendix table 3 NIH Stroke Scale 136

Appendix table 4 EUROQOL 5D-5L 140

Appendix table 5 Barthel Index 141

Appendix table 6 Classification of Infarct in a New Territory 143

Appendix table 7 Description of Intracranial Hemorrhages 144

Appendix table 8 Modified Arterial Occlusive Lesion Classification 145

Appendix table 9 Collateral Score 146

Appendix table 10 Description of Subgroup Types and Definitions 147

LIST OF ABBREVIATIONS

Abbreviations	Definitions
AComA	Anterior communicating artery
ADL	Activities of Daily Living
AE	Adverse Event
AESI	Adverse Events of Special Interest
AIS	Acute Ischemic Stroke
AOL	Arterial occlusive lesion classification
APTT	Activated Partial Thromboplastin Time
ASPECTS	the Alberta Stroke Program Early CT Score
BI	Barthel Index
CI	Confidence interval
CONSORT	Consolidated Standards of Reporting Trials
cOR	Common odds ratio
CT	Computed tomography
CTA	Computed tomography angiography
CRF	Case Report Form
CRO	Contract Research Organization
DSA	Digital substraction angiography
DSMB	Data Safety Monitoring Board
EC	Ethics committee
eTICI	extended treatment in cerebral ischemia scale
EQ5D-5L	EuroQol-5 dimensions-5 level
EVT	Endovascular treatment
FAS	Full analysis set
IAT	Intra-arterial treatment
INR	International normalized ratio
IVT	Intravenous treatment
LOC	Level of consciousness
mAOL	Modified arterial occlusive lesion classification
MCA	Middle cerebral artery
MedDRA	Medical Dictionary for Drug Regulatory Activities

Abbreviations	Definitions
MRI	Magnetic resonance imaging
mRS	Modified Rankin scale
MT	Mechanical thrombectomy
NCCT	Non-contrast computed tomography
NIHSS	National Institute of Health stroke scale
PPS	Per-protocol set
PT	Preferred term
SAE	Serious adverse event
SAP	Statistical analysis plan
SAS	Statistical analysis system
SD	Standard deviation
sICH	Symptomatic intracerebral hemorrhage
SOC	System organ class

1 STUDY OVERVIEW

This Statistical Analysis Plan (SAP) is developed based on the most recent study protocol (Version 2.0, 31-Aug-2018) and Case Report Form (CRF, Version 1.4, 13-Nov-2018), and details the statistical analysis strategies and methods for the study.

This SAP predefines the statistical analysis population, variables and analysis methods before database lock to ensure the reliability of the study results.

1.1 STUDY DESIGN AND RANDOMIZATION

This is a multicenter prospective randomized clinical trial with open-label treatment and blinded outcome assessment (PROBE). The study will run for 4 years in intervention centers. Randomization will be stratified by center. The treatment allocation is 1:1 for:

- Direct IAT,
- IVT followed by IAT (IVT plus IAT)

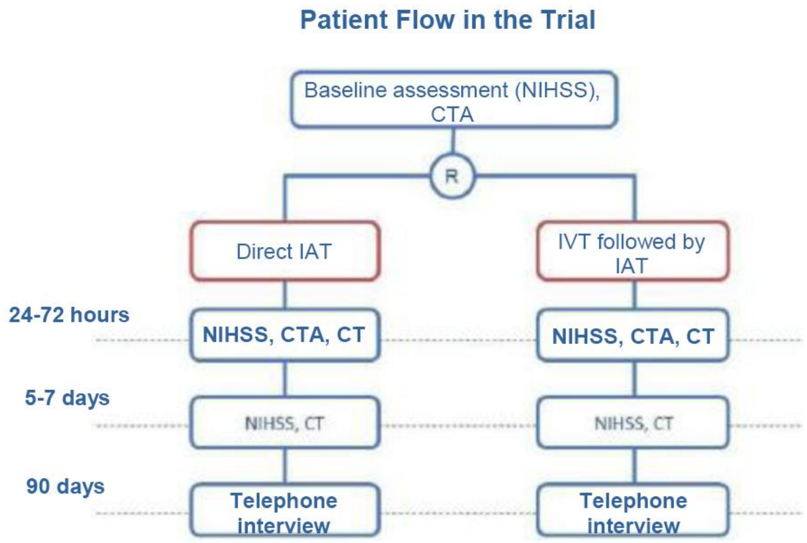

Figure 1 Patient flow in the trial

The intervention group will undergo immediate intra-arterial treatment (IAT) using a stent retriever, as recommended by the steering committee. Patients in the control group will receive alteplase intravenous treatment (IVT) (0.9 mg/kg with a maximum dose of 90 mg), followed by IAT using a stent retriever.
Local application (intra-arterial) of alteplase is allowed in any of the patients included in the DIRECT-MT if necessary. Patients pre-treated with IVT should not receive more

than 30mg alteplase during intra-arterial treatment. Delivery of alteplase in shots of 5 mg in 5-10 minutes intervals is recommended. An equivalent dose of 400,000 U urokinase, delivered in shots of 50.000 - 100.000 U, in 5-10 minutes time intervals, is also accepted as escape medication in individual cases. If successful reperfusion (eTICI 2b-3) is not achieved in the direct IAT group, IVT with 0.9 mg/kg may be initiated if the 4.5 hour window or maximum dose is not exceeded.

1.2 STUDY OBJECTIVES

The primary objective of this trial is to assess the effect of direct IAT compared with IVT followed by IAT, on functional outcome in patients with acute ischemic stroke (AIS), caused by an anterior circulation occlusion that is confirmed by Computed tomography angiography (CTA).
The secondary objective is to explore for superiority of direct IAT relative to IVT followed by IAT.
The tertiary objective is to assess the effect of direct IAT compared with IVT with IAT on neurological recovery (NIHSS), infarct size and occurrence of Symptomatic intracerebral hemorrhage (sICH).
The fourth objective is to collect thrombi and to analyze them with respect to their potential for treatment effect modification.

1.3 STUDY OUTCOMES

Primary outcome:
The primary outcome is the score on the modified Rankin Scale (mRS) (Table 1 in Appendix) at 90 days (± 14 days). The mRS is the preferred disability parameter for clinical trials in stroke. The mRS is an ordinal hierarchical scale incorporating six categories from 0 up to and including 5, and describes the range of disability encountered post stroke. "Death" is assigned a score of 6. Assessment of outcome on the mRS will be performed by outcome committee, blinded to the allocated and actually received treatment. Their assessment will be based on standardized reports of a telephone interview by trained research personnel who are not aware of treatment allocation.

Secondary outcomes:

- Death within 90 days (± 14 days)
- Pre-interventional recanalization
- extended treatment in cerebral ischemia scale (eTICI) score on final angiography of IAT (Table 2 in Appendix)
- Recanalization rate at 24-72 hours, assessed with CTA

- Score on the NIHSS at 24±6 hours and 5-7 days. (Table 3 in Appendix)
- Final infarct volume at 5-7 days. Final infarct volume will be assessed with the use of an automated, validated algorithm. Infarct size at day 5-7 will be compared with plain computed tomography (CT) and perfusion CT results (if available) at baseline.
- Dichotomized mRS of 0-1 vs. 2-6 at 90 days (±14 days)
- Dichotomized mRS of 0-2 vs. 3-6 at 90 days (±14 days)
- Dichotomized mRS of 0-3 vs. 4-6 at 90 days (±14 days)
- Score on the EuroQol-5 dimensions-5 level (EQ5D-5L) (Table 4 in Appendix) and Barthel index (BI) (Table 5 in Appendix) at 90 days (±14 days)

Safety outcomes:

- Hemorrhages according to the Heidelberg criteria [1]
- sICH scored according to the Heidelberg criteria [2]
- Embolization in new territory on angiography during IAT
- Occurrence of aneurysma spurium
- Occurrence of groin hematoma
- Infarction in new territory at 5-7 days (Table 6 in Appendix)
- Death from all causes within 90 days (±14 days)

1.4 SAMPLE SIZE CALCULATION

We based our estimations on the distribution of the mRS in the control group of the trial, which we derived from the intervention group of the MR CLEAN trial [3]: mRS 0: 3%; mRS 1: 9%; mRS 2: 21%; mRS 3: 18%; mRS 4: 22%; mRS 5: 6% and mRS 6: 21%. We assumed a favorable treatment effect with a common odds ratio (cOR) of 1.163, corresponding to a 4% absolute increase in the rate of mRS scores of 0-2. The main purpose is to demonstrate non-inferiority, that is, the lower limit of the two-side 95% confidence interval does not cross the pre-specified cOR non-inferiority Cutoff of 0.8. In a simulation with 5000 runs we computed the proportion of positive trials, for a given sample size. A sample size of 710 was determined to detect the pre-defined non-inferiority with a power of 80% and two-sided alpha of 0.05. Using covariate adjustment with at most 25%, a conservative 15% sample size reduction can be achieved, plus 5% dropout rate, leading to a final sample size of 636, 318 per arm.

1.5 STUDY PROCEDURES

Before starting the study, patients or their guardians must read and sign the informed consent approved by the current Ethics Committee (EC). All research steps should be carried out within the time window specified in the study protocol.

All patients will undergo assessment of the NIHSS at baseline, 24±6 hours and 5-7 days, which is routine in clinical procedure. It will be carried out by certified assessors. Patients will undergo NCCT and CTA at baseline. After 24-72 hours, CTA is repeated to determine recanalization. At 5-7 days, patients will undergo non-contrast computed tomography (NCCT) to assess infarct size.

In addition, this trial also makes use of "waste material": retrieved thrombi during intervention. These thrombi will be stored in the participating study centers for follow-up analysis.

1.5.1 Procedures

All the procedures to be recorded are listed in Table 1.

Table 1 Procedures

Items	Procedures(includes but not limited to:)
Demography	Date of birth (based on valid identity documents), sex, age
Medical History	Disease history, smoking/alcohol drinking history, medications
Modified Rankin Scale	Disability level, ranging from 0~5
Glasgow coma Scale	Eye Opening, Best Verbal Response, Best Motor Response
Vital Signs	Systolic/diastolic blood pressure, heart rate, body temperature, height, weight
NIHSS	Level of consciousness (LOC), LOC Questions, LOC Commands, Best Gaze, Visual, Facial palsy, Motor arm, Motor leg, Limb ataxia, Sensory, Best language, Dysarthria, Extinction and Inattention
Laboratory tests	Serum glucose, Activated Partial Thromboplastin Time (APTT), International normalized ratio (INR), Thrombocyte count, Serum creatinine
eTICI	eTICI classification includes 0, 1, 2a, 2b, 2C and 3
EQ5D-5L score	Mobility, Self-Care, Usual-Activities, Pain/Discomfort, Anxiety/Depression
BARTHEL index	Feeding, Bathing, Grooming, dressing, Bowels, Bladder, Toilet use, Transfers(bed to chair and back), Mobility(on level surfaces), Stairs
Neuroimaging	CT, CTA, MRI and other imaging examinations

2 STATISTICAL METHODOLOGY

2.1 STATISTICAL VARIABLES

2.1.1 Background and demographic characteristics

The demographic and baseline information will include age, sex, medical history, smoking history and medications used at home.

2.1.2 Efficacy

2.1.2.1 Primary efficacy variables

Primary efficacy outcome is mRS score change at 90 days (±14 days), which will be blindly evaluated by an independent Outcome Assessment Committee.

The mRS is the preferred disability parameter for clinical trials in stroke. The mRS is an ordinal hierarchical scale incorporating six categories from 0 up to and including 5, and describes the range of disability encountered post stroke. "Death" is assigned a score of 6 (Table 1 in Appendix).

2.1.2.2 Secondary efficacy variables

- Death within 90 days (±14 days)
- Pre-interventional recanalization
- eTICI score on final angiography of IAT. (Table 2 in Appendix)
- Recanalization rate at 24-72 hours, assessed with CTA
- Score on the NIHSS at 24±6 hours and 5-7 days. (Table 3 in Appendix)
- Final infarct volume at 5-7 days. Final infarct volume will be assessed with the use of an automated, validated algorithm. Infarct size at day 5-7 will be compared with plain CT and perfusion CT results (if available) at baseline.
- Dichotomized mRS of 0-1 vs. 2-6 at 90 days (±14 days)
- Dichotomized mRS of 0-2 vs. 3-6 at 90 days (±14 days)
- Dichotomized mRS of 0-3 vs. 4-6 at 90 days (±14 days)
- Score on the EQ5D-5L and Barthel index at 90 days (±14 days)

Pre-interventional recanalization： Recanalization rate (eTICI 2b, 2c or 3) before patients received mechanical intra-arterial treatment according to the DSA.

Recanalization rate at 24-72 hours: defined as the proportion of patients in whom recanalization as determined on 24-72 hours CTA is achieved.
eTICI score: eTICI assessment will be performed post IAT. The eTICI classification includes 0, 1, 2a, 2b, 2c and 3 (Table 2 in Appendix).

NIHSS score: The NIHSS is an ordinal hierarchical scale to evaluate the severity of stroke by assessing a patient's performance. Scores range from 0 to 42, with higher scores indicating a more severe deficit (Table 3 in Appendix). NIHSS assessment will be performed at baseline, 24±6 hours post operation and 5-7 days post operation.

The EuroQoL 5-dimensions 5-level (EQ-5D-5L) questionnaire is a standardized measure of health outcome that has been used extensively in patients with stroke (Table 5 in Appendix). EQ5D-5L assessment will be performed at 90±14 days post operation.

The Barthel index (BI) is an ordinal scale used to measure performance in 10 activities of daily living (ADL). Test scores range from 0 to 100, with higher scores indicating better performance in these daily activities (Table 4 in Appendix). BI assessment will be performed at 90±14 days post operation.

2.1.3 Safety

2.1.3.1 Adverse events (AEs)

This study focused on the serious adverse events (SAEs) and Adverse Events of Special Interest (AESIs), and all reported SAEs will be blindly reviewed by an independent Adverse Event Committee.

All SAEs and AESIs will be coded using MedDRA 22.0 or higher, before database lock. MedDRA System Organ Class (SOC) and Preferred Term (PT) will be summarized.

Classification of serious adverse events

All SAEs will be classified as follows,

- Death
- Symptomatic intracranial hemorrhage
- De novo Ischemic Stroke
- Large or malignant middle cerebral artery (MCA) infarction
- Pneumonia (Aspiration and others)
- Contrast allergic reaction
- Major bleeding due to femoral artery access complications including groin hematoma, retroperitoneal hematoma
- Acute kidney injury
- Others

Adverse Events of Special Interest

Adverse events of special interest for this study include aspiration pneumonia and allergic contrast reactions.

2.1.3.2 Laboratory variables

Baseline laboratory tests will be conducted at screening visit, which include blood glucose (mmol/L), prothrombin time (sec), international standardized ratio, platelet count (*10^9), serum creatinine (umol/L).

2.1.3.3 Vital signs

At screening visit, the following vital signs will be measured: systolic blood pressure (mmHg), diastolic blood pressure (mmHg), heart rate (beat/min), body temperature (℃), height (cm), weight (kg).

2.1.3.4 Neuroimaging

CT and CTA will be performed at baseline and follow-up visit and the findings of which will be blindly evaluated blindly by an independent Imaging Committee (Core lab), including hyperdense, the Alberta Stroke Program Early CT Score (ASPECTS), another occlusion location of anterior circulation except target lesion, anterior communicating artery (AComA), intracranial hemorrhages (Table 7 in Appendix), midline shift present, target vessel stent placement, modified arterial occlusive lesion classification (mAOL, (Table 8 in Appendix)), vascular occlusion, etc.
The newly affected territory of the middle cerebral artery was graded by the systematic quantitative scoring system, e.g. ASPECTS. It will be performed at baseline visit and follow-up visit. ASPECTS is allotted 10 points, including caudate, lentiform, internal capsule, insular cortex, M1, M2, M3, M4, M5, M6. One point is subtracted for an area of early ischaemic change, such as focal swelling, or parenchymal hypoattenuation, for each of the defined regions. A score of 0 indicates diffuse ischaemia throughout the territory of the middle cerebral artery.

2.1.4 Health economics

None.

2.2 STATISTICAL ANALYSIS POPULATION

The analysis populations include the full analysis set (FAS) and per-protocol set (PPS) for this study.

2.2.1 Full analysis set

All subjects who were randomized will be included in the full analysis set (FAS) according to intention-to-treat principles, in which subjects will be analyzed

according to the group assigned by randomization. FAS is the primary efficacy analysis set for this study.

2.2.2 Per-protocol set

Per-protocol set (PPS) is a subset of FAS, including all randomized subjects who have been treated in the study without major protocol deviations that may significantly impact the interpretation of efficacy results. Detailed protocol deviation criteria will be determined at the latest before database lock. PPS will be used for the primary efficacy outcome and safety analysis. Subjects entering PPS need to satisfy all the following basic criteria:

(1) Meet all the eligibility criteria specified in the study protocol;

(2) The subjects were randomized and received the assigned treatment.

(3) Underwent groin puncture, with exception of patients with clinical recovery precluding endovascular treatment EVT (due to presumed recanalization before mechanical thrombectomy).

2.2.3 Subject disposition

The number and proportion of screened, randomized, treated and analyzed subjects will be provided. Where necessary, the CONSORT flow chart will be presented to describe the subject disposition in the statistical analysis report.

2.3 STATISTICAL METHODS

For normally distributed continuous data, the following statistics will be provided: number, mean, standard deviation (SD), minimum and maximum. For non-normally distributed continuous data, number, median, lower quartile (Q1), upper quartile (Q3) will be provided, unless otherwise stated. Categorical data will be summarized in terms of the number of patients and percentages.

For summary statistics, mean, standard deviation, median and quartiles will be reported to 1 more decimal place than the original data, while the 95% confidence interval (CI) will be reported to 2. Minimum and maximum values will be reported to the same number of significant digits as the original data. In the frequency table, the percentages will keep 1 decimal, the p values keep 4 decimal or displayed as "<0.0001".

2.3.1 Demography and baseline characteristics

Demography and baseline characteristics will be statistically summarized by treatment group.

2.3.2 Analysis of efficacy outcomes

All efficacy data analyses will be based on FAS and for primary endpoint PPS will also be used.

2.3.2.1 Primary efficacy outcome

The primary effect parameter is the common odds ratio, which will be estimated by ordinal logistic regression (proportional odds model), which represent the shift on the full distribution of the modified Rankin Scale at 90±14 days. Estimations will be adjusted by known prognostic variables such as age (median), pre-stroke mRS (continuous), time from symptom onset to randomization ("<=Q1", ">Q1, <=Q2", ">Q2, <=Q3", ">Q3"), stroke severity (NIHSS, median) and collaterals (Grade 0-1, Grade 2-3). Adjusted and unadjusted estimations and their corresponding 95% confidence intervals will be reported. To assess non-inferiority of direct IAT compared to IVT with IAT, we will assess whether the 95% CI lower bound of the adjusted common odds ratio cross our pre-specified non-inferiority boundary (0.8).
The following SAS procedure will be used for ordered logistic regression analysis (proportional odds model):

```
Proc    logistic data=XXX;
Class TRT FactorA …;
Model mRS90= TRT AGE FactorA …;
Run;
```

2.3.2.2 Secondary efficacy outcome

Continuous secondary efficacy outcomes are mainly infarct size at 5-7 days after operation and recanalization rate before intervention as well. Analysis for these outcomes will be mainly based on statistical descriptions. Where necessary, analysis of variance or corresponding non-parametric test will be used for between-group comparisons. If applicable, the linear regression analysis will be used with adjustment for the same covariate variables as the primary outcome analysis. When deemed necessary, log or other common transformation of non-normal distribution will be used.

Categorical secondary outcomes include mortality at 90 days after operation, recanalization rate at 24-72 hours, dichotomized mRS score at 90 days after operation (0-1 vs. 2-6, 0-2 vs. 3-6, 0-3 vs. 4-6), successful recanalization before and after IAT, and eTICI score at IAT final angiography. Chi-square test will be used for comparison between the two groups, or Fisher's exact test will be used for comparison when applicable. The categorical secondary outcomes will be analyzed by logistic or ordered regression analysis to provide a common odds ratio and its confidence interval, if applicable. The adjustment method is the same as that in the primary outcome analysis.

2.3.2.3 Subgroup analysis

Pre-specified subgroup analysis will be performed by examining the interaction between specific baseline characteristics and treatment. Baseline grouping factors for subgroup analysis include, but are not limited to:

- Quartiles of time from onset of symptoms to randomization
- Quartiles of time from onset of symptoms to groin puncture
- Quartiles of time from randomization to groin puncture
- Quartiles of time from onset of symptoms to revascularization
- Quartiles of time from randomization to revascularization
- Ipsilateral extracranial carotid tandem lesion
- Occlusion location
- Collaterals (Table 9 in Appendix)
- Thrombus perviousness

See the detailed description of subgroup types and definitions in Appendix Table 10.

2.3.2.4 Multiplicity

This study does not consider multiplicity issues and therefore does not adjust significance levels based on multiplicity tests, unless specified otherwise.

2.3.3 Study treatment

2.3.3.1 Intravenous alteplase therapy

Intravenous alteplase therapy will be summarized (only applied to IVT plus IAT group), including whether IVT is performed, planned alteplase dose (mg) and residual alteplase volume (ml).

2.3.3.2 Intra-arterial treatment

A descriptive summary of intra-arterial therapy will be provided according to the treatment groups, including anesthesia management, pre-treatment, treatment, eTICI score as determined by final angiography, thrombectomy, intra-operative non-study drugs, stent implantation/balloon dilatation at the intracranial atherosclerosis occlusion site.

2.3.3.3 Digital substraction angiography (DSA)

The results of DSA will be blindly evaluated by the independent Imaging Committee (Core lab) , including but not limited to: ipsilateral extracranial carotid tandem lesion, intracranial arterial occlusions, another occlusion location of anterior circulation except target lesion, arterial occlusive lesion classification (AOL) and intracranial atherosclerosis occlusion, will be summarized according to the treatment groups.

2.3.4 Safety analysis

In this study, the safety analysis will be mainly based on statistical description. All the analyses will be based on PPS.

2.3.4.1 Analysis of adverse events (AEs)

The number and percentage of subjects who had at least one serious adverse event, classification of serious adverse event, adverse events of special interest and classification of adverse events of special interest from study will be provided.

- All SAEs will be summarized by SOC and PT;
- All AESIs will be summarized by SOC and PT;

2.3.4.2 Clinical laboratory data analysis

Laboratory tests included blood sugar, prothrombin time, international standardized ratio, platelet count and serum creatinine.

For continuous laboratory parameters, summary statistics, including number of subjects, mean, standard deviation, median, minimum and maximum will be provided for observed values for each parameter.

If a lab test result is recorded as "<10", then it will be summarized as a value of "5", if applicable; and likewise, ">10" will be summarized as "10".

2.3.4.3 Analysis of vital signs

Summaries of vital signs parameters will be presented by treatment group, using summary statistics, including number of subjects, mean, standard deviation, median, minimum and maximum for observed values for each parameter.

2.3.4.4 Analysis of neuroimaging

ASPECTS (0-10) and change from baseline are continuous variables and will be presented with summary statistics. The frequency table of each point will also be provided by treatment groups.

Other results of CT and CTA will be summarized using frequency table by treatment groups (if necessary).

2.3.5 Analysis of quality of life

2.3.5.1 NIHSS score

NIHSS (0-42) score and change from baseline are continuous variables and will be presented with summary statistics, including number of subjects, mean, standard deviation, median, minimum and maximum, by treatment groups and by visits. Repeated measures of variance analysis will be used to explore the impact of treatment grouping visits and NIHSS baseline levels.

2.3.5.2 EQ5D-5L score

The frequency and percentage of EQ5D-5L scale will be summarized according to each dimension. If necessary, Chi-square test will be used for comparison between the two groups, or Fisher's exact test will be used for comparison when applicable.

2.3.5.3 Barthel index

Barthel score is a continuous variable (0-100) and will be summarized using number, mean, standard deviation, median, minimum and maximum, by treatment groups. The frequency table of each class level will also be provided.

2.4 DATA PROCESSING CONVENTIONS

2.4.1 Definition of baseline

In this study, baseline values are defined as those data collected before intervention (screening visit). When multiple data collections occur during the baseline period, the final data shall prevail in principle, unless explicitly stated.

2.4.2 Missing data

We will report proportions of missing values for all collected variables where needed. Baseline characteristics missing data will be imputed by regression interpolation as appropriate.

If there is a large number of missing data on efficacy and safety, an evaluation on the missing data should be conducted before analysis, and will propose and determine the solution before database lock.

For patients who died within the study period, the worst scores will be assigned for all not-assessed clinical outcome measures in their analyses, as follows Table 2.

Table 2 The worst scores of clinical outcomes

Clinical outcomes	The worst scores
mRS	6
NIHSS	42
The Barthel index	0

2.4.3 Time window

Not applicable.

2.4.4 Unscheduled visits

Not applicable.

2.4.5 Centers pooling

Unless specifically specified, this study will not consider the center effect, so it will not pool and analyze the data of each study center.

3 CHANGES TO PLANNED ANALYSES FROM THE PROTOCOL

At present, there is no change in the statistical analysis part of the protocol (CH01 protocol, Version 2.0, 31-Aug-2018).

4 INTERIM ANALYSIS

A formal interim analysis is planned.
In order to increase the safety of the intervention, the trial will be monitored by an independent Data Safety Monitoring Board (DSMB). The DSMB will be chaired by a neurologist, and include a neuro-interventionist and an independent methodologist/ statistician. The DSMB plans to conduct two interim analyses to evaluate the treatment effect and the incidence of adverse reactions according to the procedure at the end of the 90-day follow-up of 1/3 and 2/3 subjects, respectively. During the period of patient enrollment into the study, interim analyses of mortality and of any other information that is available on major outcomes (including serious adverse events believed to be due to treatment) will be supplied, in strict confidence, to the chairman of the DSMB, along with any other analyses that the DSMB may request. In the light of these analyses, DSMB will advise the chairman of the Steering Committee if, in their view, the randomized comparisons in DIRECT-MT have provided both (i) "proof beyond reasonable doubt" that for all, or for some specific types of patients, one particular treatment is clearly indicated or clearly contraindicated in terms of a net difference in outcome, and (ii) evidence that might reasonably be expected to materially influence patient management. Appropriate criteria of proof beyond reasonable doubt cannot be specified precisely, but a difference of at least 3 standard deviations in an interim analysis of a major outcome may be needed to justify halting, or modifying, the study prematurely. This criterion has the practical advantage that the number of interim analyses is of little importance.
The advice(s) of the DSMB will be sent to the sponsor of the study by the chair of the steering committee. Should the sponsor decide not to fully implement the advice of the DSMB, the sponsor will send the advice to the EC, including a note to substantiate why (part of) the advice of the DSMB will not be followed.

5 STATISTICAL ANALYSIS SOFTWARE

All statistical analysis and summary will be carried out using SAS 9.2 or higher version in this study. Software R 3.3.1 or higher version will be used for drawing plots if applicable.

6 REFERENCES

[1] Mahoney FI, Barthel DW. Functional Evaluation: The Barthel Index. Md State Med J. 1965;14:61-5.

[2] von Kummer R, Broderick JP, Campbell BC, Demchuk A, Goyal M, Hill MD, et al. The Heidelberg Bleeding Classification: Classification of Bleeding Events After Ischemic Stroke and Reperfusion Therapy. Stroke. 2015;46(10):2981-6.

[3] Berkhemer OA, Fransen PS, Beumer D, van den BergL.A, Lingsma HF, Yoo AJ, et al. A randomized trial of intraarterial treatment for acute ischemic stroke. N Engl J Med. 2015372(1): 11-20

7 APPENDIX

Appendix table 1 Modified Rankin Scale

The modified Rankin Scale (mRS) is an ordinal hierarchical scale ranging from 0 to 5, with higher scores indicating more severe disability. A score of 6 has been added to signify death.

Category	Short description	Long description
0	No symptoms	No symptoms
1	Symptoms, no disability	Minor symptoms that do not interfere with lifestyle
2	Slight disability	Slight disability, symptoms that lead to some restriction in lifestyle, but do not interfere with the patient's capacity to look after himself.
3	Moderate disability	Moderate disability, symptoms that significantly restrict lifestyle and prevent totally independent existence
4	Moderately severe disability	Moderately severe disability, symptoms that clearly prevent independent existence though not needing constant attention
5	Severe disability	Severe disability, totally dependent patient requiring constant attention day and night.
6	Death	Death

Appendix table 2 Extended Treatment In Cerebral Ischemia (eTICI) Scale

eTICI grade	Short description	Long description
0	No perfusion	No antegrade flow beyond the point of occlusion
1	Limited reperfusion	Antegrade reperfusion past the initial occlusion, but limited distal branch filling with little or slow distal reperfusion
2a	<50% reperfusion	Antegrade reperfusion of less than half of the occluded target artery previously ischemic territory (eg, in 1 major division of the MCA and its territory)
2b	≥50% and <90% reperfusion	Antegrade reperfusion of more than half of the previously occluded target artery ischemic territory (eg, in 2 major divisions of the MCA and its territories)
2c	≥90% reperfusion	Near complete antegrade reperfusion of the previously occluded target artery ischemic territory, except for slow flow or distal emboli in a few distal cortical vessels
3	100% reperfusion	Complete antegrade reperfusion of the previously occluded target artery ischemic territory, with absence of visualized occlusion in all distal branches

Appendix table 3 NIH Stroke Scale

The NIHSS is an ordinal hierarchical scale to evaluate the severity of stroke by assessing a patient's performance. (23) Scores range from 0 to 42, with higher scores indicating a more severe deficit. Administer stroke scale items in the order listed. Record performance in each category after each subscale exam. Do not go back and change scores. Follow directions provided for each exam technique. Scores should reflect what the patient does, not what the clinician thinks the patient can do. The clinician should record answers while administering the exam and work quickly. Except where indicated, the patient should not be coached (i.e. repeated requests to patient to make a special effort).

Instructions	Scale definition
1a. Level of consciousness. The investigator must choose a response if a full evaluation is prevented by such obstacles as an endotracheal tube, language barrier, orotracheal trauma/bandages. A 3 is scored only if the patient makes no movement (other than reflexive posturing) in response to noxious stimulation.	0 = Alert; keenly responsive.<br>1 = Not alert; but arousable by minor stimulation to obey, answer, or respond.<br>2 = Not alert; required repeated stimulation to attend, or is obtunded and requires strong or painful stimulation to make movements (not stereotyped).<br>3 = Responds only with reflex motor or autonomic effects or totally unresponsive, flaccid and areflexic.
1b. LOC Questions: The patient is asked the month and his/her age. The answer must be correct – there is not partial credit for being close. Phasic and stuporous patients who do not comprehend the questions will score 2. Patients unable to speak because of endotracheal intubation, orotracheal trauma, severe dysarthria from any cause, language barrier, or any other problem not secondary to aphasia are given a 1. It is important that only the initial answer be graded and that the examiners not "help" the patient with verbal or non-verbal clues.	0 = Answers both questions correctly.<br>1 = Answers one question correctly.<br>2 = Answers neither question correctly.
1c. LOC Commands: The patient is asked to open and close the eyes and then to grip and release the non-paretic hand. Substitute another one step command if the hand cannot be used. Credit is given if an unequivocal attempt is made but not completed due to weakness. If the patient does not respond to command, the task should be demonstrated to him or her (pantomime), and the result scored<br>(i.e. follows none, one or two commands). Patients with trauma, amputation, or other physical impediments should be given suitable one-step commands. Only the first attempt is scored.	0 = Performs both tasks correctly.<br>1 = Performs one task correctly.<br>2 = Performs neither task correctly.
2. Best Gaze: Only horizontal eye movements will be tested. Voluntary or reflexive (oculocephalic) eye movements will be scored, but caloric testing is not done. If the patient has a conjugate deviation of the eyes that can be overcome by voluntary or reflexive activity, the score will be a 1. If a patient has an isolated peripheral nerve paresis (CN III, IV or VI), score a 1. Gaze is testable in all aphasic patients. Patients with ocular trauma, bandages, preexisting blindness, or other disorder of visual acuity or fields should be tested with reflexive movements, and a choice made by the investigator. Establishing eye contact and then moving about the patient from side to side	0= Normal.<br>1= Partial gaze palsy; gaze is abnormal in one or both eyes, but forced deviation or total gaze paresis is not present.<br>2= Forced deviation; or total gaze paresis not overcome by the oculocephalic maneuver.

will occasionally clarify the presence of a partial gaze palsy.	
3. Visual: Visual fields (upper and lower quadrants) are tested by confrontation, using finger counting or visual threat, as appropriate. Patients may be encouraged, but if they look at the side of the moving finger appropriately, this can be scored as normal. If there is unilateral blindness or enucleation, visual fields in the remaining eye are scored. Score 1 only if a clear-cut asymmetry, including quadrantanopia, is found. If patient is blind from any cause, score 3.<br>Double simultaneous stimulation is performed in this case.<br>If there is extinction, the patient receives a 1, and the results are used to respond to item 11.	0= No visual loss.<br>1= Partial hemianopia.<br>2= Complete hemianopia.<br>3= Bilateral hemianopia (blind including cortical blindness)
4. Facial palsy: Ask or use pantomime to encourage the patient to show teeth or raise eyebrows and close eyes. Score symmetry of grimace in response to noxious stimuli in the poorly response or non-comprehending patient. If facial trauma/bandages, orotracheal tube, tape or other physical barriers obscure the face, these should be removed to the extent possible.	0 = Normal symmetrical movements.<br>1= Minor paralysis (flattened nasolabial fold, asymmetry on smiling)<br>2= Partial paralysis (total or near-total paralysis of lower face)<br>3= Complete paralysis of one or both sides (absence of facial movement in the upper and lower face).
5. Motor arm: The limb is placed in the appropriate position: extend the arms (palms down) 90 degrees (if sitting) or 45 degrees (if supine). Drift is scored if the arm falls before 10 seconds. The aphasic patient is encouraged using urgency in the voice and pantomime, but not noxious stimulation. Each limb is tested in turn, beginning with the non-paretic arm. Only in the case of amputation or joint fusion at the shoulder, the examiner should record the score as untestable (UN), and clearly write the explanation for this choice.	0= No drift; limb holds 90 (or 45) degrees for full 10 seconds.<br>1= Drift; limb holds 90 (or 45) degrees, but drifts down before full 10 seconds; does not hit bed or other support.<br>2= Some effort against gravity; limb cannot get to or maintain (if cued) 90 (or 45) degrees, drifts down to bed, but has some effort against gravity.<br>3= No effort against gravity; limb falls.<br>4= No movement.<br>UN = Amputation or joint fusion: explain:<br>5a = Left Arm.<br>5b = Right arm.
6. Motor leg: The limb is placed in the appropriate position: hold the leg at 30 degrees (always tested supine). Drift is scored if the leg falls before 5 seconds. The aphasic patient is encouraged using urgency in the voice and pantomime, but not noxious stimulation. Each limb is tested in turn, beginning with the non-paretic leg. Only in the case of amputation or joint fusion at the hip, the examiner should record the score as untestable (UN), and clearly write the explanation for this choice.	0= No drift; leg holds 30-degree position for full 5 seconds.<br>1= Drift; leg falls by the end of the 5-second period but does not hit bed.<br>2= Some effort against gravity; leg falls to bed by 5 seconds, but has some effort against gravity.<br>3= No effort against gravity; leg falls to bed immediately.<br>4= No movement.<br>UN = Amputation or joint fusion: explain:<br>6a. Left Leg<br>6b. Right Leg.
7. Limb ataxia: This item is aimed at finding evidence of a unilateral cerebellar lesion. Test with eyes open. In case of visual defect, ensure testing is done in intact visual field. The finger-nose-finger and heel-shin tests are performed on both sides, and ataxia is scored only if present out of proportion to weakness. Ataxia is absent in the patient who cannot understand	0= Absent.<br>1= Present in one limb.<br>2= Present in two limbs.<br>UN = Amputation or joint fusion: explain:

or is paralyzed. Only in the case of amputation or joint fusion, the examiner should record the score as untestable (UN), and clearly write the explanation for this choice. In case of blindness, test by having the patient touch nose from extended arm position.	
8. Sensory: Sensation or grimace to pinprick when tested, or withdrawal from noxious stimulus in the obtunded or aphasic patient. Only sensory loss attributed to stroke is scored as abnormal and the examiner should test as many body areas (arms [not hands], legs, trunk, face) as needed to accurately check for hemisensory loss. A score of 2, 'severe or total sensory loss', should only be given when a severe or total loss of sensation can be clearly demonstrated. Stuporous and aphasic patients will, therefore, probably score 1 or 0. The patient with brainstem stroke who has bilateral loss of sensation is scored 2. If the patient does not respond and is quadriplegic, score 2. Patients in a coma (item 1a=3) are automatically given a 2 on this item.	0= Normal; no sensory loss.<br>1= Mild-to-moderate sensory loss; patients feels pinprick is less sharp or is dull on the affected side; or there is a loss of superficial pain with pinprick, but patient is aware of being touched.<br>2= Severe to total sensory loss; patient is not aware of being touched in the face, arm and leg.
9. Best language: A great deal of information about comprehension will be obtained during the preceding sections of the examination. For this scale item, the patient is asked to describe what is happening in the attached picture, to name the items on the attached naming sheet and to read from the attached list of sentences. Comprehension is judged from responses here, as well as to all of the commands in the preceding general neurological exam. If visual loss interferes with the tests, ask the patient to identify objects placed in the hand, repeat, and produce speech. The intubated patient should be asked to write. The patient in a coma (item 1a=3) will automatically score 3 on this item. The examiner must choose a score for the patient with stupor or limited cooperation, but a score of 3 should be used only if the patient is mute and follows no one-step commands.	0= No aphasia; normal<br>1= Mild-to-moderate aphasia; some obvious loss of fluency or facility of comprehension, without significant limitation on ideas expressed or form of expression. Reduction of speech and/or comprehension, however, makes conservation about provided materials difficult or impossible. For example, in conversation about provided materials, examiner can identify picture or naming card content from patient's response.<br>2= Severe aphasia; all communication is through fragmentary expression; great need for inference, questioning, and guessing by the listener. Range of information that can be exchanged is limited; listener carries burden of communication. Examiner cannot identify materials provided from patient response.<br>3 = Mute, global aphasia: no usable speech or auditory comprehension.
10. Dysarthria: If patient is thought to be normal, an adequate sample of speech must be obtained by asking patient to read or repeat words from the attached list. If the patient has severe aphasia, the clarity of articulation of spontaneous speech can be rated. Only if patient is intubated or has other physical barriers to producing speech, the examiner should record the score as untestable (UN), and clearly write an explanation for this choice. Do not tell the patient why he or she is being tested.	0= Normal.<br>1= Mild-to-moderate dysarthria; patient slurs at least some words and, at worst, can be understood by some difficulty.<br>2= Severe dysarthria: patient's speech is so slurred as to be unintelligible in the absence of or out of proportion to any dysphasia, or is mute/anarthric.<br>UN = Intubated or other physical barrier.
11. Extinction and Inattention (formerly Neglect): Sufficient information to identify neglect may be obtained during the prior testing. If the patient has a severe visual loss preventing visual double simultaneous stimulation, and the cutaneous stimuli are normal, the score is normal. If the patient has aphasia but does appear to attend to both sides, the score is normal. The presence of visual spatial neglect or anosagnosia may also be taken as	0= No abnormality.<br>1= Visual, tactile, auditory, spatial, or personal inattention or extinction to bilateral simultaneous stimulation in one of the sensory modalities.<br>2= Profound hemi-inattention or extinction to more than one modality; does not recognize own hand or orients to only one side of space.

evidence of abnormality. Since the abnormality is scored only if present, the item is never untestable.	

Appendix table 4 EUROQOL 5D-5L

The EuroQoL 5-dimensions 5-level (EQ-5D-5L) questionnaire is a standardized measure of health outcome that has been used extensively in patients with stroke.

Under each heading, please tick the ONE box that best describes your health TODAY.

Mobility

I have no problems in walking about

I have slight problems in walking about

I have moderate problems in walking about

I have severe problems in walking about

I am unable to walk about

Self-care

I have no problems washing or dressing myself

I have slight problems washing or dressing myself

I have moderate problems washing or dressing myself

I have severe problems washing or dressing myself

I am unable to wash or dress myself

Usual activities (e.g. work, study, housework, family or leisure activities)

I have no problems doing my usual activities

I have slight problems doing my usual activities

I have moderate problems doing my usual activities

I have severe problems doing my usual activities

I am unable to do my usual activities

Pain/discomfort

I have no pain or discomfort

I have slight pain or discomfort

I have moderate pain or discomfort

I have severe pain or discomfort

I have extreme pain or discomfort

Anxiety/depression

I am not anxious or depressed

I am slightly anxious or depressed

I am moderately anxious or depressed

I am severely anxious or depressed

I am extremely anxious or depressed

Appendix table 5 Barthel Index

The Barthel index (BI) is an ordinal scale used to measure performance in 10 activities of daily living (ADL). Test scores range from 0 to 100, with higher scores indicating better performance in these activities.

Category	Scale definition
Feeding	0 = unable<br>5 = needs help cutting, spreading butter, etc., or requires modified diet<br>10 = independent
Bathing	0 = dependent<br>5 = independent (or in shower)
Grooming	0 = needs to help with personal care<br>5 = independent face/hair/teeth/shaving (implements provided)
Dressing	0 = dependent<br>5 = needs help but can do about half unaided<br>10 = independent (including buttons, zips, laces, etc.)
Bowels	0 = incontinent (or needs to be given enemas)<br>5 = occasional accident<br>10 = continent
Bladder	0 = incontinent, or catheterized and unable to manage alone<br>5 = occasional accident<br>10 = continent
Toilet use	0 = dependent<br>5 = needs some help, but can do something alone<br>10 = independent (on and off, dressing, wiping)
Transfers (bed to chair and back)	0 = unable, no sitting balance<br>5 = major help (one or two people, physical), can sit<br>10 = minor help (verbal or physical)<br>15 = independent
Mobility (on level surfaces)	0 = immobile or < 50 yards<br>5 = wheelchair independent, including corners, > 50 yards<br>10 = walks with help of one person (verbal or physical) > 50 yards<br>15 = independent (but may use any aid; for example, stick) > 50 yards
Stairs	0 = unable<br>5 = needs help (verbal, physical, carrying aid)<br>10 = independent

Guidelines

1. The index should be used as a record of what a patient does, not as a record of what a patient could do.
2. The main aim is to establish degree of independence from any help, physical or verbal, however minor and for whatever reason.
3. The need for supervision renders the patient not independent.
4. A patient's performance should be established using the best available evidence. Asking the patient, friends/relatives and nurses are the usual sources, but direct observation and common sense are also important. However, direct testing is not needed.
5. Usually the patient's performance over the preceding 24-48 hours is important, but occasionally

longer periods will be relevant.

6. Middle categories imply that the patient supplies over 50 per cent of the effort.
7. Use of aids to be independent is allowed.

Appendix table 6 Classification of Infarct in a New Territory

Classification based on size		Classification based on catheter manipulation across territory ostium
Type I	≤2 mm diffusion lesion (unidentifiable on NCCT)	Type A Catheter was manipulated past the ostium of the new territory (e.g. large ACA infarct in a patient with an initial M1 occlusion): greater likelihood that infarct is related to the procedure
Type II	>2 mm to ≤ 20 mm lesion (potentially difficult to identify on CT scan)	Type B Catheter was not manipulated past the ostium of the new territory (e.g. left PICA infarct in a patient with an initial right M1 occlusion): lower likelihood that infarct is related to procedure
Type III	Large (> 20 mm) infarct	

Appendix table 7 Description of Intracranial Hemorrhages

Class	Type	Description
1 Hemorrhagic transformation of infarcted brain tissue		
1a	HI1	Scattered small petechiae, no mass effect
1b	HI2	Confluent petechiae, no mass effect
1c	PH1	Hematoma within infarcted tissue, occupying <30%, no substantive mass effect
2 Intracerebral hemorrhage within and beyond infarcted brain tissue		
	PH2	Hematoma occupying 30% or more of the infarcted tissue, with obvious mass effect
3 Intracerebral hemorrhage outside the infarcted brain tissue or intracranial-extracerebral hemorrhage		
3a	rPH	Parenchymal hematoma remote from infarcted brain tissue
3b	IVH	Intraventricular hemorrhage
3c	SAH	Subarachnoid hemorrhage
3d	SDH	Subdural hemorrhage

Appendix table 8 Modified Arterial Occlusive Lesion Classification

Grade	Description
0	primary occlusive lesions remains same
1	debulking of thrombus without recanalization
2	partial or complete recanalization of the primary lesion with thrombus/occlusion in the distal vascular tree
3	complete recanalization of the primary occlusion with no thrombus in the vascular tree or beyond the primary occlusive lesions

Appendix table 9 Collateral Score

Category	Score	Description
None	0	Absent collaterals
Poor	1	Collaterals filling ≤50% of the occluded territory
Intermediate	2	Collaterals filing >50%, but <100% of the occluded territory
Good	3	Collaterals filling 100% of the occluded territory

Appendix table 10 Description of Subgroup Types and Definitions

	**Subgroups**	**Number of levels**	**Levels**
1	Quartiles of time from onset of symptoms to randomization	4	Min-Quartile 25%;<br>>Quartile 25%-Quartile 50%;<br>>Quartile 50%-Quartile 75%;<br>> Quartile 75%<br>(if applicable)
2	Quartiles of time from onset of symptoms to groin puncture	4	Min-Quartile 25%;<br>>Quartile 25%-Quartile 50%;<br>>Quartile 50%-Quartile 75%;<br>> Quartile 75%<br>(if applicable)
3	Quartiles of time from randomization to groin puncture	4	Min-Quartile 25%;<br>>Quartile 25%-Quartile 50%;<br>>Quartile 50%-Quartile 75%;<br>> Quartile 75%<br>(if applicable)
4	Quartiles of time from onset of symptoms to revascularization	4	Min-Quartile 25%;<br>>Quartile 25%-Quartile 50%;<br>>Quartile 50%-Quartile 75%;<br>> Quartile 75%<br>(if applicable)
5	Quartiles of time from randomization to revascularization	4	Min-Quartile 25%;<br>>Quartile 25%-Quartile 50%;<br>>Quartile 50%-Quartile 75%;<br>> Quartile 75%<br>(if applicable)
6	Ipsilateral extracranial carotid tandem lesion	2	Yes;<br>No
7	Occlusion location	3	ICA;<br>M1;<br>M2
8	Collaterals	2	Grade 0-1;<br>Grade 2-3
9	Large vessel occlusion due to different etiologies	3	Intracranial atherosclerosis;<br>Cardioembolism;<br>Others

STATISTICAL ANALYSIS PLAN(SAP)

Protocol Number:	CH01
Version Status (Draft /Final / Amendment):	2.0 / Amendment
Date:	21-Oct-2019

Direct Intra-arterial thrombectomy in order to Revascularize AIS patients with large vessel occlusion Efficiently in Chinese Tertiary hospitals: a Multicenter randomized clinical Trial (DIRECT-MT)

Study Statistician (CRO):

Sam Zhong

Shanghai KNOWLANDS MedPharm Consulting Co., Ltd.

Sponsor:

Changhai Hospital Affiliated to the Second Military Medical University

SIGNATURE PAGE

Direct Intra-arterial thrombectomy in order to Revascularize AIS patients with large vessel occlusion Efficiently in Chinese Tertiary hospitals: a Multicenter randomized clinical Trial (DIRECT-MT)

Study Statistician (CRO) Sam Zhong 2019-10-21

Sam Zhong
Shanghai KNOWLANDS
MedPharm Consulting Co., Ltd.

Date

SIGNATURE PAGE

Direct Intra-arterial thrombectomy in order to Revascularize AIS patients with large vessel occlusion Efficiently in Chinese Tertiary hospitals: a Multicenter randomized clinical Trial (DIRECT-MT)

Sponsor

Pengfei Yang	2019.10.22
Changhai Hospital Affiliated to the Second Military Medical University	Date

Revision history

Version	Date	Revision description
1.0	30-Nov-2018	Initial version, not applicable
2.0	21-Oct-2019	1. The SAP was revised to this current version primarily considering the protocol has been updated to version 3.0 (20-Aug-2019) from version 2.0 (31-Aug-2018).<br>2. In the current SAP, the primary analysis population was renamed to intention-to-treat population (ITT) instead of the full analysis set, while the same definition remained. And also elaborated several basic criteria for subjects entering PPS, see chapter 2.2.<br>3. Following the original statistical methodology strictly, this current SAP provided more wording details regarding statistical analysis variables and statistical methods, which involved demographic characteristics, efficacy, the definitive rules of subgroups, safety and analysis of quality of life as well, see chapter 2.<br>4. The current SAP added some subgroup analyses for primary efficacy endpoint, see chapter 2.3 for details.<br>5. A few document styles and formats in the current SAP template were adjusted as appropriate.

TABLE OF CONTENTS

TITLE PAGE **148**
TABLE OF CONTENTS **152**
LIST OF ABBREVIATIONS **154**
1 STUDY OVERVIEW **156**
1.1 STUDY DESIGN AND RANDOMIZATION 156
1.2 STUDY OBJECTIVES 157
1.3 STUDY OUTCOMES 157
1.4 SAMPLE SIZE CALCULATION 158
1.5 STUDY PROCEDURES 158
1.5.1 Procedures 159
2 STATISTICAL METHODOLOGY **159**
2.1 STATISTICAL VARIABLES 159
2.1.1 Background and demographic characteristics 159
2.1.2 Efficacy 160
2.1.3 Safety 161
2.1.4 Health economics 162
2.2 STATISTICAL ANALYSIS POPULATION 162
2.2.1 Intention-to-treat population 162
2.2.2 Per-protocol set 163
2.2.3 Subject disposition 163
2.3 STATISTICAL METHODS 164
2.3.1 Demography and baseline characteristics 164
2.3.2 Analysis of efficacy outcomes 164
2.3.3 Study treatment 166
2.3.4 Safety analysis 166
2.3.5 Analysis of quality of life 167
2.4 DATA PROCESSING CONVENTIONS 168
2.4.1 Definition of baseline 168
2.4.2 Missing data 168
2.4.3 Time window 168
2.4.4 Unscheduled visits 168
2.4.5 Centers pooling 168
3 CHANGES TO PLANNED ANALYSES FROM THE PROTOCOL **169**
4 INTERIM ANALYSIS **171**

5 STATISTICAL ANALYSIS SOFTWARE 172

6 REFERENCES 173

7 APPENDIX 174

Appendix table 1 Modified Rankin Scale 174
Appendix table 2 Extended Treatment In Cerebral Ischemia (eTICI) Scale 175
Appendix table 3 NIH Stroke Scale 176
Appendix table 4 EUROQOL 5D-5L 180
Appendix table 5 Barthel Index 181
Appendix table 6 Classification of Infarct in a New Territory 183
Appendix table 7 Description of Intracranial Hemorrhages 184
Appendix table 8 Modified Arterial Occlusive Lesion Classification 185
Appendix table 9 Collateral Score 186
Appendix table 10 Description of Subgroup Types and Definitions 187

LIST OF ABBREVIATIONS

Abbreviations	Definitions
AComA	Anterior communicating artery
ADL	Activities of Daily Living
AE	Adverse Event
AESI	Adverse Events of Special Interest
AIS	Acute Ischemic Stroke
AOL	Arterial occlusive lesion classification
APTT	Activated Partial Thromboplastin Time
ASPECTS	the Alberta Stroke Program Early CT Score
BI	Barthel Index
CI	Confidence interval
CONSORT	Consolidated Standards of Reporting Trials
cOR	Common odds ratio
CT	Computed tomography
CTA	Computed tomography angiography
CRF	Case Report Form
CRO	Contract Research Organization
DSA	Digital substraction angiography
DSMB	Data Safety Monitoring Board
EC	Ethics committee
eTICI	extended treatment in cerebral ischemia scale
EQ5D-5L	EuroQol-5 dimensions-5 level
EVT	Endovascular treatment
IAT	Intra-arterial treatment
INR	International normalized ratio
ITT	Intention-to-treat
IVT	Intravenous treatment
LOC	Level of consciousness
mAOL	Modified arterial occlusive lesion classification
MCA	Middle cerebral artery
MedDRA	Medical Dictionary for Drug Regulatory Activities

Abbreviations	Definitions
MRI	Magnetic resonance imaging
mRS	Modified Rankin scale
MT	Mechanical thrombectomy
NCCT	Non-contrast computed tomography
NIHSS	National Institute of Health stroke scale
PPS	Per-protocol set
PT	Preferred term
SAE	Serious adverse event
SAP	Statistical analysis plan
SAS	Statistical analysis system
SD	Standard deviation
sICH	Symptomatic intracerebral hemorrhage
SOC	System organ class

1 STUDY OVERVIEW

This Statistical Analysis Plan (SAP) is developed based on the most recent study protocol (Version 3.0, 20-Aug-2019) and Case Report Form (CRF, Version 1.4, 13-Nov-2018), and details the statistical analysis strategies and methods for the study.

This SAP predefines the statistical analysis population, variables and analysis methods before database lock to ensure the reliability of the study results.

1.1 STUDY DESIGN AND RANDOMIZATION

This is a multicenter prospective randomized clinical trial with open-label treatment and blinded outcome assessment (PROBE). The study will run for 4 years in intervention centers. Randomization will be stratified by center. The treatment allocation is 1:1 for:

- Direct IAT (MT),
- IVT followed by IAT (IVT plus MT)

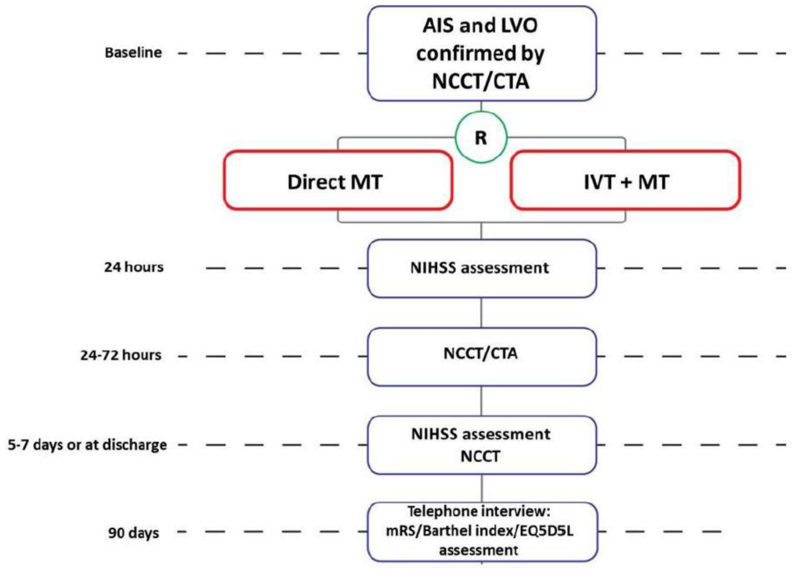

Figure 2 Patient flow in the trial

The intervention group will undergo immediate intra-arterial treatment (IAT) using a stent retriever, as recommended by the steering committee. Patients in the control group will receive alteplase intravenous treatment (IVT) (0.9 mg/kg with a maximum dose of 90 mg), followed by IAT using a stent retriever.
Local application (intra-arterial) of alteplase is allowed in any of the patients included in the DIRECT-MT if necessary. Patients pre-treated with IVT should not receive more than 30mg alteplase during intra-arterial treatment. Delivery of alteplase in shots of 5

mg in 5-10 minutes intervals is recommended. An equivalent dose of 400,000 U urokinase, delivered in shots of 50.000 - 100.000 U, in 5-10 minutes time intervals, is also accepted as escape medication in individual cases. If successful reperfusion (eTICI 2b-3) is not achieved in the direct MT group, IVT with 0.9 mg/kg may be initiated if the 4.5 hour window or maximum dose is not exceeded.

1.2 STUDY OBJECTIVES

The primary objective of this trial is to assess the effect of direct IAT compared with IVT followed by IAT, on functional outcome in patients with acute ischemic stroke (AIS), caused by an anterior circulation occlusion that is confirmed by Computed tomography angiography (CTA).
The secondary objective is to explore for superiority of direct IAT relative to IVT followed by IAT.
The tertiary objective is to assess the effect of direct IAT compared with IVT with IAT on neurological recovery (NIHSS), infarct size and occurrence of Symptomatic intracerebral hemorrhage (sICH).
The fourth objective is to collect thrombi and to analyze them with respect to their potential for treatment effect modification.

1.3 STUDY OUTCOMES

Primary outcome:
The primary outcome is the score on the modified Rankin Scale (mRS) (Table 1 in Appendix) at 90 days (± 14 days). The mRS is the preferred disability parameter for clinical trials in stroke. The mRS is an ordinal hierarchical scale incorporating six categories from 0 up to and including 5, and describes the range of disability encountered post stroke. "Death" is assigned a score of 6. Assessment of outcome on the mRS will be performed by outcome committee, blinded to the allocated and actually received treatment. Their assessment will be based on standardized reports of a telephone interview by trained research personnel who are not aware of treatment allocation.

Secondary outcomes:

- Death within 90 days (± 14 days)
- Pre-interventional recanalization
- extended treatment in cerebral ischemia scale (eTICI) score on final angiography of IAT (Table 2 in Appendix)
- Recanalization rate at 24-72 hours, assessed with CTA
- Score on the NIHSS at 24±6 hours and 5-7 days. (Table 3 in Appendix)

- Final infarct volume at 5-7 days. Final infarct volume will be assessed with the use of an automated, validated algorithm. Infarct size at day 5-7 will be compared with plain computed tomography (CT) and perfusion CT results (if available) at baseline.
- Dichotomized mRS of 0-1 vs. 2-6 at 90 days (±14 days)
- Dichotomized mRS of 0-2 vs. 3-6 at 90 days (±14 days)
- Dichotomized mRS of 0-3 vs. 4-6 at 90 days (±14 days)
- Score on the EuroQol-5 dimensions-5 level (EQ5D-5L) (Table 4 in Appendix) and Barthel index (BI) (Table 5 in Appendix) at 90 days (±14 days)

Safety outcomes:

- Hemorrhages according to the Heidelberg criteria [1]
- sICH scored according to the Heidelberg criteria [2]
- Embolization in new territory on angiography during IAT
- Occurrence of aneurysma spurium
- Occurrence of groin hematoma
- Infarction in new territory at 5-7 days (Table 6 in Appendix)
- Death from all causes within 90 days (±14 days)

1.4 SAMPLE SIZE CALCULATION

We based our estimations on the distribution of the mRS in the control group of the trial, which we derived from the intervention group of the MR CLEAN trial [3]: mRS 0: 3%; mRS 1: 9%; mRS 2: 21%; mRS 3: 18%; mRS 4: 22%; mRS 5: 6% and mRS 6: 21%. We assumed a favorable treatment effect with a common odds ratio (cOR) of 1.163, corresponding to a 4% absolute increase in the rate of mRS scores of 0-2. The main purpose is to demonstrate non-inferiority, that is, the lower limit of the two-side 95% confidence interval does not cross the pre-specified cOR non-inferiority Cutoff of 0.8. In a simulation with 5000 runs we computed the proportion of positive trials, for a given sample size. A sample size of 710 was determined to detect the pre-defined non-inferiority with a power of 80% and two-sided alpha of 0.05. Using covariate adjustment with at most 25%, a conservative 15% sample size reduction can be achieved, plus 5% dropout rate, leading to a final sample size of 636, 318 per arm.

1.5 STUDY PROCEDURES

Before starting the study, patients or their guardians must read and sign the informed consent approved by the current Ethics Committee (EC). All research steps should be

carried out within the time window specified in the study protocol.

All patients will undergo assessment of the NIHSS at baseline, 24±6 hours and 5-7 days, which is routine in clinical procedure. It will be carried out by certified assessors. Patients will undergo NCCT and CTA at baseline. After 24-72 hours, CTA is repeated to determine recanalization. At 5-7 days, patients will undergo non-contrast computed tomography (NCCT) to assess infarct size.

In addition, this trial also makes use of "waste material": retrieved thrombi during intervention. These thrombi will be stored in the participating study centers for follow-up analysis.

1.5.1 Procedures

All the procedures to be recorded are listed in Table 1.

Table 1 Procedures

Items	Procedures(includes but not limited to:)
Demography	Date of birth (based on valid identity documents), sex, age
Medical History	Disease history, smoking/alcohol drinking history, medications
Modified Rankin Scale	Disability level, ranging from 0~5
Glasgow coma Scale	Eye Opening, Best Verbal Response, Best Motor Response
Vital Signs	Systolic/diastolic blood pressure, heart rate, body temperature, height, weight
NIHSS	Level of consciousness (LOC), LOC Questions, LOC Commands, Best Gaze, Visual, Facial palsy, Motor arm, Motor leg, Limb ataxia, Sensory, Best language, Dysarthria, Extinction and Inattention
Laboratory tests	Serum glucose, Activated Partial Thromboplastin Time (APTT), International normalized ratio (INR), Thrombocyte count, Serum creatinine
eTICI	eTICI classification includes 0, 1, 2a, 2b, 2C and 3
EQ5D-5L score	Mobility, Self-Care, Usual-Activities, Pain/Discomfort, Anxiety/Depression
BARTHEL index	Feeding, Bathing, Grooming, dressing, Bowels, Bladder, Toilet use, Transfers(bed to chair and back), Mobility(on level surfaces), Stairs
Neuroimaging	CT, CTA, MRI and other imaging examinations

2 STATISTICAL METHODOLOGY

2.1 STATISTICAL VARIABLES

2.1.1 Background and demographic characteristics

The demographic and baseline information will include age, sex, medical history,

smoking history and medications used at home.

2.1.2 Efficacy

2.1.2.1 Primary efficacy variables

Primary efficacy outcome is mRS score change at 90 days (±14 days), which will be blindly evaluated by an independent Outcome Assessment Committee.

The mRS is the preferred disability parameter for clinical trials in stroke. The mRS is an ordinal hierarchical scale incorporating six categories from 0 up to and including 5, and describes the range of disability encountered post stroke. "Death" is assigned a score of 6 (Table 1 in Appendix).

2.1.2.2 Secondary efficacy variables

- Death within 90 days (±14 days)
- Pre-interventional recanalization
- eTICI score on final angiography of IAT. (Table 2 in Appendix)
- Recanalization rate at 24-72 hours, assessed with CTA
- Score on the NIHSS at 24±6 hours and 5-7 days. (Table 3 in Appendix)
- Final infarct volume at 5-7 days. Final infarct volume will be assessed with the use of an automated, validated algorithm. Infarct size at day 5-7 will be compared with plain CT and perfusion CT results (if available) at baseline.
- Dichotomized mRS of 0-1 vs. 2-6 at 90 days (±14 days)
- Dichotomized mRS of 0-2 vs. 3-6 at 90 days (±14 days)
- Dichotomized mRS of 0-3 vs. 4-6 at 90 days (±14 days)
- Dichotomized mRS of 0-4 vs. 5-6 at 90 days (±14 days)
- Dichotomized mRS of 0-5 vs. 6 at 90 days (±14 days)
- Score on the EQ5D-5L and Barthel index at 90 days (±14 days)

Pre-interventional recanalization：Recanalization rate (eTICI 2b, 2c or 3) before patients received mechanical intra-arterial treatment according to the DSA.

Recanalization rate at 24-72 hours: defined as the proportion of patients in whom recanalization as determined on 24-72 hours CTA is achieved.
eTICI score: eTICI assessment will be performed post IAT. The eTICI classification includes 0, 1, 2a, 2b, 2c and 3 (Table 2 in Appendix).

NIHSS score: The NIHSS is an ordinal hierarchical scale to evaluate the severity of stroke by assessing a patient's performance. Scores range from 0 to 42, with higher

scores indicating a more severe deficit (Table 3 in Appendix). NIHSS assessment will be performed at baseline, 24±6 hours post operation and 5-7 days post operation.

The EuroQoL 5-dimensions 5-level (EQ-5D-5L) questionnaire is a standardized measure of health outcome that has been used extensively in patients with stroke (Table 5 in Appendix). EQ5D-5L assessment will be performed at 90±14 days post operation.

The Barthel index (BI) is an ordinal scale used to measure performance in 10 activities of daily living (ADL). Test scores range from 0 to 100, with higher scores indicating better performance in these daily activities (Table 4 in Appendix). BI assessment will be performed at 90±14 days post operation.

2.1.3 Safety

2.1.3.1 Adverse events (AEs)

This study focused on the serious adverse events (SAEs) and Adverse Events of Special Interest (AESIs), and all reported SAEs will be blindly reviewed by an independent Adverse Event Committee.

All SAEs and AESIs will be coded using MedDRA 22.0 or higher, before database lock. MedDRA System Organ Class (SOC) and Preferred Term (PT) will be summarized.

Classification of serious adverse events

All SAEs will be classified as follows,

- Death
- Symptomatic intracranial hemorrhage
- De novo Ischemic Stroke
- Large or malignant middle cerebral artery (MCA) infarction
- Pneumonia (Aspiration and others)
- Contrast allergic reaction
- Major bleeding due to femoral artery access complications including groin hematoma, retroperitoneal hematoma
- Acute kidney injury
- Others

Adverse Events of Special Interest

Adverse events of special interest for this study include aspiration pneumonia and allergic contrast reactions.

2.1.3.2 Laboratory variables

Baseline laboratory tests will be conducted at screening visit, which include blood glucose (mmol/L), prothrombin time (sec), international standardized ratio, platelet count (*10^9), serum creatinine (umol/L).

2.1.3.3 Vital signs

At screening visit, the following vital signs will be measured: systolic blood pressure (mmHg), diastolic blood pressure (mmHg), heart rate (beat/min), body temperature (℃), height (cm), weight (kg).

2.1.3.4 Neuroimaging

CT and CTA will be performed at baseline and follow-up visit and the findings of which will be blindly evaluated blindly by an independent Imaging Committee (Core lab), including hyperdense, the Alberta Stroke Program Early CT Score (ASPECTS), another occlusion location of anterior circulation except target lesion, anterior communicating artery (AComA), intracranial hemorrhages (Table 7 in Appendix), midline shift present, target vessel stent placement, modified arterial occlusive lesion classification (mAOL, (Table 8 in Appendix)), vascular occlusion, etc.
The newly affected territory of the middle cerebral artery was graded by the systematic quantitative scoring system, e.g. ASPECTS. It will be performed at baseline visit and follow-up visit. ASPECTS is allotted 10 points, including caudate, lentiform, internal capsule, insular cortex, M1, M2, M3, M4, M5, M6. One point is subtracted for an area of early ischaemic change, such as focal swelling, or parenchymal hypoattenuation, for each of the defined regions. A score of 0 indicates diffuse ischaemia throughout the territory of the middle cerebral artery.

2.1.4 Health economics

None.

2.2 STATISTICAL ANALYSIS POPULATION

The analysis populations include intention-to-treat population (ITT) and per-protocol set (PPS) for this study.

2.2.1 Intention-to-treat population

All subjects who were randomized will be included in the intention-to-treat population (ITT) according to intention-to-treat principles, in which subjects will be analyzed according to the group assigned by randomization. ITT is the primary efficacy analysis set for this study.

2.2.2 Per-protocol set

Per-protocol set (PPS) is a subset of ITT, including all randomized subjects who have been treated in the study without major protocol deviations that may significantly impact the interpretation of efficacy results. Detailed protocol deviation criteria will be determined at the latest before database lock. PPS will be used for the primary efficacy outcome and safety analysis. Subjects entering PPS need to satisfy all the following basic criteria:

(1) Meet all the eligibility criteria specified in the study protocol;

(2) The subjects were randomized and received the assigned treatment, i.e.

−No IVT was administered before the intended endovascular treatment (EVT) in the intervention group (direct IAT group);

−IVT was administered before the intended EVT in patients in the control group.

(3) Underwent groin puncture, with exception of patients with clinical recovery precluding EVT (due to presumed recanalization before mechanical thrombectomy).

2.2.3 Subject disposition

The number and proportion of screened, randomized, treated and analyzed subjects will be provided. Where necessary, the CONSORT flow chart will be presented to describe the subject disposition in the statistical analysis report.

2.3 STATISTICAL METHODS

For normally distributed continuous data, the following statistics will be provided: number, mean, standard deviation (SD), minimum and maximum. For non-normally distributed continuous data, number, median, lower quartile (Q1), upper quartile (Q3) will be provided, unless otherwise stated. Categorical data will be summarized in terms of the number of patients and percentages.

For summary statistics, mean, standard deviation, median and quartiles will be reported to 1 more decimal place than the original data, while the 95% confidence interval (CI) will be reported to 2. Minimum and maximum values will be reported to the same number of significant digits as the original data. In the frequency table, the percentages will keep 1 decimal, the p values keep 4 decimal or displayed as "<0.0001".

2.3.1 Demography and baseline characteristics

Demography and baseline characteristics will be statistically summarized by treatment group.

In addition, the medical history, smoking history, drug treatment history and other information will be summarized. Data listings will be provided where necessary.

2.3.2 Analysis of efficacy outcomes

All efficacy data analyses will be based on ITT and for primary endpoint PPS will also be used.

2.3.2.1 Primary efficacy outcome

The primary effect parameter is the common odds ratio, which will be estimated by ordinal logistic regression (proportional odds model), which represent the shift on the full distribution of the modified Rankin Scale at 90±14 days. Estimations will be adjusted by known prognostic variables such as age (median), pre-stroke mRS (continuous), time from symptom onset to randomization ("<=Q1", ">Q1, <=Q2", ">Q2, <=Q3", ">Q3"), stroke severity (NIHSS, median) and collaterals (Grade 0-1, Grade 2-3). Adjusted and unadjusted estimations and their corresponding 95% confidence intervals will be reported. To assess non-inferiority of direct MT compared to IVT with MT, we will assess whether the 95% CI lower bound of the adjusted common odds ratio cross our pre-specified non-inferiority boundary (0.8).

The following SAS procedure will be used for ordered logistic regression analysis (proportional odds model):

```
Proc    logistic data=XXX;
Class TRT FactorA …;
Model mRS90= TRT AGE FactorA …;
```

```
Run;
```

2.3.2.2 Secondary efficacy outcome

Continuous secondary efficacy outcomes are mainly infarct size at 5-7 days after operation and recanalization rate before intervention as well. Analysis for these outcomes will be mainly based on statistical descriptions. Where necessary, analysis of variance or corresponding non-parametric test will be used for between-group comparisons. If applicable, the linear regression analysis will be used with adjustment for the same covariate variables as the primary outcome analysis. When deemed necessary, log or other common transformation of non-normal distribution will be used.

Categorical secondary outcomes include mortality at 90 days after operation, recanalization rate at 24-72 hours, dichotomized mRS score at 90 days after operation (0-1 vs. 2-6, 0-2 vs. 3-6, 0-3 vs. 4-6), successful recanalization before and after Mechanical thrombectomy (MT), and eTICI score at MT final angiography. Chi-square test will be used for comparison between the two groups, or Fisher's exact test will be used for comparison when applicable. The categorical secondary outcomes will be analyzed by logistic or ordered regression analysis to provide a common odds ratio and its confidence interval, if applicable. The adjustment method is the same as that in the primary outcome analysis.

2.3.2.3 Subgroup analysis

Pre-specified subgroup analysis will be performed by examining the interaction between specific baseline characteristics and treatment. Baseline grouping factors for subgroup analysis include, but are not limited to:

- Age
- Baseline NIHSS
- Quartiles of time from onset of symptoms to randomization
- Quartiles of time from onset of symptoms to groin puncture
- Quartiles of time from randomization to groin puncture
- Quartiles of time from onset of symptoms to revascularization
- Quartiles of time from randomization to revascularization
- Ipsilateral extracranial carotid tandem lesion
- Occlusion location
- Collaterals (Table 9 in Appendix)
- Large vessel occlusion due to different etiologies
- Thrombus perviousness

– Thrombus density

See the detailed description of subgroup types and definitions in Appendix Table 10.

2.3.2.4 Multiplicity

This study does not consider multiplicity issues and therefore does not adjust significance levels based on multiplicity tests, unless specified otherwise.

2.3.3 Study treatment

2.3.3.1 Intravenous alteplase therapy

Intravenous alteplase therapy will be summarized (only applied to IVT plus MT group), including whether IVT is performed, planned alteplase dose (mg) and residual alteplase volume (ml).

2.3.3.2 Intra-arterial treatment

A descriptive summary of intra-arterial therapy will be provided according to the treatment groups, including anesthesia management, pre-treatment, treatment, eTICI score as determined by final angiography, thrombectomy, intra-operative non-study drugs, stent implantation/balloon dilatation at the intracranial atherosclerosis occlusion site.

2.3.3.3 Digital substraction angiography (DSA)

The results of DSA will be blindly evaluated by the independent Imaging Committee (Core lab) , including but not limited to: ipsilateral extracranial carotid tandem lesion, intracranial arterial occlusions, another occlusion location of anterior circulation except target lesion, arterial occlusive lesion classification (AOL) and intracranial atherosclerosis occlusion, will be summarized according to the treatment groups.

2.3.4 Safety analysis

In this study, the safety analysis will be mainly based on statistical description. All the analyses will be based on PPS.

2.3.4.1 Analysis of adverse events (AEs)

The number and percentage of subjects who had at least one serious adverse event, classification of serious adverse event, adverse events of special interest and classification of adverse events of special interest from study will be provided.

- All SAEs will be summarized by SOC and PT;
- All AESIs will be summarized by SOC and PT;

2.3.4.2 Clinical laboratory data analysis

Laboratory tests included blood sugar, prothrombin time, international standardized

ratio, platelet count and serum creatinine.

For continuous laboratory parameters, summary statistics, including number of subjects, mean, standard deviation, median, minimum and maximum will be provided for observed values for each parameter.

If a lab test result is recorded as "<10", then it will be summarized as a value of "5", if applicable; and likewise, ">10" will be summarized as "10".

2.3.4.3 Analysis of vital signs

Summaries of vital signs parameters will be presented by treatment group, using summary statistics, including number of subjects, mean, standard deviation, median, minimum and maximum for observed values for each parameter.

2.3.4.4 Analysis of neuroimaging

ASPECTS (0-10) and change from baseline are continuous variables and will be presented with summary statistics. The frequency table of each point will also be provided by treatment groups.

Other results of CT and CTA will be summarized using frequency table by treatment groups (if necessary).

2.3.5 Analysis of quality of life

2.3.5.1 NIHSS score

NIHSS (0-42) score and change from baseline are continuous variables and will be presented with summary statistics, including number of subjects, mean, standard deviation, median, minimum and maximum, by treatment groups and by visits. Repeated measures of variance analysis will be used to explore the impact of treatment grouping visits and NIHSS baseline levels.

2.3.5.2 EQ5D-5L score

The frequency and percentage of EQ5D-5L scale will be summarized according to each dimension. If necessary, Chi-square test will be used for comparison between the two groups, or Fisher's exact test will be used for comparison when applicable.

2.3.5.3 Barthel index

Barthel score is a continuous variable (0-100) and will be summarized using number, mean, standard deviation, median, minimum and maximum, by treatment groups. The frequency table of each class level will also be provided.

2.4 DATA PROCESSING CONVENTIONS

2.4.1 Definition of baseline

In this study, baseline values are defined as those data collected before intervention (screening visit). When multiple data collections occur during the baseline period, the final data shall prevail in principle, unless explicitly stated.

2.4.2 Missing data

We will report proportions of missing values for all collected variables where needed.
Baseline characteristics missing data will be imputed by regression interpolation as appropriate.
If there is a large number of missing data on efficacy and safety, an evaluation on the missing data should be conducted before analysis, and will propose and determine the solution before database lock.
For patients who died within the study period, the worst scores will be assigned for all not-assessed clinical outcome measures in their analyses, as follows Table 2.

Table 2 The worst scores of clinical outcomes

Clinical outcomes	The worst scores
mRS	6
NIHSS	42
The Barthel index	0

2.4.3 Time window

Not applicable.

2.4.4 Unscheduled visits

Not applicable.

2.4.5 Centers pooling

Unless specifically specified, this study will not consider the center effect, so it will not pool and analyze the data of each study center.

3 CHANGES TO PLANNED ANALYSES FROM THE PROTOCOL

Protocol version (Date)	Major Changes to Planned Analyses from the Protocol
Version 2.0 (31-Aug-2018)	● Recalculated the sample size according to the modified good outcome (Section 4.5 of the protocol)<br><br>After revision:<br><br>*We assumed a favorable treatment effect with a common odds ratio (cOR) of 1.163, corresponding to a 4% absolute increase in the rate of mRS scores of 0-2. The main aim of the trial is to demonstrate non-inferiority. To do so, the lower limit of the two-sided 95% confidence interval of the cOR should not cross the pre-specified non-inferiority boundary of 0.8.*<br><br>*In a Monte Carlo simulation with 5000 runs we computed the proportion of positive trials, for a given sample size. A sample size of 710 was determined to detect the pre-defined non-inferiority with a power of 80% and two-sided alpha of 0.05. Using covariate adjustment with at most 25%, a conservative 15% sample size reduction can be achieved, plus 5% dropout rate, leading to a final sample size of 636, 318 per arm.*<br><br>Before revision:<br><br>*We based our estimations on the distribution of the modified Rankin Scale (mRS) in the control group of the trial, which we derived from the intervention group of the MR CLEAN trial (9): mRS 0: 3%; mRS 1: 9%; mRS 2: 21%; mRS 3: 18%; mRS 4: 22%; mRS 5: 6% and mRS 6: 21%. We assumed a favorable treatment effect with a common odds ratio (cOR) of 1.54, which corresponds to an absolute risk difference of having a score on the modified Rankin Scale of 0-2 of approximately 8%. The main purpose is to demonstrate non-inferiority, that is, the lower limit of the 95% confidence interval does not cross the pre-specified cOR non-inferiority Cutoff of 0.8. In a simulation with 5000 runs we computed the proportion of positive trials, for a given sample size. This yielded a sample size of 680, providing 99% power to detect a true treatment effect, with two-sided alpha =0.05. In the analysis we will use covariate adjustment, which reduces the required sample size with 25% (28, 29). Therefore, the aim is to include 540 patients, 270 in each group of the trial, considering a dropout rate of 5%.*<br><br>● Revised the interim analyses plan (Section 9.4 of the

	protocol)<br>After revision:<br>*DSMB plans to conduct two interim analyses to evaluate the treatment effect and the incidence of adverse reactions according to the procedure at the end of the 90-day follow-up of 1/3 and 2/3 subjects, respectively.*<br><br>Before revision:<br>*The DSMB will meet frequently, at least annually or after inclusion of the next 100 patients (whichever comes first) and assess the occurrence of adverse events by center and by procedure.*<br><br>● Modified Study committees member list<br>Data Safety Monitoring Board, Outcome Assessment Committee and Adverse Event Adjudication Committee added.
Version 3.0 (20-Aug-2019)	● Subgroup analysis (Section 10.2 of the protocol)<br>One subgroup added: *Large vessel occlusion due to different etiologies*<br><br>● Modified Study committees member list<br>Imaging Assessment Committee added.

4 INTERIM ANALYSIS

A formal interim analysis is planned.
In order to increase the safety of the intervention, the trial will be monitored by an independent Data Safety Monitoring Board (DSMB). The DSMB will be chaired by a neurologist, and include a neuro-interventionist and an independent methodologist/ statistician. The DSMB plans to conduct two interim analyses to evaluate the treatment effect and the incidence of adverse reactions according to the procedure at the end of the 90-day follow-up of 1/3 and 2/3 subjects, respectively. During the period of patient enrollment into the study, interim analyses of mortality and of any other information that is available on major outcomes (including serious adverse events believed to be due to treatment) will be supplied, in strict confidence, to the chairman of the DSMB, along with any other analyses that the DSMB may request. In the light of these analyses, DSMB will advise the chairman of the Steering Committee if, in their view, the randomized comparisons in DIRECT-MT have provided both (i) "proof beyond reasonable doubt" that for all, or for some specific types of patients, one particular treatment is clearly indicated or clearly contraindicated in terms of a net difference in outcome, and (ii) evidence that might reasonably be expected to materially influence patient management. Appropriate criteria of proof beyond reasonable doubt cannot be specified precisely, but a difference of at least 3 standard deviations in an interim analysis of a major outcome may be needed to justify halting, or modifying, the study prematurely. This criterion has the practical advantage that the number of interim analyses is of little importance.
The advice(s) of the DSMB will be sent to the sponsor of the study by the chair of the steering committee. Should the sponsor decide not to fully implement the advice of the DSMB, the sponsor will send the advice to the EC, including a note to substantiate why (part of) the advice of the DSMB will not be followed.

5 STATISTICAL ANALYSIS SOFTWARE

All statistical analysis and summary will be carried out using SAS 9.2 or higher version in this study. Software R 3.3.1 or higher version will be used for drawing plots if applicable.

6 REFERENCES

[1] Mahoney FI, Barthel DW. Functional Evaluation: The Barthel Index. Md State Med J. 1965;14:61-5.

[2] von Kummer R, Broderick JP, Campbell BC, Demchuk A, Goyal M, Hill MD, et al. The Heidelberg Bleeding Classification: Classification of Bleeding Events After Ischemic Stroke and Reperfusion Therapy. Stroke. 2015;46(10):2981-6.

[3] Berkhemer OA, Fransen PS, Beumer D, van den BergL.A, Lingsma HF, Yoo AJ, et al. A randomized trial of intraarterial treatment for acute ischemic stroke. N Engl J Med. 2015372(1): 11-20

7 APPENDIX

Appendix table 1 Modified Rankin Scale

The modified Rankin Scale (mRS) is an ordinal hierarchical scale ranging from 0 to 5, with higher scores indicating more severe disability. A score of 6 has been added to signify death.

Category	Short description	Long description
0	No symptoms	No symptoms
1	Symptoms, no disability	Minor symptoms that do not interfere with lifestyle
2	Slight disability	Slight disability, symptoms that lead to some restriction in lifestyle, but do not interfere with the patient's capacity to look after himself.
3	Moderate disability	Moderate disability, symptoms that significantly restrict lifestyle and prevent totally independent existence
4	Moderately severe disability	Moderately severe disability, symptoms that clearly prevent independent existence though not needing constant attention
5	Severe disability	Severe disability, totally dependent patient requiring constant attention day and night.
6	Death	Death

Appendix table 2 Extended Treatment In Cerebral Ischemia (eTICI) Scale

eTICI grade	Short description	Long description
0	No perfusion	No antegrade flow beyond the point of occlusion
1	Limited reperfusion	Antegrade reperfusion past the initial occlusion, but limited distal branch filling with little or slow distal reperfusion
2a	<50% reperfusion	Antegrade reperfusion of less than half of the occluded target artery previously ischemic territory (eg, in 1 major division of the MCA and its territory)
2b	≥50% and <90% reperfusion	Antegrade reperfusion of more than half of the previously occluded target artery ischemic territory (eg, in 2 major divisions of the MCA and its territories)
2c	≥90% reperfusion	Near complete antegrade reperfusion of the previously occluded target artery ischemic territory, except for slow flow or distal emboli in a few distal cortical vessels
3	100% reperfusion	Complete antegrade reperfusion of the previously occluded target artery ischemic territory, with absence of visualized occlusion in all distal branches

Appendix table 3 NIH Stroke Scale

The NIHSS is an ordinal hierarchical scale to evaluate the severity of stroke by assessing a patient's performance. (23) Scores range from 0 to 42, with higher scores indicating a more severe deficit. Administer stroke scale items in the order listed. Record performance in each category after each subscale exam. Do not go back and change scores. Follow directions provided for each exam technique. Scores should reflect what the patient does, not what the clinician thinks the patient can do. The clinician should record answers while administering the exam and work quickly. Except where indicated, the patient should not be coached (i.e. repeated requests to patient to make a special effort).

Instructions	Scale definition
1a. Level of consciousness. The investigator must choose a response if a full evaluation is prevented by such obstacles as an endotracheal tube, language barrier, orotracheal trauma/bandages. A 3 is scored only if the patient makes no movement (other than reflexive posturing) in response to noxious stimulation.	0 = Alert; keenly responsive.<br>1 = Not alert; but arousable by minor stimulation to obey, answer, or respond.<br>2 = Not alert; required repeated stimulation to attend, or is obtunded and requires strong or painful stimulation to make movements (not stereotyped).<br>3 = Responds only with reflex motor or autonomic effects or totally unresponsive, flaccid and areflexic.
1b. LOC Questions: The patient is asked the month and his/her age. The answer must be correct – there is not partial credit for being close. Phasic and stuporous patients who do not comprehend the questions will score 2. Patients unable to speak because of endotracheal intubation, orotracheal trauma, severe dysarthria from any cause, language barrier, or any other problem not secondary to aphasia are given a 1. It is important that only the initial answer be graded and that the examiners not "help" the patient with verbal or non-verbal clues.	0 = Answers both questions correctly.<br>1 = Answers one question correctly.<br>2 = Answers neither question correctly.
1c. LOC Commands: The patient is asked to open and close the eyes and then to grip and release the non-paretic hand. Substitute another one step command if the hand cannot be used. Credit is given if an unequivocal attempt is made but not completed due to weakness. If the patient does not respond to command, the task should be demonstrated to him or her (pantomime), and the result scored<br>(i.e. follows none, one or two commands). Patients with trauma, amputation, or other physical impediments should be given suitable one-step commands. Only the first attempt is scored.	0 = Performs both tasks correctly.<br>1 = Performs one task correctly.<br>2 = Performs neither task correctly.
2. Best Gaze: Only horizontal eye movements will be tested. Voluntary or reflexive (oculocephalic) eye movements will be scored, but caloric testing is not done. If the patient has a conjugate deviation of the eyes that can be overcome by voluntary or reflexive activity, the score will be a 1. If a patient has an isolated peripheral nerve paresis (CN III, IV or VI), score a 1. Gaze is testable in all aphasic patients. Patients with ocular trauma, bandages, preexisting blindness, or other disorder of visual acuity or fields should be tested with reflexive movements, and a choice made by the investigator. Establishing eye contact and then moving about the patient from side to side will occasionally clarify the presence of a partial gaze palsy.	0= Normal.<br>1= Partial gaze palsy; gaze is abnormal in one or both eyes, but forced deviation or total gaze paresis is not present.<br>2= Forced deviation; or total gaze paresis not overcome by the oculocephalic maneuver.

3. Visual: Visual fields (upper and lower quadrants) are tested by confrontation, using finger counting or visual threat, as appropriate. Patients may be encouraged, but if they look at the side of the moving finger appropriately, this can be scored as normal. If there is unilateral blindness or enucleation, visual fields in the remaining eye are scored. Score 1 only if a clear-cut asymmetry, including quadrantanopia, is found. If patient is blind from any cause, score 3.<br>Double simultaneous stimulation is performed in this case.<br>If there is extinction, the patient receives a 1, and the results are used to respond to item 11.	0= No visual loss.<br>1= Partial hemianopia.<br>2= Complete hemianopia.<br>3= Bilateral hemianopia (blind including cortical blindness)
4. Facial palsy: Ask or use pantomime to encourage the patient to show teeth or raise eyebrows and close eyes. Score symmetry of grimace in response to noxious stimuli in the poorly response or non-comprehending patient. If facial trauma/bandages, orotracheal tube, tape or other physical barriers obscure the face, these should be removed to the extent possible.	0 = Normal symmetrical movements.<br>1= Minor paralysis (flattened nasolabial fold, asymmetry on smiling)<br>2= Partial paralysis (total or near-total paralysis of lower face)<br>3= Complete paralysis of one or both sides (absence of facial movement in the upper and lower face).
5. Motor arm: The limb is placed in the appropriate position: extend the arms (palms down) 90 degrees (if sitting) or 45 degrees (if supine). Drift is scored if the arm falls before 10 seconds. The aphasic patient is encouraged using urgency in the voice and pantomime, but not noxious stimulation. Each limb is tested in turn, beginning with the non-paretic arm. Only in the case of amputation or joint fusion at the shoulder, the examiner should record the score as untestable (UN), and clearly write the explanation for this choice.	0= No drift; limb holds 90 (or 45) degrees for full 10 seconds.<br>1= Drift; limb holds 90 (or 45) degrees, but drifts down before full 10 seconds; does not hit bed or other support.<br>2= Some effort against gravity; limb cannot get to or maintain (if cued) 90 (or 45) degrees, drifts down to bed, but has some effort against gravity.<br>3= No effort against gravity; limb falls.<br>4= No movement.<br>UN = Amputation or joint fusion: explain:<br>5a = Left Arm.<br>5b = Right arm.
6. Motor leg: The limb is placed in the appropriate position: hold the leg at 30 degrees (always tested supine). Drift is scored if the leg falls before 5 seconds. The aphasic patient is encouraged using urgency in the voice and pantomime, but not noxious stimulation. Each limb is tested in turn, beginning with the non-paretic leg. Only in the case of amputation or joint fusion at the hip, the examiner should record the score as untestable (UN), and clearly write the explanation for this choice.	0= No drift; leg holds 30-degree position for full 5 seconds.<br>1= Drift; leg falls by the end of the 5-second period but does not hit bed.<br>2= Some effort against gravity; leg falls to bed by 5 seconds, but has some effort against gravity.<br>3= No effort against gravity; leg falls to bed immediately.<br>4= No movement.<br>UN = Amputation or joint fusion: explain:<br>6a. Left Leg<br>6b. Right Leg.
7. Limb ataxia: This item is aimed at finding evidence of a unilateral cerebellar lesion. Test with eyes open. In case of visual defect, ensure testing is done in intact visual field. The finger-nose-finger and heel-shin tests are performed on both sides, and ataxia is scored only if present out of proportion to weakness. Ataxia is absent in the patient who cannot understand or is paralyzed. Only in the case of amputation or joint fusion, the examiner should record the score as untestable (UN), and	0= Absent.<br>1= Present in one limb.<br>2= Present in two limbs.<br>UN = Amputation or joint fusion: explain:

clearly write the explanation for this choice. In case of blindness, test by having the patient touch nose from extended arm position.	
8. Sensory: Sensation or grimace to pinprick when tested, or withdrawal from noxious stimulus in the obtunded or aphasic patient. Only sensory loss attributed to stroke is scored as abnormal and the examiner should test as many body areas (arms [not hands], legs, trunk, face) as needed to accurately check for hemisensory loss. A score of 2, 'severe or total sensory loss', should only be given when a severe or total loss of sensation can be clearly demonstrated. Stuporous and aphasic patients will, therefore, probably score 1 or 0. The patient with brainstem stroke who has bilateral loss of sensation is scored 2. If the patient does not respond and is quadriplegic, score 2. Patients in a coma (item 1a=3) are automatically given a 2 on this item.	0= Normal; no sensory loss.<br>1= Mild-to-moderate sensory loss; patients feels pinprick is less sharp or is dull on the affected side; or there is a loss of superficial pain with pinprick, but patient is aware of being touched.<br>2= Severe to total sensory loss; patient is not aware of being touched in the face, arm and leg.
9. Best language: A great deal of information about comprehension will be obtained during the preceding sections of the examination. For this scale item, the patient is asked to describe what is happening in the attached picture, to name the items on the attached naming sheet and to read from the attached list of sentences. Comprehension is judged from responses here, as well as to all of the commands in the preceding general neurological exam. If visual loss interferes with the tests, ask the patient to identify objects placed in the hand, repeat, and produce speech. The intubated patient should be asked to write. The patient in a coma (item 1a=3) will automatically score 3 on this item. The examiner must choose a score for the patient with stupor or limited cooperation, but a score of 3 should be used only if the patient is mute and follows no one-step commands.	0= No aphasia; normal<br>1= Mild-to-moderate aphasia; some obvious loss of fluency or facility of comprehension, without significant limitation on ideas expressed or form of expression. Reduction of speech and/or comprehension, however, makes conservation about provided materials difficult or impossible. For example, in conversation about provided materials, examiner can identify picture or naming card content from patient's response.<br>2= Severe aphasia; all communication is through fragmentary expression; great need for inference, questioning, and guessing by the listener. Range of information that can be exchanged is limited; listener carries burden of communication. Examiner cannot identify materials provided from patient response.<br>3 = Mute, global aphasia: no usable speech or auditory comprehension.
10. Dysarthria: If patient is thought to be normal, an adequate sample of speech must be obtained by asking patient to read or repeat words from the attached list. If the patient has severe aphasia, the clarity of articulation of spontaneous speech can be rated. Only if patient is intubated or has other physical barriers to producing speech, the examiner should record the score as untestable (UN), and clearly write an explanation for this choice. Do not tell the patient why he or she is being tested.	0= Normal.<br>1= Mild-to-moderate dysarthria; patient slurs at least some words and, at worst, can be understood by some difficulty.<br>2= Severe dysarthria: patient's speech is so slurred as to be unintelligible in the absence of or out of proportion to any dysphasia, or is mute/anarthric.<br>UN = Intubated or other physical barrier.
11. Extinction and Inattention (formerly Neglect): Sufficient information to identify neglect may be obtained during the prior testing. If the patient has a severe visual loss preventing visual double simultaneous stimulation, and the cutaneous stimuli are normal, the score is normal. If the patient has aphasia but does appear to attend to both sides, the score is normal. The presence of visual spatial neglect or anosagnosia may also be taken as evidence of abnormality. Since the abnormality is scored only if present, the item is never untestable.	0= No abnormality.<br>1= Visual, tactile, auditory, spatial, or personal inattention or extinction to bilateral simultaneous stimulation in one of the sensory modalities.<br>2= Profound hemi-inattention or extinction to more than one modality; does not recognize own hand or orients to only one side of space.

Appendix table 4 EUROQOL 5D-5L

The EuroQoL 5-dimensions 5-level (EQ-5D-5L) questionnaire is a standardized measure of health outcome that has been used extensively in patients with stroke.

Under each heading, please tick the ONE box that best describes your health TODAY.

Mobility

I have no problems in walking about

I have slight problems in walking about

I have moderate problems in walking about

I have severe problems in walking about

I am unable to walk about

Self-care

I have no problems washing or dressing myself

I have slight problems washing or dressing myself

I have moderate problems washing or dressing myself

I have severe problems washing or dressing myself

I am unable to wash or dress myself

Usual activities (e.g. work, study, housework, family or leisure activities)

I have no problems doing my usual activities

I have slight problems doing my usual activities

I have moderate problems doing my usual activities

I have severe problems doing my usual activities

I am unable to do my usual activities

Pain/discomfort

I have no pain or discomfort

I have slight pain or discomfort

I have moderate pain or discomfort

I have severe pain or discomfort

I have extreme pain or discomfort

Anxiety/depression

I am not anxious or depressed

I am slightly anxious or depressed

I am moderately anxious or depressed

I am severely anxious or depressed

I am extremely anxious or depressed

Appendix table 5 Barthel Index

The Barthel index (BI) is an ordinal scale used to measure performance in 10 activities of daily living (ADL). Test scores range from 0 to 100, with higher scores indicating better performance in these activities.

Category	Scale definition
Feeding	0 = unable<br>5 = needs help cutting, spreading butter, etc., or requires modified diet<br>10 = independent
Bathing	0 = dependent<br>5 = independent (or in shower)
Grooming	0 = needs to help with personal care<br>5 = independent face/hair/teeth/shaving (implements provided)
Dressing	0 = dependent<br>5 = needs help but can do about half unaided<br>10 = independent (including buttons, zips, laces, etc.)
Bowels	0 = incontinent (or needs to be given enemas)<br>5 = occasional accident<br>10 = continent
Bladder	0 = incontinent, or catheterized and unable to manage alone<br>5 = occasional accident<br>10 = continent
Toilet use	0 = dependent<br>5 = needs some help, but can do something alone<br>10 = independent (on and off, dressing, wiping)
Transfers (bed to chair and back)	0 = unable, no sitting balance<br>5 = major help (one or two people, physical), can sit<br>10 = minor help (verbal or physical)<br>15 = independent
Mobility (on level surfaces)	0 = immobile or < 50 yards<br>5 = wheelchair independent, including corners, > 50 yards<br>10 = walks with help of one person (verbal or physical) > 50 yards<br>15 = independent (but may use any aid; for example, stick) > 50 yards
Stairs	0 = unable<br>5 = needs help (verbal, physical, carrying aid)<br>10 = independent

Guidelines

1. The index should be used as a record of what a patient does, not as a record of what a patient could do.
2. The main aim is to establish degree of independence from any help, physical or verbal, however minor and for whatever reason.
3. The need for supervision renders the patient not independent.
4. A patient's performance should be established using the best available evidence. Asking the patient, friends/relatives and nurses are the usual sources, but direct observation and common sense are also important. However, direct testing is not needed.
5. Usually the patient's performance over the preceding 24-48 hours is important, but occasionally

longer periods will be relevant.
6. Middle categories imply that the patient supplies over 50 per cent of the effort.
7. Use of aids to be independent is allowed.

Appendix table 6 Classification of Infarct in a New Territory

Classification based on size		Classification based on catheter manipulation across territory ostium
Type I	≤2 mm diffusion lesion (unidentifiable on NCCT)	Type A Catheter was manipulated past the ostium of the new territory (e.g. large ACA infarct in a patient with an initial M1 occlusion): greater likelihood that infarct is related to the procedure<br>Type B Catheter was not manipulated past the ostium of the new territory (e.g. left PICA infarct in a patient with an initial right M1 occlusion): lower likelihood that infarct is related to procedure
Type II	>2 mm to ≤ 20 mm lesion (potentially difficult to identify on CT scan)	
Type III	Large (> 20 mm) infarct	

Appendix table 7 Description of Intracranial Hemorrhages

Class	Type	Description
1 Hemorrhagic transformation of infarcted brain tissue		
1a	HI1	Scattered small petechiae, no mass effect
1b	HI2	Confluent petechiae, no mass effect
1c	PH1	Hematoma within infarcted tissue, occupying <30%, no substantive mass effect
2 Intracerebral hemorrhage within and beyond infarcted brain tissue		
	PH2	Hematoma occupying 30% or more of the infarcted tissue, with obvious mass effect
3 Intracerebral hemorrhage outside the infarcted brain tissue or intracranial-extracerebral hemorrhage		
3a	rPH	Parenchymal hematoma remote from infarcted brain tissue
3b	IVH	Intraventricular hemorrhage
3c	SAH	Subarachnoid hemorrhage
3d	SDH	Subdural hemorrhage

Appendix table 8 Modified Arterial Occlusive Lesion Classification

Grade	Description
0	primary occlusive lesions remains same
1	debulking of thrombus without recanalization
2	partial or complete recanalization of the primary lesion with thrombus/occlusion in the distal vascular tree
3	complete recanalization of the primary occlusion with no thrombus in the vascular tree or beyond the primary occlusive lesions

Appendix table 9 Collateral Score

Category	Score	Description
None	0	Absent collaterals
Poor	1	Collaterals filling ≤50% of the occluded territory
Intermediate	2	Collaterals filing >50%, but <100% of the occluded territory
Good	3	Collaterals filling 100% of the occluded territory

Appendix table 10 Description of Subgroup Types and Definitions

	**Subgroups**	**Number of levels**	**Levels**
1	Age (Years)	3	18-60;<br>60-80;<br>⩾80
2	Baseline NIHSS	3	2-15;<br>16-19;<br>⩾20
3	Quartiles of time from onset of symptoms to randomization	4	Min-Quartile 25%;<br>>Quartile 25%-Quartile 50%;<br>>Quartile 50%-Quartile 75%;<br>> Quartile 75%<br>(if applicable)
4	Quartiles of time from onset of symptoms to groin puncture	4	Min-Quartile 25%;<br>>Quartile 25%-Quartile 50%;<br>>Quartile 50%-Quartile 75%;<br>> Quartile 75%<br>(if applicable)
5	Quartiles of time from randomization to groin puncture	4	Min-Quartile 25%;<br>>Quartile 25%-Quartile 50%;<br>>Quartile 50%-Quartile 75%;<br>> Quartile 75%<br>(if applicable)
6	Quartiles of time from onset of symptoms to revascularization	4	Min-Quartile 25%;<br>>Quartile 25%-Quartile 50%;<br>>Quartile 50%-Quartile 75%;<br>> Quartile 75%<br>(if applicable)
7	Quartiles of time from randomization to revascularization	4	Min-Quartile 25%;<br>>Quartile 25%-Quartile 50%;<br>>Quartile 50%-Quartile 75%;<br>> Quartile 75%<br>(if applicable)
8	Ipsilateral extracranial carotid tandem lesion	2	Yes;<br>No
9	Occlusion location	3	ICA;<br>M1;<br>M2
10	Collaterals	2	Grade 0-1;

	Subgroups	Number of levels	Levels
			Grade 2-3
11	Large vessel occlusion due to different etiologies	3	Intracranial atherosclerosis;<br>Cardioembolism;<br>Others
