# Supplementary material for: Asymptomatic Hemorrhagic Events and Functional Outcomes in Acute Stroke: A Secondary Analysis of the DIRECT-MT Randomized Clinical Trial
Source: JAMA Netw Open. 2025 Mar 28;8(3):e252411. doi: 10.1001/jamanetworkopen.2025.2411 (PMC11953756; doi:10.1001/jamanetworkopen.2025.2411)
Supplement: Supplement 2. — eFigure 1. Forest Plot for Stratified Analysis of Small-Volume aICH and mRS Score of 0 or 1 at 90 Days by Subgroup eFigure 2. Forest Plot for Stratified Analysis of Small-Volume aICH and mRS Score of 0 to 2 at 90 Days by Subgroup [file jamanetwopen-e252411-s002.pdf]

Supplemental Online Content

Chen R, Hua W, Zhang Y, et al. Asymptomatic hemorrhagic events and functional outcomes in acute stroke: a secondary analysis of the DIRECT-MT randomized clinical trial. *JAMA Netw Open.* 2025;8(3):e252411. doi:10.1001/jamanetworkopen.2025.2411

eFigure 1. Forest Plot for Stratified Analysis of Small-Volume aICH and mRS Score of 0 or 1 at 90 Days by Subgroup

eFigure 2. Forest Plot for Stratified Analysis of Small-Volume aICH and mRS Score of 0 to 2 at 90 Days by Subgroup

This supplemental material has been provided by the authors to give readers additional information about their work.

© 2025 Chen R et al. *JAMA Network Open*.

eFigure 1. Forest Plot for Stratified Analysis of Small-Volume aICH and mRS Score of 0 or 1 at 90 Days by Subgroup

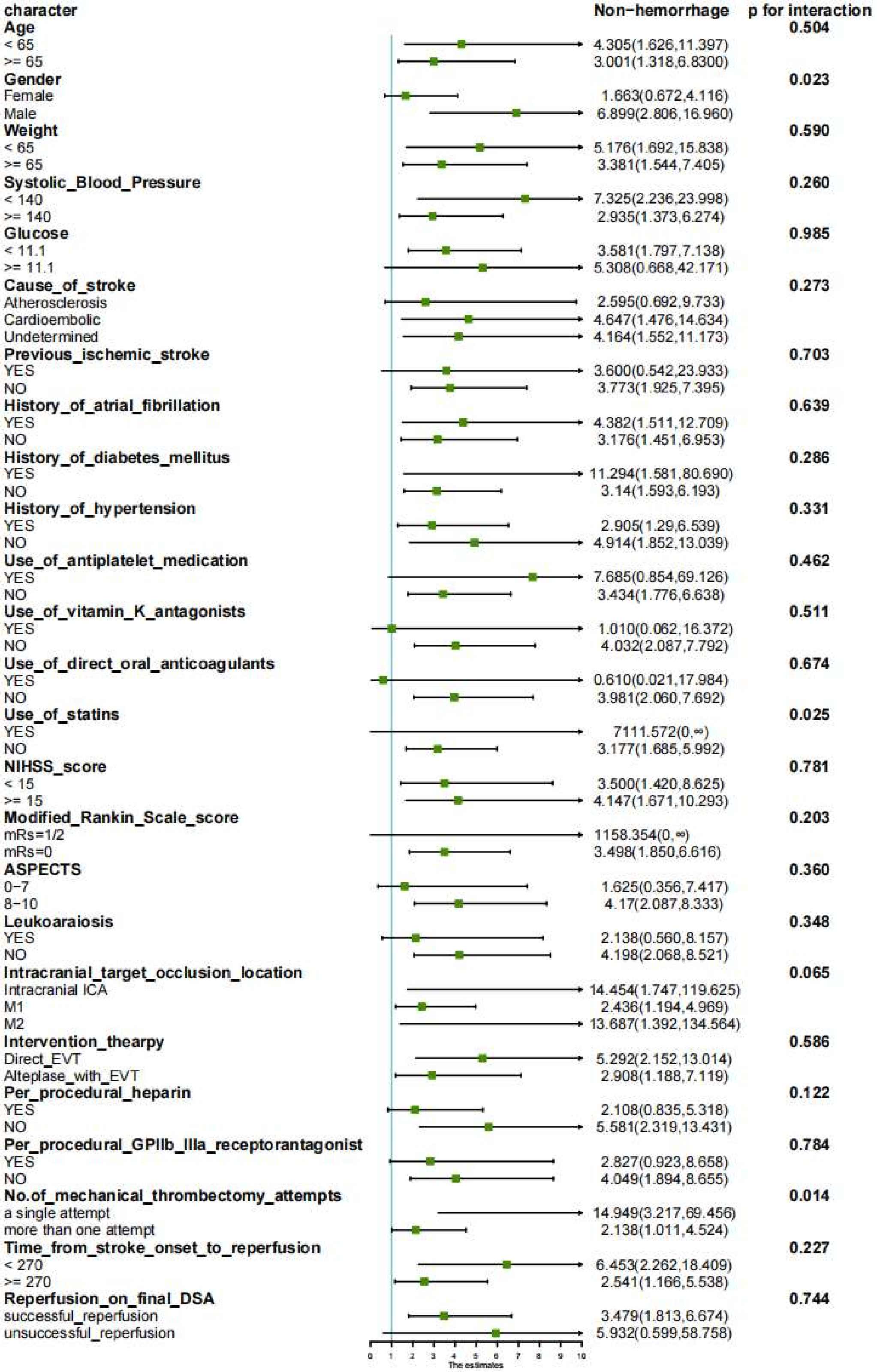

© 2025 Chen R et al. *JAMA Network Open*.

eFigure 2. Forest Plot for Stratified Analysis of Small-Volume aICH and mRS Score of 0 to 2 at 90 Days by Subgroup

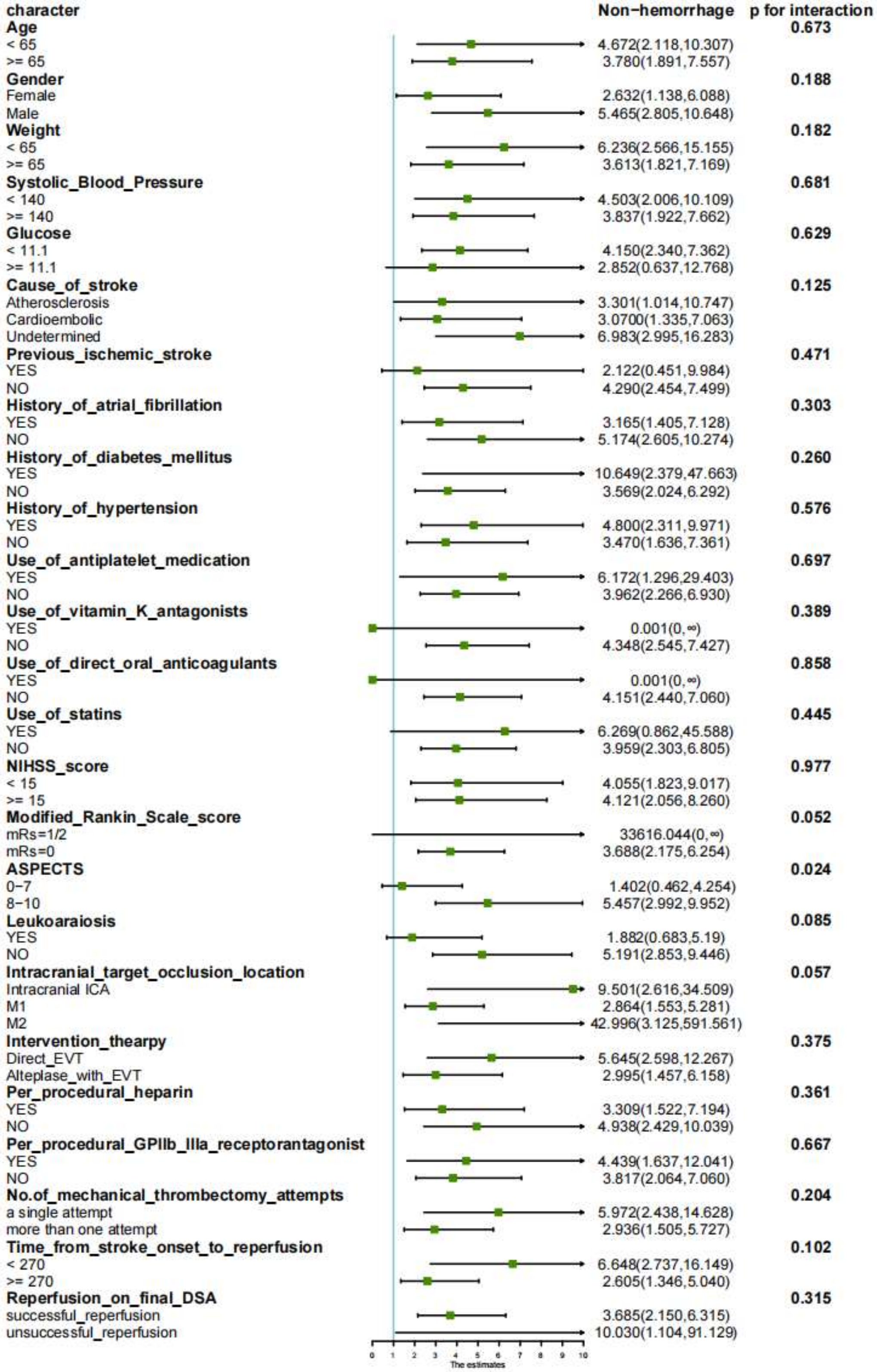

© 2025 Chen R et al. *JAMA Network Open*.
