# Supplementary material for: Asymptomatic Hemorrhagic Events and Functional Outcomes in Acute Stroke: A Secondary Analysis of the DIRECT-MT Randomized Clinical Trial
Source: JAMA Netw Open. 2025 Mar 28;8(3):e252411. doi: 10.1001/jamanetworkopen.2025.2411 (PMC11953756; doi:10.1001/jamanetworkopen.2025.2411)
Supplement: Supplement 3. — Data Sharing Statement [file jamanetwopen-e252411-s003.pdf]

Data Sharing Statement

Chen. Asymptomatic Hemorrhagic Events and Functional Outcomes in Acute Stroke. *JAMA Netw Open*. Published March 28, 2025. doi:10.1001/jamanetworkopen.2025.2411

Data
Additional Information: Trial Registry Name: Direct Intra-arterial Thrombectomy in Order to Revascularize AIS Patients With Large Vessel Occlusion Efficiently in Chinese Tertiary Hospitals (DIRECT-MT) Registry URL: https://clinicaltrials.gov/study/NCT03469206?term=NCT03469206&rank=1 Trial Registration Number: NCT03469206
Data available: No

Additional Information
Explanation for why data not available: Data are available upon reasonable request. The data used to support the findings of this study are available from the corresponding author upon request.
